# Landscape of Druggable Molecular Pathways Downstream of Genomic CDH1/Cadherin-1 Alterations in Gastric Cancer

**DOI:** 10.3390/jpm12122006

**Published:** 2022-12-03

**Authors:** Giorgio Malpeli, Stefano Barbi, Giulio Innamorati, Mariella Alloggio, Federica Filippini, Ilaria Decimo, Claudia Castelli, Roberto Perris, Maria Bencivenga

**Affiliations:** 1 Department of Surgical, Odontostomatologic, Maternal and Child Sciences, University of Verona, 37134 Verona, Italy; 2 Department of Diagnostics and Public Health, University and Hospital Trust of Verona, 37134 Verona, Italy; 3 General and Upper GI Surgery Division, Department and of Surgical, Odontostomatologic, Maternal and Child Sciences, University of Verona, 37134 Verona, Italy; 4 Section of Pharmacology, Department of Diagnostic and Public Health, University of Verona, 37134 Verona, Italy; 5 Pathology Unit, Department of Diagnostics and Public Health, University and Hospital Trust of Verona, 37134 Verona, Italy; 6 Department of Biosciences, COMT-Centre for Molecular and Translational Oncology, University of Parma, 43124 Parma, Italy

**Keywords:** E-cadherin, CDH1, gastric cancer, diffuse gastric cancer, biomarkers, pharmacological therapy

## Abstract

Loss of CDH1/Cadherin-1 is a common step towards the acquisition of an abnormal epithelial phenotype. In gastric cancer (GC), mutation and/or downregulation of CDH1/Cadherin-1 is recurrent in sporadic and hereditary diffuse GC type. To approach the molecular events downstream of CDH1/Cadherin-1 alterations and their relevance in gastric carcinogenesis, we queried public databases for genetic and DNA methylation data in search of molecular signatures with a still-uncertain role in the pathological mechanism of GC. In all GC subtypes, modulated genes correlating with CDH1/Cadherin-1 aberrations are associated with stem cell and epithelial-to-mesenchymal transition pathways. A higher level of genes upregulated in CDH1-mutated GC cases is associated with reduced overall survival. In the diffuse GC (DGC) subtype, genes downregulated in CDH1-mutated compared to cases with wild type CDH1/Cadherin-1 resulted in being strongly intertwined with the DREAM complex. The inverse correlation between hypermethylated CpGs and CDH1/Cadherin-1 transcription in diverse subtypes implies a common epigenetic program. We identified nonredundant protein-encoding isoforms of 22 genes among those differentially expressed in GC compared to normal stomach. These unique proteins represent potential agents involved in cell transformation and candidate therapeutic targets. Meanwhile, drug-induced and CDH1/Cadherin-1 mutation-related gene expression comparison predicts FIT, GR-127935 hydrochloride, amiodarone hydrochloride in GC and BRD-K55722623, BRD-K13169950, and AY 9944 in DGC as the most effective treatments, providing cues for the design of combined pharmacological treatments. By integrating genetic and epigenetic aspects with their expected functional outcome, we unveiled promising targets for combinatorial pharmacological treatments of GC.

## 1. Introduction

CDH1 (Cadherin-1 or E-cadherin) is the representative member of classical type cadherin subgroup, a broad family of membrane proteins performing calcium-dependent cell–cell homophylic interactions involved in the maintenance of a physiological tissue architecture [1]. CDH1 is involved in mechanisms regulating cell–cell adhesions, mobility, and proliferation of epithelial cells, and has a potent cancer invasion suppressing role. The function of CDH1 is essential for the stability of tight and adherens junctions between epithelial cells [2,3]. Its deficiency, or functional aberration due to mutations of the gene, results in improper localization of the adhesion molecule and alterations of junctional complexes, which is known to affect both cell–cell and cell–extracellular matrix interactions.

In normal epithelia, binding of CDH1 to CTNNB1/β-catenin assures maintenance of the epithelial integrity preventing β-catenin from being released from the cytoplasmic signaling protein complex, enter the nucleus and bind to members of the DNA binding protein family LEF/TCF [4,5,6]. TEAD4, a transcription factor driving expression of YAP/TAZ signaling target genes, is known to be regulated by LEF/TCF in organoids derived from different gastrointestinal cell types including those from stomach [7,8,9]. Thus, the selective targeting of LEF/TCF in GC cells lacking CDH1 and having an activated b-catenin pathway may represent a promising therapeutic approach for GC treatment.

A properly functioning CDH1 is critical for cell fate, whereas the lack of the protein may lead to chronic inflammation and other deleterious effects in epithelia [10,11]. In experimental models, CDH1 depletion confers a mesenchymal morphology and increased migration and invasion of the cells [10,12,13,14]. Normal gastric cells engineered with CDH1 mutations can develop organoids and turn into cell–matrix adhesion independent [15]. Conversely, ectopic expression of CDH1 in cell lines lacking endogenous expression induces reversal of undifferentiated phenotypes [16]. CDH1 loss-of-function is assumed to be an initiating event in early carcinogenesis, and it is thought to contribute to cancer progression by increasing cell proliferation and invasion ability of the neoplastic cells [17]. CDH1 re-expression observed in metastatic cells appears to help with establishing contacts with resident normal cells of the tumor microenvironment and to enhance NF-kB signaling and the metabolic support needed for metastatic growth [18].

CDH1 alterations are believed to be 20% of epigenetic nature and 10% of structural nature, and gastric cancer (GC) patients with CDH1 structural alterations displayed a significantly more dismal prognosis than patients with tumors lacking CDH1 alterations or harboring epigenetic CDH1 aberrations [19]. Mutations in the CDH1 gene have been disclosed in 33 of 395 (8.4%) cases of GC (according to the TCGA Firehose Legacy study) and are a more frequent event in the diffuse gastric cancer (DGC) subtype (20.8% of cases, TCGA Firehose legacy study). CDH1 mutation is also the major germ line genetic deficiency in the hereditary diffuse gastric cancer [HDGC], an autosomal dominant syndrome characterized by the onset of GC in young patients [20]. Hypermethylation of the CDH1 promoter has been proposed as a major second hit in advanced GC and HDGC [21,22]. Loss of CDH1 is associated with epithelial-mesenchymal transitions (EMT) in many experimental settings, including GC. Moreover, it has been reported that promoter methylation, but not CDH1 mutation, promoted EMT [23].

When all GC subtypes were compared to a normal stomach, CDH1 mRNA levels were found to be, on average, higher in GCs, but with a great variation [24,25]. Among diverse GC subtypes, lower CDH1 levels were observed in the DGC subtype, while levels higher than those found in normal gastric mucosa were present in subtypes different from DGC. Thus, this set of GC subtype-specific CDH1 profiles corroborates the idea that CDH1 loss-of-function due to gene mutation and altered expression of the transcript appear to be critical events in the onset of GC. The level of defectiveness in CDH1 expression is linked to the loss of cellular homeostasis of epithelia on a more systemic basis, and the consequences of this imbalance on its protein level need to be better understood.

In this study, computational analyses of data sets obtained from public databases were applied to identify possibly affected genes, the cellular processes that they may be associated with, and their potential dependence or impact on the CDH1 gene status to contribute to GC development. We identified discrete transcriptional signatures associated with CDH1 mutations and defined sets of genes whose mutational status is associated with differences in CDH1 transcriptional levels. Given the relevance of epigenetic regulation for CDH1 inactivation, we further correlated the association between methylation of individual CpGs and mutational status and the levels of CDH1 expression. Furthermore, we searched for protein-coding gene variants linked to CDH1 genetic alterations as potential players in pathological mechanisms underlying GC progression. Transcriptional signatures linked to CDH1 were further delineated to identify potential combinatorial drug treatment approaches.

## 2. Materials and Methods

### 2.1. Bioinformatic Analysis

Genes differentially expressed between GC cases (*n* = 372) with mutated (*n* = 34) and wild type (*n* = 338) CDH1 were retrieved from the muTarget database at https://www.mutarget.com (accessed on 13 August 2022) [26]. Running parameters for using functions “Target” and “Genotype” were *p* values ≤ 0.01 and log2 fold change cut-off >1.44. In “Genotype” analyses, a cut-off of mutation prevalence of 2% was applied. DNA methylation values of single CpGs and mRNA level of CDH1 were extracted from The Cancer Genome Atlas (TCGA), Stomach Adenocarcinoma, Firehose Legacy Study [27]. DNA methylation levels (beta values) of single CpGs were correlated with mRNA level (z-scores, RNA Seq V2 RSEM, compared to the distribution of each gene tumors that are diploid for this gene), and the Pearson’s correlation factor *r* was calculated.

### 2.2. Interactome Analyses

Interaction networks were constructed using the top 50 up- or down-regulated genes in GC cases with mutated CDH1 compared to wild type cases and the top 50 genes with CDH1 upregulation in mutated compared to wild type GC cases using the interactions available from the STRING database [28]. Interactomes were obtained using partly known physical interactions, medium confidence intervals as an interaction score, and the inflation value of 3 for clustering by MCL. Functional enrichment analyses were applied to the networks and the most significant pathways were extrapolated.

### 2.3. Pathway Enrichment Analyses

Gene enrichment analysis was performed as follows: a total of 124 gene set collections adding up to 147,592 gene sets were obtained from Enrichr at https://maayanlab.cloud/Enrichr/ (accessed on 1 December 2021) [29]. The collections were split into 13 functionally related groups [blocks] and used to perform gene set variation analysis (GSVA) [30]. Subsequently, gene sets most correlated with CDH1 gene expression were selected using the *limma* package for each block [31].

### 2.4. Patient Survival Analyses

The KM-plotter software integrates gene expression and clinical data simultaneously from TCGA [32]. To analyze the prognostic value of a particular gene, patient data were divided into two groups according to an automatically selected threshold based on the expression of a single gene or the combined expression of multiple genes. Patient survival data above or below the threshold were visualized using a Kaplan–Meier survival plot, and the hazard ratio with 95% confidence intervals and log-rank *p* value were calculated.

### 2.5. Analyses of Transcriptional Variants

Transcriptional variants differentially expressed in GC cases (*n* = 408) compared to normal stomach tissue (*n* = 36) were obtained from the database GEPIA2 (http://gepia2.cancer-pku.cn/#index; [33] (accessed on 8 February 2022)). Isoforms with a tumor/normal ratio higher than three were selected out. Subsequently, gene loci of genes with isoforms differentially expressed in GC compared to normal stomach tissue were verified on the data bank available at www.ensembl.org. Only unique (i.e., an isoform with unique amino acids numbers differing from the reference sequence) and shared (i.e., an isoform that shared the number of amino acids with other isoforms different from the reference sequence) protein-coding isoforms were considered.

### 2.6. Decoding Drugs-CDH1 Mutation Gene Expression Signatures

Differentially expressed genes identified in association with CDH1 mutations and their cognate targeting small drug compounds were matched using the L1000CDS² (L1000 Characteristic Direction Signature Search Engine) platform, a direction signature search engine of small drugs characteristics, available online: https://maayanlab.cloud/L1000CDS2/#/index (accessed on 1 September 2022) [29]. When up/down-regulated gene lists are submitted to L1000CDS², the search engine compares the input lists to the differentially expressed genes computed from the LINCS L1000 data and descriptive information on the top 50 matchings is returned. A reverse mode for the gene-set search was applied. The input upregulated genes were intersected with the datasets of downregulated genes, and the input downregulated genes are intersected with the upregulated genes of datasets. This approach allows for the screening of treatments based on the ability to counteract transcriptional changes detected in cancer cells.

### 2.7. Statistical Evaluations

The statistical approaches adopted by the databases cBioPortal, Enrichr, muTarget, KM-plotter, STRING, GEPIA2, and MSigDB are described in the corresponding portals. Statistical evaluation of differences in relative gene expression and DNA methylation levels (beta value) between patient/GC subtype groups was performed using two-tailed Student’s *t*-test. The correlation between transcripts levels and between DNA methylation degrees and gene expression levels was analyzed by Pearson correlation. If not otherwise specified, the significance level was set at *p* < 0.05.

## 3. Results

### 3.1. Gene Expression Signatures Associated to CDH1 Mutation in GC

To provide predictive information on how CDH1 loss-of-function may affect the molecular mechanisms involved in gastric carcinogenesis, we compiled RNA-seq data from 372 GC tissues by taking advantage of the software tool *muTarget*. Namely, we first selected differentially expressed genes between the 34 cases with mutated CDH1 and 338 wild type cases. Nine hundred and eighty-three genes were upregulated in tumors with CDH1 mutation (log2 fold change (FC) > 1.44, *p* < 0.01). Table 1 lists the 50 most upregulated genes, along with indications on the function traits of their coded proteins or their predicted roles in GC. Twenty-two differentially expressed genes have previously been characterized in GC as biomarkers. In contrast, no major involvement in GC has been reported yet for 28 genes.

According to GSEA analysis, epithelial-mesenchymal transition (*p* = 2.3 × 10^−8^) and myogenesis (*p* = 1.2 × 10^−2^) were signaling pathways enriched among upregulated genes. Among these, CYP1B1, COL6A2, CCDC8, CFD, and ASPN have previously been implicated in the response to drug treatments in GC [41,57,58,59,60]. CYP1B1 is a cytochrome P450 enzyme known to regulate transcription factors and allows cancer cells to reduce the toxicity of drugs by metabolizing a variety of pre-carcinogens and anticancer drugs [61]. As for CCDC8, Jumonji domain-containing protein 2A (JMJD2A) histone lysine demethylases knockdown in GC cells is associated with a significant decrease in the expression of pro-apoptotic coiled-coil domain containing 8 (CCDC8), a downstream target and interactor of JMJD2A. CCDC8 inhibition restored drug resistance to docetaxel, cisplatin, and S-1 in GC cells [41]. As for CFD (Complement factor D), it was among low-expressed genes up-regulated after vorinostat treatment of GC cells [62]. As for ASPN, it supports HIF1α-mediated resistance to oxidative stress via reprogramming of glucose metabolism, and activation of CD44-Rac1 and MMP9 to promote cell migration and invasion in GC cells [60].

Three-hundred and two transcripts could be identified as being downregulated in 34 CDH1-mutated vs. 338 CDH1-wt GC cases (FC > 1.44, *p* < 0.01). The list of the top 50 downregulated genes is reported in Table 2. The proteins LINC00511, HSD17B7, TOP2A, RFC3, EFNA1, CSE1L, TPX2, ATAD2 UBE2C, TMEM14A, PDRG1, AURKA, MTBP, RAD54B, GAD1, KRT80, KIF14, and CKS1B were previously characterized in GC. In contrast, the specific function of GC in 33 genes is unknown. According to the GSEA database, cholesterol homeostasis (*p* = 6.2 × 10^−10^), G2-M checkpoint (*p* = 6.2× 10^−7^), E2F targets (*p* = 6.2 × 10^−7^), mTORC1 signaling (*p* = 1.0 × 10^−5^), and mitotic spindle (*p* = 1.5 × 10^−3^) were enriched signaling pathways. Moreover, the genes LINC00511, TOP2A, TPX2, ATAD2, UBE2C, AURKA, MTBP, RAD54B, GAD1, KRT80, and KIF14 have previously been found as activated in response to drug treatments in GC cells.

### 3.2. Impact of Gene Mutations on CDH1 Transcription in GC

The muTarget database was searched for mutant genes related to CDH1 expression levels. Only the CDH1/Cadherin-1 transcript itself was significantly downregulated in cases harboring CDH1 mutations (*p* = 1.2 × 10^−4^). In contrast, mutation of 108 genes was associated with CDH1 upregulation (FC > 1.44; *p* < 0.01).

The top 50 genes whose mutation results in CDH1 upregulation are listed in Table 3. For each gene, we annotated the function of the protein and/or its previous involvement in the GC. No signaling pathway was significantly enriched based on GSEA hallmarks. Instead, according to the database Bioplanet 2019, these genes were enriched with the “Validated nuclear estrogen receptor beta network” term (*p* = 8.5 × 10^−4^). Note the presence of the SWI/SNF and BRG1/BAF chromatin-remodeling complexes members SMARCB1 and SMARCA4. The genes PCDHA7, CD93, SGSM1, TRPC7, ADAMTSL4, TAOK1, TADA2B, PABPC3, ATP8B1, ADAM22, POU5F2, P3H1, KCNV2, FAM193A, MOCOS, UBA6, KIAA1683, CDH16, DHX35, SMC3, RBM22, IFFO, KIAA0556, TBPL2, DIAPH1, LPCAT3, ELP3, ZFP57, EEF1D, GABBR2, NR0B2, ATN1, HPR, ECT2L, CYP2U1, and ITPRID2 have not previously been associated with GC and are therefore a novel array of putative GC markers and/or therapeutic targets whose mutation could enhance CDH1 expression.

### 3.3. Gene Enrichment Analysis of Transcripts Positively Correlating with CDH1/Cadherin−1 Expression Levels in GC

Gene enrichment analysis of transcripts positively correlating with CDH1/Cadherin−1 expression levels in GC was performed using 124 libraries available from Enrichr, including a total of 147,592 gene sets. These were arranged into 13 related groups to allow for easier computation and visual representation. For each group, gene set variation analysis (GSVA) transformation was applied and a heat-map was generated, showing the gene set expression levels in relation with the WHO, EpVsMp, and TCGA GC classifications of the examined cases, together with the CDH1/Cadherin−1 mRNA expression levels and gene mutation (Figure S1A–O). Moreover, we correlated the expression of each gene set with expression levels of CDH1. The top 50 more significantly enriched gene sets from all the groups are shown in Table 4. Among those there were: mouse SNAI2, human EHF tf ARCHS4 co-expression, human TFCP2L1 tf ARCHS4 co-expression, Human OVOL2 tf ARCHS4 co-expression, Ago2 Sufficient Pancreatic Lsl GSE147781 1, Kras Rank Pneumocytes GSE81670 1, and ERBB3 human kinase ARCHS4 coexpression. KLF5 human coexpression tf ARCHS4, ELF3 human coexpression tf ARCHS4, Flk1 + Mesodermal cell: pluripotent stem cell.

### 3.4. Sonic Hedgehog (Shh) and Collagen-Linked Pathways, Cholesterol, and Humoral Metabolism Are Key Pathways in the Interactome of CDH1-Related Genes

To dig out network of interactions of CDH1/Cadherin−1, we represented the relationships among proteins listed in Table 1, Table 2 and Table 3 (STRING, physical interactions, interaction score: medium confidence; MCL clustering: inflation 3). The interactome based on proteins of Table 1 (proteins upregulated in cases harboring CDH1 mutation) generated 7 clusters (Figure S2A). The core cluster included members of the sonic hedgehog (Shh) pathway GLI1, GLI2, GAS1, BOC, and BCL2. The Shh pathway is known to be active during embryonic development, in inflammation of the stomach mucosa, and represents a paradigm of the onset of GC [102,103]. Another significant cluster included a number of collagen genes. The interactome of the proteins of Table 2 (proteins downregulated in cases with CDH1 mutation]) generated nine clusters (Figure S2B). The three main clusters were rRNA processing, cholesterol synthesis, and steroid metabolism, and complex DNA replication factor C pathways. Cholesterol and hormone metabolism have gained importance in GC pathology [104,105]. The interactome of proteins listed in Table 3 generated seven clusters (Figure S2C). The core cluster included CDH1, chromatin-remodeling proteins SMARCB1 and SMARCA4, and connections with TADA2B and TBPL2. SMARCB1 and SMARCA4 are members of the large ATP-dependent chromatin remodeling complex SNF/SWI, which is required for transcriptional activation of normally repressed genes [106,107,108]. TBPL2 is a transcription factor required in the complex with TAF3 for the differentiation of myoblasts into myocytes. The complex TBPL2(TLF3)-TAF3 substitutes TFIID at specific promoters at an early stage of the embryonic differentiation process [109]. TADA2B is subunit of a chromatin-modifying complex SAGA, a central regulator of pluripotency, cell survival, cell growth, and lineage specification [107]. The functions of TADA2B and TBPL2 in GC are unknown.

### 3.5. Gain of Copy Number Is a Common Feature of Genes Upregulated in GC Cases Having wt CDH1 Sequence

The classification of GC recognizes tumor subtypes DGC, mucinous stomach adenocarcinoma, signet ring cell carcinoma, papillary adenocarcinoma, gastric adenocarcinoma, and tubular gastric adenocarcinoma, each one with peculiar phenotypic and molecular characteristics [110]. We illustrated the expression levels and status of 48 of the 50 genes listed in Table 2 in 485 GC cases divided into 6 GC subtypes (Figure S3). Genes downregulated in CDH1-mutated GC cases are expressed at higher level in subtypes different from DGC. This is expected as CDH1 mutations are detected in 36% of DGC, which accounts for about 15% of all GC cases.

There is clear positive dependence between CDH1 mRNA levels and gene copy number (Figure S4). Most of the mutations of CDH1 occurred in cases with a CDH1 diploid genetic status and at a higher frequency in the DGC subtype compared to other GC variants. Forty-eight of the top fifty genes upregulated in CDH1-mutated GC cases and listed in Table 1 showed copy number variation (CNV) event in at least one GC case, indicating that CNV contributes to set up their transcriptional level. We asked how genetic alterations of the 48 genes matched the CDH1 mutations in the DGC subtype compared to all GC; 39 genes were gained, and 9 genes were lost (gains 81.2% observed vs. 55.6% predicted, *p* = 0.077). Overall, these results, although up to the statistical threshold, indicate a trend of genes upregulated in CDH1-mutated GC cases to be gained in GC, belonging to subtypes different from DGC.

### 3.6. CDH1 Mutation in DGC Entails Gene Enrichment of Bmp2, Stem Cell, and H3K27me3 Pathways for Upregulated Genes and DREAM Complex, Targets of EWSR1-FLI1 Fusion up and BRCA1 PCC Network Pathways for Downregulated Genes

To assess the impact of driver CDH1 mutations in the context of the DGC subtype, we compared the transcriptional profile in 13 CDH1-mutated and 56 CDH1-wild type DGC cases. We found 969 upregulated genes and 588 downregulated genes in CDH1-mutated versus CDH1-wild type DGC cases (FC > 1.3, *p* < 0.05). The 10 most significantly upregulated genes included PTH1R, AUTS2, NALCN, LPL, FXYD1, DRP2, PTGDR, WMS, LHFP, and FZRB, whereas the 10 most significantly downregulated genes were FAM199X, ATP13A3, MASTL, DTX3L, PARP9, PSMD12, DNAJB11, HNRNPF, CSTF1, and PSMD11. The literature does not report direct implication in DGC of these 20 genes. Interestingly, DTX3L and PARP9 are known to cooperate in promoting the rapid and specific recruitment of 53BP1/TP53BP1 and UIMC1/RAP80 complexes and BRCA1 into DNA damage sites [111,112]. In virtue of its implication in DNA repair, the DTX3L/PARP9 complex could be considered a promising target of combinatorial drug treatment against CDH1-mutated DGC cancer.

By gene enrichment analysis accessible at GSEA, it was possible to make predictions on the cellular functions in which upregulated and downregulated genes could be involved (Table S1). Upregulated genes were mainly enriched in gene sets Lee, Bmp2 targets up (2.7 × 10^−45^), Boquest, stem cell up (3.6 × 10^−42^), Benporath, genes with H3K27me3 (2.0 × 10^−41^), Lim, mammary stem cell up (1.1 × 10^−39^), Naba, and matrisome (7.2 × 10^−34^). Downregulated genes were mainly enriched in the gene sets Fischer, DREAM targets (4.8 × 10^−146^), Kinsey, targets of EWSR1-FLI1 fusion up (4.3 × 10^−121^) Dodd, nasopharyngeal carcinoma down (6.2 × 10^−117^), Pujana, BRCA1 pcc network (normal tissues) (8.1 × 10^−114^), Gobert, oligodendrocyte differentiation up, and hay bone marrow erythroblast (1.6 × 10^−108^).

In summary, the signaling pathway analysis on CDH1-related transcriptional signatures in DGC highlights strong and significant implication of features of stem cells, histone methylation and mechanisms, and apparatus controlling cell cycle and cell differentiation.

### 3.7. Protein-Encoding Isoforms of Genes Associated with CDH1 Genomic Alterations in GC

We investigated the potential impact of mutation or altered expression of CDH1 and the differentially expressed isoforms (DEI) when comparing GC and normal gastric mucosa for the genes listed in Table 1, Table 2 and Table 3. DEI in GC compared to normal tissue were extracted through the GEPIA2 software tool. Next, DEIs were examined in the context of their own gene locus as reported in the database Ensembl, and the specific annotations are listed in Table 5.

To highlight DEIs encoding proteins with unique characteristics, DEI representing non-coding RNAs and transcripts coding for the protein having an amino acid length equivalent to that of the reference isoform were not considered. Among the remaining DEI, we selected out those encoding for unique or shared proteins (meaning an isoform with an alternative amino acid common to another gene isoform). These include isoforms of genes, ASPN, CCDC80, COL16A1, COL6A3, FBLN2, and PRRX1, related to the ones reported in Table 1; of genes, AURKA, GGH, PRMT3, RAE1, and UBE2C, related to those listed in Table 2; of genes, ANXA4, ARHGEF28, DSC2, EEF1D, ELP3, EPB41L1, FBXO38, KIF18B, MAPK4A, MUC20, P3H1, SGCE, TROAP, UBA6, and USP22, related to the gene set of Table 3. These protein variants represent potential mediators of the deregulation of cellular processes following CDH1 loss and therapeutic targets.

### 3.8. CDH1 Transcriptional Levels and Degree of DNA Methylation Are Inversely Correlated in GC Subtypes

Hypermethylation of the CDH1 gene is a recognized secondary event to the gene mutation, thus contributing synergistically to CDH1 inactivation in different cancer types [22]. To further understand the influence of DNA methylation on CDH1 gene inactivation in GC, we compiled the extent mRNA and DNA methylation levels in 377 GC cases which were subdivided into five subtypes. CDH1 transcript levels were higher in non-mutated compared to mutated tumors (average RNA Seq V2 RSEM, 0.02 vs. −0.64, *t*-test *p* = 0.0014). Considering probes with the strongest negative correlation between the DNA methylation and mRNA expression levels in all GC cases, CDH1 DNA methylation was higher in CDH1-mutated cases compared to CDH1-wild type cases (average beta value 0.39 vs. 0.29; *p* = 1.2 × 10^−5^).

DNA methylation and mRNA levels of CDH1 were inversely correlated considering all GC cases (r = −0.55, *p* = 3.2 × 10^−31^) (Figure S5). At single CpG level, the most significant inverse correlation between DNA methylation and mRNA expression in mutated GC tumors (*n* = 29) was disclosed for CG dinucleotides cg26508465 (r = −0.60, *p* = 0.0006), cg07762788 (r = −0.56, *p* = 0.0016), cg09406989 (r = −0.53, *p* = 0.0031), and cg24765079 (r = −0.42, *p* = 0.023). By contrast, a direct correlation could be evidenced for cg06875305 (r = 0.56, *p* = 0.016).

Inverse correlation of DNA methylation and mRNA expression levels was significant and similar in GC subtypes: DGC (r = −0.45), mucinous stomach adenocarcinoma (r = −0.62), Signet Ring cell carcinoma (r = −0.59), papillary adenocarcinoma (r = −0.45), gastric adenocarcinoma (r = −0.50), and tubular gastric adenocarcinoma (r = −0.45). These results indicate that DNA methylation may be implicated in the regulation of CDH1 transcription, and its increase concurring with CDH1 mutations might contribute to the loss of gene expression.

### 3.9. A Set Comprising 50 of the Most Upregulated Genes in CDH1-Mutated GC Cases Associate with Lower Overall Survival of GC Patients

**Figure 1:**
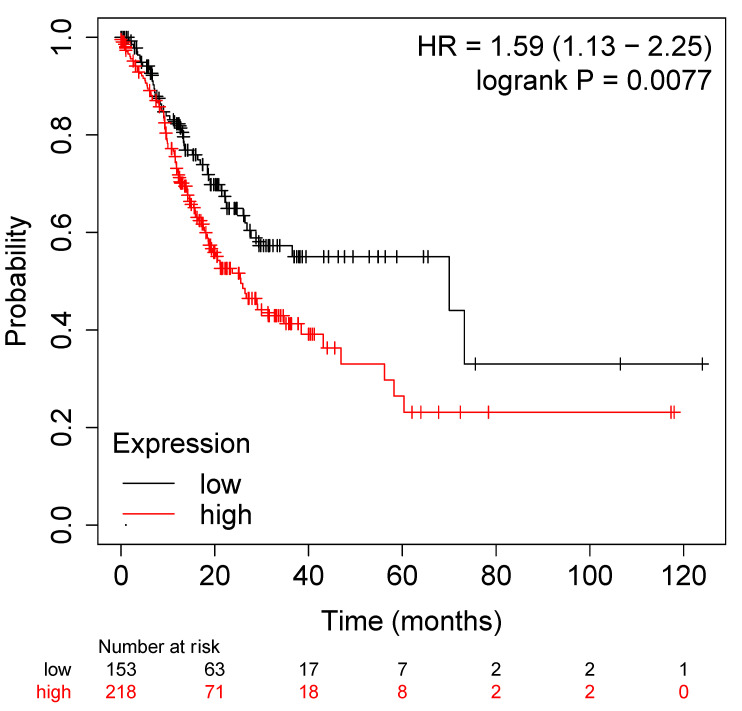
Overall survival in 371 GC cases based on the average mRNA level of 50 genes associated with higher CDH1 level in CDH1-mutated compared to CDH1-wt cases (Table 1). The threshold was selected automatically by the software. The image can be reproduced at https://kmplot.com/analysis/ (accessed on 6 May 2022) using the best cut-off option.

We next investigated the possible association of transcript levels of the genes listed in Table 1, Table 2 and Table 3 with the overall survival (OS) in a cohort of 371 GC patients. When considering the 50 most upregulated genes (Table 1), a reduced OS characterized cases with a higher mRNA level (218 cases exhibiting higher transcriptional levels) showed lower OS rate than the 153 cases presenting lower expression levels (*p* = 0.0077) (Figure 1). The three genes most significantly associated with OS were CLMP (*p* = 0.00028), GLT8D2 (*p* = 0.00079), and COLEC12 (*p* = 0.0012).

No significant association with OS rates was observed for the whole genes of Table 2 (upregulated in CDH1-mutated cases) and for the whole genes of Table 3 (gene mutation concomitant to CDH1 upregulation). As for single genes reported in Table 2, lower mRNA expression levels of MTBP (*p* = 0.0029), KIF14 (*p* = 0.0046) and SNHG1 (*p* = 0.0051), ATAD2 (*p* = 0.0095), GGH (*p* = 0.011), RAB5IF (*p* = 0.024), GAD1 (*p* = 0.026), CKS1B (*p* = 0.032), TPX2 (*p* = 0.033), LINC00511 (*p* = 0.04), CHKA (*p* = 0.04), RAD54B (*p* = 0.043), and MAL2 (*p* = 0.048) was associated with reduced OS. As for single genes of Table 3, higher levels of CYP2U1 (*p* = 0.00066), FGF21 (*p* = 0.004), TAOK1 (*p* = 0.0051), CD93 (*p* = 0.0081), HPR (*p* = 0.015), PCDHA7 (*p* = 0.017), P3H1 (*p* = 0.018), MAP4K4 (*p* = 0.018), BRINP3 (*p* = 0.026), ZNF418 (*p* = 0.032), ADAM22 (*p* = 0.032), NETO2 (*p* = 0.036), DHX35 (*p* = 0.047) associated with less OS. Lower level of genes SMARCA4 (*p* = 0.0075), DIAPH1 (*p* = 0.024), GAD1 (*p* = 0.026), XAB2 (*p* = 0.028), ECT2L (*p* = 0.033), TADA2B (*p* = 0.041), EEF1D (*p* = 0.046), SMC3 (*p* = 0.047), and MAP3K6 (*p* = 0.047) were associated with less OS.

### 3.10. Therapeutic Implications of CDH1-Associated Transcriptional Changes in GC

**Figure 2:**
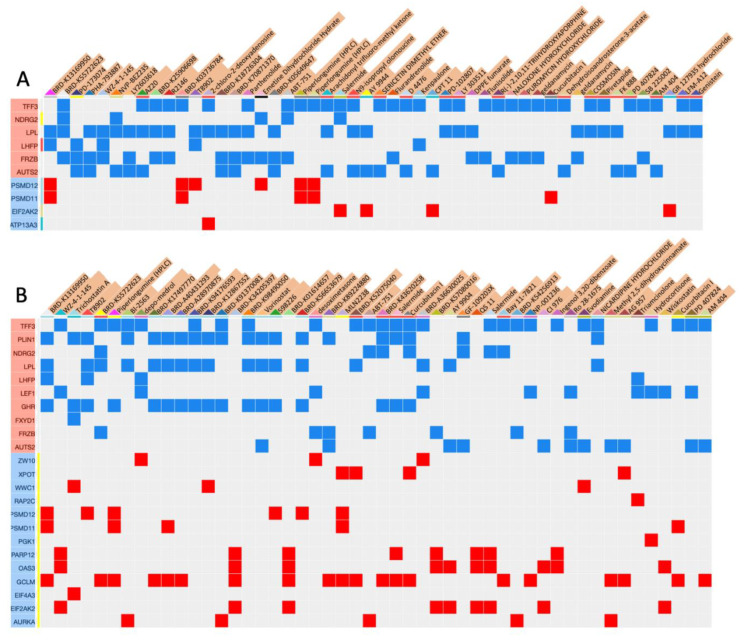
Matching drugs and consensus genes based on twenty differentially expressed genes from Table 1 and twenty genes from Table 2. Consensus for small molecule matching signatures in GC (**A**) and in DGC (**B**). On the Y side bar are up, light red, or down, light blue, regulated genes; on the X bar are drugs used for treatments. The input gene signature is depicted as the rows of the matrix and the expression level of the genes are indicated with light red or light blue label-bars. The top-ranked L1000 perturbations (e.g., those with most similar or anti-similar signatures) are shown as columns with light red label-bars that indicate their score. L1000 perturbation gene signatures are depicted as columns of the matrix with red and blue squares, indicating their effect on gene expression.

We searched for potential targets of pharmacological treatments in GC by investigating the intersection between differentially expressed genes in response to treatments with drugs and genes connected with mutated CDH1. To this end, we selected the twenty most upregulated and the twenty most downregulated genes in CDH1-mutated versus non-mutated GC and DGC cases and submitted these gene sets to the server L1000CDS² to identify matched consensuses. To profile the molecular and phenotypical outcomes of agent perturbed human cells, the differentially expressed genes of these profiles were calculated using the characteristic direction method. The score obtained for a single agent measuring the overlap combines upregulated and downregulated genes (Table S2). In Figure 2A,B we report drugs that associate with the most upregulated and downregulated genes in all GC and in DGC, respectively.

Drugs showing the highest scores were FIT (0.1667), GR 127935 hydrochloride (0.1667), amiodarone hydrochloride (0.1667) in all GC and BRD-K55722623 (0.222), BRD-K13169950 (0.222), and AY 9944 (0.1667) in DGC. The genes that could be predicted to be best co-targeted by selected treatments in all GC were TFF3, NDRG2, LPL, LHFP, FZRB, and AUTS2 in the case of upregulation, and PSDM12, PSDM11, EIF2AK2, and ATP13A3 in the case of downregulation. The genes that could be predicted to be best co-targeted by top four treatments in DGC were TFF3, PLIN1, NDRG2, LPL, LHFP, LEF1, GHR, FXHD1, FZRB, and AUTS2 in the case of upregulation and ZW10, XPOT, WWC1, RAP2C, PSDM12, PSDM11, PGK1, PARP12, OAS3, GCLM, EIF4A3, EIF2AK2, and AURKA in the case of downregulation. Drug combinations that best matched upregulated and downregulated genes in CDH1-mutated versus non-mutated cases were GF-109203X/BRD-K37392901 (0.333), BRD-K54256913/BRD-K37392901 (0.333), BRD-K11634954/BRD-K37392901 (0.333) for GC and BRD-K55722623/BRD-K13169950 (0.389), BRD-K13169950/Salermide (0.389), and BRD-K13169950/ABT-751 (0.389) for DGC (Table S3).

## 4. Discussion

We delineate here a landscape of genetic, epigenetic, and cellular pathways linked to genetic and transcriptional alterations of CDH1 in GC. We performed multiple transcriptional comparisons to reveal relationships occurring between CDH1 and interacting genes in GC, considering all histological variants. Particular consideration was dedicated to the DGC subtype, where loss-of-function or mutation of the gene is a frequent event. The findings intersect aspects previously annotated and new insights into the interactome sustaining GC progression. Transcriptional data were finally computed to disclose relevant druggable targets for GC treatment.

Transcriptional profile changes associated with CDH1 mutations in GC highlighted enrichment of EMT and myogenesis-related signaling pathways. In particular, the upregulation of ZEB2 and TWIST2 stood out, alongside the increased expressions of GAS1, GLI1/2 and POU2AF1, known to characterize EMT and stem cells [113,114]. Of note in this context is that Hedghog autocrine signaling has been shown to promote GC cell proliferation [115], suggesting that CDH1 loss-of-function and concurrent activation of the Hedghog signaling pathway may cooperate in potentiating the loss of the epithelial phenotype [116]. It could then be deduced that targeting GLI proteins may be a reasonable counter-measure to halt GC progression [117].

Among the genes found to be upregulated in CDH1 mutated cases and previously studied in GC, CYP1B1, COL6A2, CCDC8, CFD and ASPN have been proposed to be implicated in the response to drug treatments [41,57,58,59,60,61,62]. CYP1B1 is a cytochrome P450 enzyme known to regulate transcription factors and to reduce drug toxicity by metabolizing a variety of drugs including pre-carcinogenic and anticancer drugs [61]. Thus, suppression of CYP1B1 is known to synergize with anticancer drugs [118]. Similarly, enhanced deposition of collagen type VI in the tumor microenvironment has been experimentally shown to contribute to an augmented drug resistance in different types of carcinomas.

The role of 28 of the 50 genes upregulated in CDH1-mutated cases, including COL16A1, OMD, S1PR2, CLMP, KCNE4, CCDC80, FMNL3, VSTM4, PCDHGB7, CILP, POU2AF1, SERPINF1, GGT5, THBS4, GLIPR2, COL8AT2, RFRAT2, RFRAT2 GLT8D2, MAP1A, FBLN2, BOC, LSP1, PLEKHO2, TMEM273, OLFML1, SC5D, and NDN, remains unresolved in the context of GC. Thus, these genes provide an ample array of novel potential diagnostic and therapeutic targets to explore, singly or in combination. Moreover, upregulation of 9 of the 50 genes, including COL16A1, EMILIN1, CLMP, COL6A2, CCDC80, FMNL3, PCDHGB7, COL6A3, COL8A2, strongly support the idea that accumulation of collagenous and ECM glycoproteins in the GC microenvironment may strongly affect the progression of the tumor and ultimately impact on its metastatic behavior.

Genes downregulated in CDH1-mutated GC cases were enriched in pathways and factors primarily related to cholesterol homeostasis, G2-M checkpoint, E2F targets, mTORC1 signaling, and mitotic spindle formation and maintenance. Deregulation of cholesterol, steroid and lipid metabolism has been previously reported in GC [119]. The SQLE gene, encoding the enzyme squalene epoxidase that catalyzes the rate-limiting phase in cholesterol biosynthesis, and DHCR7 and IDI1, two additional enzymes that operate in the cholesterol biosynthesis pathway, were noted to be significantly downregulated. These findings would suggest that treatments aimed at modulating cholesterol metabolism could be beneficial for GC patients.

We identified genes whose genetic status, i.e., wild type or mutated, differentially associated with CDH1 transcriptional levels, and could be directly or indirectly implicated in the transcriptional regulation of CDH1. Examples of such genes are SMARCA4 and SMARCB1, two members of the SWI/SNF chromatin remodeling complex. Considering all the protein subunits forming the complex, the GCs cases harboring mutated SWI/SNF showed a more favorable outcome [120]. Furthermore, although SMARCA4 had the highest mutation rate (6%) among gene-characterizing cell senescence, it was linked to higher overall survival and progression-free survival [121]. Taken together, these results suggest that progression of GC cells into a malignant phenotype requires an active and well-functioning chromatin remodeling apparatus to allow transcriptome reshuffle.

We further found that mutation of 108 genes associated with CDH1 upregulation and a corollary gene enrichment revealed a major involvement of the estrogen receptor beta (ERβ network). The function of ERβ has been investigated in GC [122,123], but no relationship with CDH1 status has previously been reported. In breast cancer, impaired activity of ERβ affects the oncogenic activity of NF-kB by inducing outplacement of the EZH2/PRC2 complex and transcriptional repression [124]. We argue that higher levels of CDH1 may cooperate with gene mutation events implicated in the driving of NF-kB and PRC2 functions. Overall, we propose that CDH1 might act as a modulator of the metabolism and physiological role of cholesterol and humoral pathways to promote the progression of GC. In support of this hypothesis, we found that a higher transcriptional level of genes upregulated in CDH1 mutated GC cases was associated with worse OS.

The outcome of our computational analysis suggests that transcriptional levels of CDH1 are dictated by its genetic status and discriminate the most genetically unstable cases of GC. CDH1 mRNA levels and promoter methylation were disclosed to be inversely related in different GC subtypes. Furthermore, we observed that hypermethylation of CDH1 occurs preferentially in the CDH1-mutated GC. These observations confirm that CDH1 methylation controls gene transcription in GC, as previously reported [21,22], and that genes upregulated in CDH1-mutated GC cases possess a higher gene copy number than CDH1-mutated cases. This would mean that epigenetic and genetic mechanisms help to precisely define the transcriptional pattern highlighted in GC.

When we examined the transcriptional context of CDH1 mutations in DGC we could conclude that mutation of the CDH1 gene was a more frequent event in this latter GC subtype. Transcriptional signatures linked to mutated CDH1 in the DGC subtype feature upregulation of genes involved in BMP signaling, stem cells, and the DNA methylation mark H3K27me3, alongside down-regulation of genes controlled by the DREAM complex, targets of the EWSR1-FLI1 gene fusion product, and genes involved in the BRCA1-PCC network. Curiously, BMP2 stimulation has previously been shown to promote motility and invasion of GC cells [125], while the acquisition of stem cell properties in the context cell transformation is believed to be promoted through various mechanisms including CDH1. Third, epigenetic reprogramming mediated by the chromosome mark H3K27me3 has been demonstrated to contribute to epithelial cell dedifferentiation and the subverting of the transcriptional program of healthy epithelial cells. Accordingly, both EZH2, the catalytic subunit of the Polycomb-2 repressive complex, and the H3K27me3 mark predicted poor survival of GC patients [126].

Among the down-regulated genes evidenced in CDH1-mutated DGC, components of the DREAM complex emerge as specifically modulated. This characteristic of the gene pattern clearly recalls an aberrant regulation of the cell cycle in the neoplastic cells driven by the DREAM complex. This multiprotein complex, which includes the dimerization partners of Rb, E2F proteins, and MuvB, and which also cooperates by linking to cell-cycle regulatory factors such as p130, p107, BMYB, and FOXM1, is a major controller of cell division. These multiprotein complexes are known to orchestrate cell quiescence in normal cells and are deregulated in numerous cancer types [127]. To what extent aberrant expression of the DREAM complex in DGC directly or directly affects the formation and the evolution of this GC subtype remains to be determined. It could, however, be implied that a network linking CDH1, the DREAM complex, and cognate effectors acting downstream on chromatin remodeling and transcriptional regulation could represent a functional axis to be further investigated in DGC.

By applying an analysis of transcriptional patterns to delineate specific and characterizing gene profiles across all GC subtypes, we were able to highlight gene networks involving enriched signaling pathways. In addition to the EMT transcription inhibitors SNAI2 and ZEB1, the transcription factors EHF, TFCP2L1, OVOL2, GRHL2, KLF5, ELF3, MST1R, RUNXs, OVOL1, ZBTB7C, and LGR4 were consistently co-expressed with CDH1. To put these genes in the right context, it is notable that EHF has been identified as a HER2-regulated transcription factor [128], whereas OVOL1/2 are known to function as promoters of EMT in cancer [129]. KLF5 is reported to act as a promoter of cell proliferation [130], ELF3 is essential for maintenance of the epithelial phenotype [131], and MST1R has been associated with enhanced growth of GC cells [132]. RUNX2 has been found to fuel YAP1 signaling and GC tumorigenesis [133], whereas ZBTB7C is a recognized gluconeogenic transcription factor that is still unexplored in GC [134]. LGR4 has been proposed to promote proliferation of both GC and gastric mucosa cells and may therefore function as a cell division switch in neoplastic conditions [135]. Virtually nothing is known about the possible role of TCFP2L1 and GRHL2 transcription factors in the context of GHC. Therefore, in light of the present findings, it may be worth pursuing dedicated studies of these factors.

We used the CDH1 mutation as a probe to select a subset of gene variants differentially expressed in GC compared to normal stomach tissue. The computational approach was specially aimed at identifying variants encoding proteins with a unique amino acid sequence clearly differing from the reference gene sequence. An important set of protein isoforms coded by the genes ASPN, CCDC80, COL16A1, COL6A3, FBLN2, PRRX1, AURKA, GGH, PRMT3, RAE1, UBE2C, ANXA4, ARHGEF28, DSC2, EEF1D, ELP3, EPB41L1, FBXO38, KIF18, KIF3, TROAP, UBA6, and USP22 was identified, thereby providing leads to novel potential diagnostic markers and/or therapeutic targets. Interestingly, ASPN has been shown to induce cellular reprogramming through increased resistance to oxidative stress in GC cells [38]. Similarly, CCDC80 has been associated with acquired drug resistance and immune infiltration in colorectal cancer [136]. To our knowledge, no studies have specifically addressed the significance of these proteins in GCs, but it could be expected that similar pro-tumorigenic function could be exerted in these tumors.

Transcriptional peculiarities linked to misexpression of CDH1 could indeed be exploited to design new and more effective drug treatments of GC and the DGC subtype. Through our pharmacologically directed computational approach we identified drug treatments that, when associated with the 50 most up- and down-regulated genes, highlighted FIT, GR 127935 hydrochloride, GF-109203X, BRD-K54256913, BRD-K94390040, BRD-K94832621, BRD-A10420615 BRD-K11634954 among the small drug compounds as showing the highest efficiency scores and never cited in relation to the GC treatment.

Cumulatively, we predict that the protein products of the upregulated genes GAS1, COL6A2, CILP, and ASPN may represent ideal therapeutic targets for the treatment of GC, and some background information in this context is already available for GAS1 and COL6A2. In addition, the marked down-regulation in CDH1-mutated GC cases of the proteins coded by the genes TMEM14A, MSOM1, and IDI1 appears to be particularly interesting, as it may suggest novel diagnostic markers. Molecules of particular relevance as CDH1 co-targets for therapeutic approaches on DGC include TFF3, PLIN1, NDRG2, and LPL, while the down-regulated ZW10, XPOT, WWC1, and RAP2C may serve as diagnostic markers. It naturally remains to be ascertained whether the cellular processes in which these genes operate will allow the conceiving of a rational pharmacological strategy based on targeting the single proteins or combination of these molecules within the framework of therapeutic approaches against GC and DGC.

This multilevel analysis provides an overview of the molecular events that result from CDH1 alterations. The evidence that emerged from this study represents useful working hypotheses to be experimentally tested in the context of synthetic lethality screening. To reinforce the results, the study of only one cohort should be extended to another. Although CDH1 loss is a necessary initial event in gastric carcinogenesis, re-expression of CDH1 during cancer progression could represent a confounding factor in identifying useful targets for combined drug treatments. We therefore propose that integrated analysis of genetic, epigenetic, and functional information be applied separately to cohorts that are representative of early and late GC evolution to identify stage-specific synthetic lethality mechanisms.

## Figures and Tables

**Table 1:** Listing of the significantly most upregulated genes in CDH1-mutated versus CDH1-wt GC cases.

Symbol	FC *	*p*-Value	Function in GC or in General	Reference *
TMEM119	2.44	3.0 × 10^−8^	STAT3 signaling in GC.	Zheng P et al. Onco Targets Ther 2018 [34]
COL16A1	1.83	1.4 × 10^−7^	Cell attachment and integrin-mediated cellular reactions.	Nd
OMD	3.45	1.6 × 10^−7^	Biomineralization processes. TGFbeta pathway.	Nd
PRRX1	1.96	2.2 × 10^−7^	WNT-related cofactor of EMT in GC.	Guo J Et al. Mol Oncol 2015 [35]
PCOLCE	2.05	3.0 × 10^−7^	Immune infiltration in GC.	Xiang A et al. Front Mol BioSci 2020 [36]
EMILIN1	1.98	3.0 × 10^−7^	Extracellular matrix glycoprotein interacting with microfibrils and collagens. Angiogenesis, migration, invasion in GC.	Qi Y et al. BMC Cancer 2019 [37]
CYP1B1	3.32	3.3 × 10^−7^	Cytochrome P450, aryl hydrocarbon receptor pathway in GC.	Yin XF et al. Oncol Rep 2013 [38]
S1PR2	1.47	4.6 × 10^−7^	Bile acid receptors, gastrointestinal functions.	Ticho AL et al. Liver Res 2019 [39]
GAS1	2.33	4.8 × 10^−7^	Stem cell activity in GC.	Katano T et al. Am. J. Pathol. 2015 [40]
CLMP	2.07	7.5 × 10^−7^	Cell–cell adhesion.	Nd
KCNE4	2.00	8.5 × 10^−7^	Subunit of the voltage-gated potassium channel complex.	Nd
COL6A2	1.84	8.7 × 10^−7^	Cell-binding protein.	Nd
CCDC80	2.80	9.8 × 10^−7^	Cell adhesion.	Nd
FMNL3	1.65	1.0 × 10^−6^	Required in the control of cell shape and migration. RHO-GTPase related.	Nd
VSTM4	1.98	1.0 × 10^−6^	V-Set and Transmembrane Domain Containing.	Nd
PCDHGB7	1.80	1.1 × 10^−6^	Expressed in liver and pancreatic juice.	Nd
CILP	2.53	1.1 × 10^−6^	TGFbeta and IGF1 pathway control.	Nd
CCDC8	1.68	1.1 × 10^−6^	Cofactor required for p53-mediated apoptosis. Interacts with JMJD2A.	Nagakawa T et al. Gastric Cancer 2020 [41]
CFD	2.93	1.2 × 10^−6^	Complement Factor D.	Nd
BNC2	2.41	1.2 × 10^−6^	Zinc finger protein, mutated in GC.	Yang M Arai et al. Carcinogenesis 2020 [42]
POU2AF1	1.75	1.4 × 10^−6^	Transcriptional coactivator of OCT1 or OCT2	Nd
SERPINF1	2.40	1.4 × 10^−6^	Secreted by adipocytes.	Nd
RUNX1T1	2.18	1.5 × 10^−6^	Inhibitor of CEBPB. Downregulated in 38 % of GC.	Regalo G et al. J Mol Med 2016 [43]
COL6A3	1.84	1.5 × 10^−6^	Upregulated in GC. Its inhibition blocks cancer cell proliferation.	Yang B et al. Front Genet 2020 [44]
GGT5	1.92	1.6 × 10^−6^	Inhibits steoroidogenesis. Member of the metabolic signature of GC.	Zhao Q et al. J Cancer 2021 [45]
HIC1	2.03	1.8 × 10^−6^	Transcriptional repressor in Wnt signaling.	Nd
COLEC12	2.15	1.8 × 10^−6^	Receptor involved in H. Pylori-stromal cell interaction in gastric diseases.	Chang LL et al. Sci Rep 2018 [46]
THBS4	3.54	1.9 × 10^−6^	Promotes proliferation and metastasis in GC.	Chen X et al. J Physiol Biochem 2019 [47]
GLIPR2	1.57	1.9 × 10^−6^	Glioma Pathogenesis-Related Protein 2. Protein binding in vesicle.	Nd
COL8A2	1.82	1.9 × 10^−6^	Migration and proliferation of vascular smooth muscle cells. Induced by TWIST1.	Nd
GLI1	2.29	2.0 × 10^−6^	Regulates stemness characteristics in GC.	Qi W et al. Diagn Pathol 2020 [48]
ZEB2	1.90	2.2 × 10^−6^	Transcriptional inhibitor that represses CDH1.	Park JW et al. Mol Carcinog 2018 [49]
CCL19	4.40	2.4 × 10^−6^	Expressed only in GC. Suppresses cell proliferation, migration, and invasion.	Zhou R et al. Human Cell 2020 [50]
GLI2	1.88	2.4 × 10^−6^	GLI2 induces PDGFRB expression and modulates cancer stem cells in GC. Involved in resistance to drugs in GC.	Lu Y et al. J Transl Med 2021 [51]
RFTN1	1.59	2.4 × 10^−6^	Formation and/or maintenance of lipid rafts.	Nd
ADRA2A	1.93	2.4 × 10^−6^	The stimulation of this adrenoceptor enhances gastric carcinogenesis.	Tatsuta M et al. Int J Can 1998 [52]
GLT8D2	1.86	2.6 × 10^−6^	Glycosyltransferase 8 Domain Containing 2. Synthetic lethal with Ras	Nd
TIMP3	2.06	2.7 × 10^−6^	Metalloprotease inhibitor. Tissue-specific acute response to remodeling stimuli. Marker of GC.	George S et al. Front Microbiol 2020 [53]
ASPN	2.14	2.7 × 10^−6^	Promotes cell proliferation via PSMD2. Overexpressed in DGC.	Zhang Z et al. Front Biosci 2019 [54]
MAP1A	2.00	2.8 × 10^−6^	Binding partner of RhoB. Regulator of EGFR.	Nd
FBLN2	2.26	2.9 × 10^−6^	Required for basement membrane integrity.	Nd
DTX1	2.17	3.0 × 10^−6^	Regulation of c-FLIP stability. Downregulated in GC.	Hsu TS et al. Cell Death Dis 2018 [55]
BCL2	1.92	3.3 × 10^−6^	Antiapoptotic protein never mentioned as associated to CDH1 mutations in GC.	Nd
BOC	2.70	3.4 × 10^−6^	Positively regulates hedgehog signaling.	Nd
LSP1	1.82	3.4 × 10^−6^	Lymphocyte and endothelium specific.	Nd
PLEKHO2	1.52	3.6 × 10^−6^	Promotes macrophages survival.	Nd
TMEM273	1.75	3.7 × 10^−6^	Uncharacterized protein.	Nd
OLFML1	1.95	3.7 × 10^−6^	Olfactomedin-Like Protein 1.	Nd
TWIST2	2.25	3.7 × 10^−6^	Vascuologenic mimicry. EMT in GC.	Wang L et al. Oncotarget 2015 [56]
SSC5D	2.10	3.7 × 10^−6^	Scavenger Receptor Cysteine-Rich Family Member With 5 Domains.	Nd

* FC, fold change; Nd, unknown.

**Table 2:** Listing of the significantly most down-regulated genes in CDH1-mutated versus CDH1-wt GC cases.

Gene	FC *	*p*-Value	Function in GC or in General	Reference *
C5orf34	1.85	5.2 × 10^−7^	Uncharacterized protein.	Nd
SQLE	2.13	5.7 × 10^−7^	Squalene epoxidase. Rate-limiting enzyme in steroid biosynthesis.	Nd
EIF2S2	1.47	1.0 × 10^−6^	Eukaryotic translation initiation factor 2.	Nd
TOMM34	1.64	1.2 × 10^−6^	Unfolded protein import in mitochondria.	Nd
RAE1	1.54	1.2 × 10^−6^	RNA exporting to cytoplasm.	Nd
LINC00511	2.04	1.2 × 10^−6^	Acts as a ceRNA in GC	Sun CB et al. World J Gastr Oncol 2020 [63]
HSD17B7	1.45	1.4 × 10^−6^	Hydroxysteroid 17-Beta Dehydrogenase 7. Mevalonate and sterols enzyme downregulated and associated with survival in GC.	Chang W-C et al. Cancer Med 2018 [64]
TOP2A	2.22	1.8 × 10^−6^	DNA Topoisomerase II Alpha. Associated with GC recurrence.	Terashima MI et al. Oncotarget 2017 [65]
DHCR7	1.89	1.9 × 10^−6^	Removes the C [7,8] double bond of sterols and catalyzes the conversion of 7-dehydrocholesterol to cholesterol.	Nd
DNMT3B	2.70	2.0 × 10^−6^	DNA Methyltransferase 3 Beta. Its downregulation induces stemness.	Wu S et al. Biosci Rep 2020 [66]
MAL2	1.75	2.0 × 10^−6^	Marker of spasmolytic polypeptide expressing metaplasia of stomach.	Weis VG et al. Am J Physiol Gastr Liver Physiol 2014 [67]
CCT6A	1.52	2.1 × 10^−6^	Molecular chaperon. Member of the TCP1 ring complex.	Nd
RFC3	1.79	2.2 × 10^−6^	Replication factor. Its downregulation associates with MSI in GC.	Kim HR et al. Hum Pathol 2010 [68]
PRMT3	1.47	2.7 × 10^−6^	Arginine methyltransferases. Modifying enzyme of histones.	Nd
NELFCD	1.61	3.5 × 10^−6^	Member of the negative elongation factor complex.	Nd
ATAD2	1.69	4.8 × 10^−6^	ATPase, oncogene overexpressed in GC.	Zhang M et al. Clin Transl Oncol 2016 [69]
IDI1	1.56	5.2 × 10^−6^	Enzyme involved in the synthesis of farnesyl diphosphate and cholesterol.	Nd
EFNA1	1.75	5.7 × 10^−6^	Ligand for Eph receptors. Negative regulator in tumorigenesis via EPHA2 and FAK in GC.	Lee PC et al. Oncogene 2020 [70]
CSE1L	1.54	5.8 × 10^−6^	Mediator of cellular proliferation, invasion, and apoptosis via upregulation of MTIF activity in GC.	Li Y et al. J Cell Physiol 2020 [71]
TPX2	1.82	6.0 × 10^−6^	Spindle assembly factor upregulated in GC.	Huang J et al. Biomark Med 2020 [72]
GPR89B	1.45	6.3 × 10^−6^	Voltage dependent anion channel required for acidification and functions of the Golgi apparatus.	Nd
NUF2	1.72	7.1 × 10^−6^	Component of the essential kinetochore-associated NDC80 complex.	Nd
UBE2C	2.00	7.5 × 10^−6^	Marker of microsatellite instability in the intestinal GC type.	Wang R et al. Int J Oncol 2017 [73]
CDK1	1.64	1.0 × 10^−5^	Contributes to colony-forming ability of gastric cancer cells.	Li F et al. Int J Biol Sci 2021 [74]
DDX27	1.52	1.1 × 10^−5^	Contributes to colony-forming ability of gastric cancer cells.	Jin Y et al. Front Genet 2022 [75]
TMEM14A	1.45	1.3 × 10^−5^	Expression upregulated and correlated to worse prognosis in ovarian cancer.	Zhang X et al. Front Cell Dev Biol 2020 [76]
PDRG1	1.49	1.4 × 10^−5^	p53 and DNA damage-regulated gene upregulated in GC.	Zhang Y-J et al. Pathol Res Pract 2019 [77]
HIST1H2BD	2.08	1.6 × 10^−5^	Core component of nucleosome.	Nd
MSMO1	1.64	1.8 × 10^−5^	Methylsterol Monooxygenase 1. Enzyme of the cholesterol biosynthesis.	Nd
ETV4	2.33	1.9 × 10^−5^	ETV4 promotes the progression of gastric cancer through regulating KDM5D.	Cai L-S et al. Eur Rev Med Pharmacol Sci 2020 [78]
AURKA	1.89	1.9 × 10^−5^	Mitotic serine/threonine kinase overexpressed in GC	Orenay-Boyacioglu S et al. Mol Biol Rep 2018 [79]
MTBP	1.64	2.0 × 10^−5^	MDM2-binding protein downregulated in GC.	Wang W et al. Oncol Lett 2017 [80]
MRGBP	1.56	2.1 × 10^−5^	Component of the NuA4 histone acetyltransferase complex	Nd
GGH	2.5	2.2 × 10^−5^	Gamma-Glutamyl Hydrolase enzyme.	Nd
TDRKH	1.67	2.4 × 10^−5^	Catalyze the final steps of primary piRNA biogenesis	Nd
HIST1H3H	2.5	2.5 × 10^−5^	Core component of nucleosome.	Nd
RPN2	1.45	2.9 × 10^−5^	Subunit of the oligosaccharyl transferase complex. Multidrug resistance associated factor in GC.	Zhang H et al. Int J Biol Macromol 2019 [81]
RAB5IF	1.45	3.0 × 10^−5^	Assembly factor for mitochondrial respiratory complexes.	Nd
RAD54B	1.61	3.2 × 10^−5^	DNA repair and risk factor in GC.	Li W-Q et al. Carcinogenesis 2013 [82]
GAD1	4.17	3.5 × 10^−5^	Glutamic acid decarboxylase involved in GC.	Song Y et al. Histopathology 2013
POLE2	1.54	3.6 × 10^−5^	DNA Polymerase Epsilon 2.	Nd [83]
KRT80	3.12	4.3 × 10^−5^	Activated by lncRNA CircPIP5K1A in GC.	Song H et al. Biomed Pharmacother 2020 [84]
CPNE1	1.56	4.5 × 10^−5^	Calcium-dependent phospholipid-binding protein.	Nd
ACAT2	1.47	4.6 × 10^−5^	Cytosolic acetoacetyl-CoA thiolase.	Nd
DCAF13	1.47	5.2 × 10^−5^	Ribosomal RNA processing.	Nd
PRELID3B	1.52	5.6 × 10^−5^	PRELI Domain Containing 3B.	Nd
CMTM8	1.61	5.7 × 10^−5^	Chemokine-like factor downregulated in solid tumors	Nd
KIF14	1.56	5.9 × 10^−5^	Promotes tumor progression in GC.	Yang Z et al. BBA Mol Basis Dis 2019 [85]
CKS1B	1.49	6.8 × 10^−5^	Cyclin dependent kinases regulator involved in intestinal GC	Zhang J et al. Front Pharmacol 2018 [86]
CHKA	1.61	7.4 × 10^−5^	Choline Kinase Alpha	Nd

* FC, fold change; Nd, unknown.

**Table 3:** Listing of top 50 genes for which their mutated or wt status associates with a significantly different CDH1 level in GC.

Genes	FC *	*p*-Value	Function in GC or in General	Reference *
Upregulated				
PCDHA7	1.57	2.2 × 10^−5^	Protocadherin Alpha 7, neuronal.	Nd
CD93	1.49	6.4 × 10^−5^	Receptor for C1q. Mannose-binding protein.	Nd
XAB2	1.61	8.7 × 10^−5^	SNPs are associated with GC.	Xie Y et al. Int J Res Publ Health 2021 [87]
SGSM1	1.52	1.6 × 10^−4^	Small G Protein Modulator of RAB family	Nd
TRPC7	1.52	4.0 × 10^−4^	Transient Receptor Potential Cation Channel Subfamily C Member 7	Nd
BRINP3	1.47	5.1 × 10^−4^	Hypermethylated and downregulated in pre-GC and GC.	Chen L et al. Dis Markers 2012 [88]
ADAMTSL4	1.57	6.1 × 10^−4^	Disintegrin and metalloproteinase with thrombospondin motifs. Positive regulation of apoptosis.	Nd
STAMBPL1	1.59	1.0 × 10^−3^	STAMBPL1 knockdown inhibits GC.	Yu D-J et al. Oncol Lett 2019 [89]
TAOK1	1.67	1.5 × 10^−3^	Kinase involved in p38/MAPK14 stress-activated MAPK cascade. Negative regulator of inflammation.	Nd
TADA2B	1.61	1.7 × 10^−3^	Coactivates PAX5-dependent transcription together with either SMARCA4 or GCN5L2	Nd
PABPC3	1.5	1.8 × 10^−3^	Binds the poly[A] tail of mRNA.	Nd
ATP8B1	1.46	1.8 × 10^−3^	Catalytic component of a P4-ATPase flippase complex.	Nd
ADAM22	1.49	2.0 × 10^−3^	Non-catalytic metalloprotease-like protein, neuronal. Regulator of cell adhesion, spreading, inhibition of cell proliferation	Nd
POU5F2	1.71	2.0 × 10^−3^	Transcription factor required for terminal differentiation of male germ cell.	Nd
P3H1	1.59	2.1 × 10^−3^	Basement membrane-associated chondroitin sulfate proteoglycan with prolyl 3-hydroxylase activity.	Nd
SMARCA4	1.45	2.1 × 10^−3^	Loss of chromatin remodeling SMARCA4 associated to GC subtypes.	Huang S-C et al. Histopathology 2020 [90]
KCNV2	1.48	2.2 × 10^−3^	Voltage-gated potassium [Kv] channels subunit.	Nd
FAM193A	1.44	2.3 × 10^−3^	Uncharacterized protein.	Nd
MOCOS	1.53	2.4 × 10^−3^	Sulfurates the molybdenum cofactor.	Nd
UBA6	1.77	2.4 × 10^−3^	Required for UBD/FAT10 conjugation.	Nd
KIAA1683	1.47	2.5 × 10^−3^	Alias of IQCN. Uncharacterized protein.	Nd
TROAP	1.48	2.7 × 10^−3^	Cell adhesion. Its downregulation inhibits cell proliferation of GC.	Jing K et al. Mol Med Rep 2018 [91]
CDH16	1.45	2.7 × 10^−3^	Cadherin 16, associated to renal oncocytoma.	Nd
DHX35	1.6	2.7 × 10^−3^	DEAD box proteins, putative RNA helicases.	Nd
SMC3	1.57	2.7 × 10^−3^	Cohesin, required for cell cycle.	Nd
GAD1	1.5	2.8 × 10^−3^	Glutamic acid decarboxylase involved in GC	Song Y et al. Histopathology 2013 [83]
MAP3K6	1.46	3.0 × 10^−3^	Germline mutations are associated with familial GC.	Gaston D et al. PloS Genet 2014 [92]
ZNF418	1.61	3.1 × 10^−3^	Its overexpression is protective to GC.	Hui H-X et al. Onco Target Ther 2018 [93]
RBM22	1.80	3.1 × 10^−3^	Component of spliceosome.	Nd
FMNL2	1.62	3.2 × 10^−3^	Formin member, morphogenesis. FMNL2 silencing inhibits GC.	Zhong B et al. Cancer Cell Int 2018 [94]
IFFO1	1.73	3.3 × 10^−3^	Nuclear matrix intermediate filament.	Nd
KIAA0556	1.49	3.3 × 10^−3^	Alias of KATNIP. Katanin Interacting ciliary protein.	Nd
MAP4K4	1.47	3.4 × 10^−3^	MAP kinase upstream to JUN pathway. Its silencing suppresses GC proliferation.	Liu YF et al. Mol Med Rep 2016 [95]
NETO2	1.47	3.7 × 10^−3^	Subunit of neuronal ainite-sensitive glutamate. Promotes GC invasion.	Liu JY et al. Cell Death Dis 2019 [96]
TBPL2	1.56	3.7 × 10^−3^	Transcription factor required for myocytes differentiation.	Nd
DIAPH1	1.5	3.8 × 10^−3^	Formin-related, cell morphology, and cytoskeletal.	Nd
LPCAT3	1.68	3.8 × 10^−3^	Lysophospholipid O-acyltransferase.	Nd
ELP3	1.56	3.8 × 10^−3^	Catalytic subunit of the histone acetyltransferase elongator complex.	Nd
ZFP57	1.52	3.9 × 10^−3^	Zinc finger protein required to maintain maternal and paternal gene imprinting.	Nd
FGF21	2.14	4.0 × 10^−3^	Fibroblast growth factor. Serum marker of GC.	Kimak E et al. Pol Arch Intern Med 2019 [97]
ZMPSTE24	1.62	4.0 × 10^−3^	Zinc metalloproteinase in lamin A pathway. Involved in MSI-H GC.	Lee JH et al. Pathol Res Pract 2016 [98]
EEF1D	1.60	4.2 × 10^−3^	Subunit of the elongation factor 1 complex.	Nd
GABBR2	1.45	4.2 × 10^−3^	Component of a heterodimeric G-protein coupled receptor for GABA.	Nd
SGCE	1.60	4.2 × 10^−3^	Component of the sarcoglycan complex. Predicts prognosis of GC.	Hou J-Y et al. J Cancer Res Clin Oncol 2017 [99]
SMARCB1	1.53	4.2 × 10^−3^	Chromatin remodeling protein involved in GC.	Mochizuki K et al. Anticancer Res 2018 [100]
NR0B2	2.23	4.3 × 10^−3^	Inhibitor of transactivation of nuclear receptors.	Nd
ATN1	1.47	4.4 × 10^−3^	Transcriptional corepressor. Recruits NR2E1 to repress transcription.	Nd
HPR	1.82	4.4 × 10^−3^	Haptoglobin-related protein.	Nd
ECT2L	1.47	4.5 × 10^−3^	Guanine nucleotide exchange factor.	Nd
CYP2U1	1.56	4.6 × 10^−3^	Cytochrome P450 monooxygenase, metabolism of arachidonic acid.	Nd
ITPRID2	1.44	4.6 × 10^−3^	Unknown	Nd
Downregulated				
CDH1	1.49	1.2 × 10^−4^	Cadherine 1, calcium-dependent cell–cell adhesion glycoprotein	Guilford P et al. Nature 1998 [101]

* FC, fold change; Nd, unknown.

**Table 4:** Listing of the 50 gene sets enriched with genes whose expression is positively correlated to CDH1 in GC.

Library	Gene Set	LogFC *	*p*. Value	Adj *p*. Value *
TRRUST_Transcription_Factors_2019	SNAI2 mouse	0.14	1.58 × 10^−24^	6.83 × 10^−21^
ARCHS4_TFs_Coexp	EHF human tf ARCHS4 coexpression	0.14	1.60 × 10^−24^	6.83 × 10^−21^
ARCHS4_TFs_Coexp	TFCP2L1 human tf ARCHS4 coexpression	0.12	1.14 × 10^−23^	2.92 × 10^−20^
ARCHS4_TFs_Coexp	OVOL2 human tf ARCHS4 coexpression	0.11	1.37 × 10^−23^	2.92 × 10^−20^
RNAseq_Automatic_GEO_Signatures_Mouse_Down	Ago2 Sufficent Pancreatic Lsl GSE147781 1	0.13	4.94 × 10^−24^	4.20 × 10^−20^
ARCHS4_TFs_Coexp	GRHL2 human tf ARCHS4 coexpression	0.13	7.55 × 10^−23^	1.29 × 10^−19^
RNAseq_Automatic_GEO_Signatures_Human_Down	Ogt Knockout S2Vp10 Cells GSE114472 1	0.11	1.16 × 10^−22^	3.69 × 10^−19^
RNAseq_Automatic_GEO_Signatures_Mouse_Down	Kras Rank Rankfl Pneumocytes GSE81670 1	0.10	1.30 × 10^−22^	3.69 × 10^−19^
ARCHS4_Kinases_Coexp	ERBB3 human kinase ARCHS4 coexpression	0.14	9.90 × 10^−23^	3.82 × 10^−19^
ARCHS4_TFs_Coexp	KLF5 human tf ARCHS4 coexpression	0.14	2.97 × 10^−22^	4.02 × 10^−19^
ARCHS4_TFs_Coexp	ELF3 human tf ARCHS4 coexpression	0.12	3.30 × 10^−22^	4.02 × 10^−19^
CellMarker_Augmented_2021	Flk1 + Mesodermal cell:Pluripotent Stem Cell	0.11	2.84 × 10^−21^	1.09 × 10^−18^
ARCHS4_IDG_Coexp	FXYD3 IDG ionchannel ARCHS4 coexpression	0.13	4.25 × 10^−22^	4.44 × 10^−18^
TRRUST_Transcription_Factors_2019	ZEB1 human	0.13	4.93 × 10^−21^	4.89 × 10^−18^
ARCHS4_TFs_Coexp	MST1R human tf ARCHS4 coexpression	0.15	5.16 × 10^−21^	4.89 × 10^−18^
ARCHS4_Kinases_Coexp	STYK1 human kinase ARCHS4 coexpression	0.13	2.02 × 10^−21^	5.84 × 10^−18^
Reactome_2016	Signaling by MST1 Homo sapiens R-I−8852405	0.17	3.20 × 10^−21^	7.41 × 10^−18^
ENCODE_TF_ChIP-seq_2015	ZEB1 HepG2 hg19	0.10	9.58 × 10^−21^	8.19 × 10^−18^
ARCHS4_Kinases_Coexp	MST1R human kinase ARCHS4 coexpression	0.15	5.45 × 10^−21^	1.05 × 10^−17^
RNAseq_Automatic_GEO_Signatures_Human_Up	Runx Transciption Factor Mediated GSE141503 1	0.09	2.78 × 10^−20^	2.81 × 10^−17^
RNAseq_Automatic_GEO_Signatures_Human_Down	Re-Induction Baruch Science 2020 GSE162436 2	0.13	2.99 × 10^−20^	2.81 × 10^−17^
ARCHS4_TFs_Coexp	OVOL1 human tf ARCHS4 coexpression	0.12	4.05 × 10^−20^	3.15 × 10^−17^
ARCHS4_IDG_Coexp	TMC4 IDG ionchannel ARCHS4 coexpression	0.11	1.30 × 10^−20^	4.53 × 10^−17^
RNAseq_Automatic_GEO_Signatures_Mouse_Up	Elf5 C3-T Elf5-Gfp Reporter GSE122180 1	0.10	9.19 × 10^−20^	7.42 × 10^−17^
InterPro_Domains_2019	BRO1 domain	0.15	7.38 × 10^−20^	9.82 × 10^−17^
Pfam_Domains_2019	BRO1	0.15	7.38 × 10^−20^	9.82 × 10^−17^
InterPro_Domains_2019	Growth factor receptor domain 4	0.14	7.42 × 10^−20^	9.82 × 10^−17^
Pfam_Domains_2019	GF recep IV	0.14	7.42 × 10^−20^	9.82 × 10^−17^
BioPlex_2017	APOB	0.12	6.71 × 10^−20^	1.11 × 10^−16^
GTEx_Tissue_Sample_Gene_Expression_Profiles_up	GTEX-SNMC−0626-SM−4DM6H stomach male 20–29 years	0.11	1.05 × 10^−18^	1.12 × 10^−16^
ARCHS4_Kinases_Coexp	PTK6 human kinase ARCHS4 coexpression	0.11	9.25 × 10^−20^	1.34 × 10^−16^
RNAseq_Automatic_GEO_Signatures_Mouse_Down	Kras Hdac5 Eccaper Pdac GSE149126 1	0.10	2.26 × 10^−19^	1.59 × 10^−16^
InterPro_Domains_2019	MANSC domain	0.14	1.87 × 10^−19^	2.06 × 10^−16^
ARCHS4_TFs_Coexp	ZBTB7C human tf ARCHS4 coexpression	0.10	3.88 × 10^−19^	2.75 × 10^−16^
ARCHS4_TFs_Coexp	LGR4 human tf ARCHS4 coexpression	0.10	4.19 × 10^−19^	2.75 × 10^−16^
RNAseq_Automatic_GEO_Signatures_Human_Up	Overexpression Proliferation Drivers Mammary GSE109326 4	0.11	8.36 × 10^−19^	4.89 × 10^−16^
ENCODE_and_ChEA_Consensus_TFs_from_ChIP-X	ZEB1 ENCODE	0.10	1.18 × 10^−18^	7.19 × 10^−16^
Pfam_InterPro_Domains	Aminotrans I/II	0.11	8.89 × 10^−19^	8.41 × 10^−16^
ARCHS4_IDG_Coexp	SCNN1B IDG ionchannel ARCHS4 coexpression	0.10	4.10 × 10^−19^	1.07 × 10^−15^
BioPlex_2017	ITM2A	0.12	8.80 × 10^−19^	1.13 × 10^−15^
Drug_Perturbations_from_GEO_2014	vitamin c mus musculus gpl6246 gse19378 chdir up	0.07	9.42 × 10^−19^	3.20 × 10^−15^

* log FC: log2 of fold change enrichment; Adj *p*. Value, adjusted *p*-value.

**Table 5:** Protein-coding gene variants differentially expressed in GC cases with altered CDH1 compared to normal stomach tissue.

Gene Symbol-Isoform	Gene ID ENST	Tumor mRNA *	Normal mRNA *	Adjusted *p*-Value	Characteristics [aa Length vs. Refseq]
From Table 1					
ASPN−002	00000375543	1.2	0.01	2.95 × 10^−30^	Unique 243 aa vs. 380 aa
CCDC80-006	00000479368	0.001	1.57	5.27 × 10^−35^	Unique 212 aa vs. 950 aa
CCDC80-007	00000461431	0.15	1.70	1.13 × 10^−33^	Unique 261 aa vs. 950 aa
COL16A1-013	00000458715	1.43	4.66	5.04 × 10^−22^	Unique 235 aa vs. 1604 aa
COL6A3-012	00000472056	60.97	11.93	6.61 × 10^−22^	Unique 2570 aa vs. 3177 aa
COL6A3-202	00000353578	9.02	0.38	3.61 × 10^−66^	Unique 2971 aa vs. 3177 aa
FBLN2-002	00000295760	8.85	2.59	1.70 × 10^−14^	Unique 1184 aa vs. 1231 aa
PRRX1-001	00000367760	1.53	0.12	5.67 × 10^−33^	Unique 217 aa vs. 245 aa
From Table 2					
AURKA-007	00000456249	1.08	0.01	5.44 × 10^−37^	Unique 106 aa vs. 403aa
GGH-003	00000518113	1.72	0.22	3.10 × 10^−30^	Unique 271 aa vs. 318 aa
PRMT3-002	00000330796	2.62	0.64	5.90 × 10^−36^	Shared 46 aa vs. 531aa
RAE1-009	00000527947	9.34	3.17	8.08 × 10^−29^	Unique 437 aa vs. 368 aa
UBE2C-003	00000335046	1.02	0,01	8.98 × 10^−48^	Unique 161 aa vs. 179 aa
UBE2C-004	00000372568	3.84	0.15	5.29 × 10^−64^	Unique 140 aa vs. 179 aa
UBE2C-006	00000352551	3.13	0.01	8.42 × 10^−56^	Unique 150 aa vs. 179 aa
From Table 3					
ANXA4-002	00000409920	33.15	9.56	1.7 × 10^−17^	Unique 299 aa vs. 321 aa
ARHGEF28-006	00000512883	0.46	3.34	5.4 × 10^−31^	Unique 651 aa vs. 1731 aa
DSC2-002	00000251081	10.07	2.07	1.31 × 10^−13^	Unique 847 aa vs. 901 aa
EEF1D-019	00000529516	9.92	-3.44	1.64 × 10^−93^	Shared 262 aa vs. 647 aa
EEF1D-045	00000533494	0.49	1.48	1.14 × 10^−36^	Shared 281aa vs. 647 aa
ELP3-003	00000524103	1.46	0.01	6.09 × 10^−20^	Unique 475 aa vs. 547 aa
ELP3-004	00000518112	2.95	0.37	1.13 × 10^−40^	Shared 45 aa vs. 547 aa
EPB41L1-003	00000427533	2.28	0.32	2.43 × 10^−36^	Unique 59 aa vs. 1595 aa
EPB41L1-012	00000432603	12.10	2.68	2.68 × 10^−49^	Unique 119 aa vs. 1595 aa
EPB41L1-015	00000406771	8.52	2.01	1.65 × 10^−33^	Unique 168 aa vs. 1595 aa
EPB41L1-016	00000628415	11.35	4.96	5.9 × 10^−26^	Unique 701 aa vs. 1595 aa
FBXO38-201	00000296701	1.14	0.01	3.8 × 10^−13^	Shared 943 aa vs. 1188 aa
KIF18B-002	00000587309	5.02	0.24	2.05 × 10^−77^	Unique 833 aa vs. 852 aa
MAP4K4-001	00000347699	5.25	0.01	4.46 × 10^−44^	Unique 1239 aa vs. 1273 aa
MUC20-001	00000447234	4.96	0.15	1.07 × 10^−29^	Unique 709 aa vs. 723 aa
MUC20-201	00000320736	18.33	3.75	3.86 × 10^−14^	Unique 538 aa vs. 723 aa
P3H1-001	00000296388	11.09	2.31	1.56 × 10^−74^	Unique 736 aa vs. 804 aa
P3H1-201	00000397054	1.49	0.19	1.72 × 10^−59^	Unique 697 aa vs. 804 aa
SGCE-002	00000437425	0.62	2.31	6.66 × 10^−20^	Unique 396 aa vs. 473 aa
SMARCA4-003	00000444061	9.89	2.68	2.24 × 10^−56^	Shared 1613 aa vs. 1647 aa
TMEM132A-010	00000544065	1.6	0.01	6.88 × 10^−27^	Unique 76 aa vs. 1023 aa
TROAP-001	00000257909	4.46	0.25	1.41 × 10^−79^	Unique 778 aa vs. 868
TROAP-002	00000380327	1.06	0.01	2.29 × 10^−33^	Unique 144 aa vs. 868
TROAP-005	00000548311	2.96	0.16	4.11 × 10^−52^	Shared 126 aa vs. 868 aa
TROAP-009	00000550709	1.19	0.09	4.87 × 10^−31^	Shared 126 aa vs. 868 aa
UBA6-005	00000505673	2.35	0.26	9.77 × 10^−23^	Unique 271 aa vs. 1052 aa
USP22-003	00000476111	16.16	0.01	1.53 × 10^−42^	Unique 125 aa vs. 525 aa
USP22-005	00000537526	0.06	18.48	3.73 × 10^−66^	Unique 513 aa vs. 525 aa

* mRNA level (Transcripts Per Million) was obtained from http://gepia2.cancer-pku.cn (accessed on 8 February 2022); ENSTXXXXXXXXXXX is the Gene ID (accessed on 1 July 2021), according to www.ensemble.org; aa, amino acids; unique, a mRNA isoform which codes for a protein with nonrecurring aa length; shared, a mRNA isoform that codes for a protein with recurring aa length.

## Data Availability

Data represented or analyzed in this study are openly available as such or can be obtained by means of the software: Stomach Adenocarcinoma, Firehose Legacy Study at https://gdac.broadinstitute.org/runs/stddata__2016_01_28/data/STAD/20160128/ (accessed on 20 October 2021) and https://www.cbioportal.org/study/summary?id=stad_tcga (accessed on 30 December 2021); MuTarget at https://www.mutarget.com (accessed on 13 August 2022); STRING at https://string-db.org/cgi/input?sessionId=bB2BdwaOF2oJ&input_page_show_search=off (accessed on 10 October 2021); GEPIA2 at http://gepia2.cancer-pku.cn/#index (accessed on 8 February 2022); KMPLOT at https://kmplot.com/analysis/index.php?*p*=service&cancer=pancancer_rnaseq (accessed on 6 May 2022); Enrichr at https://maayanlab.cloud/Enrichr/ (accessed on 1 December 2021); L1000CDS2 at https://maayanlab.cloud/L1000CDS2/#/index (accessed on 1 September 2022).

## Supplementary Materials

The following supporting information can be downloaded at: https://www.mdpi.com/article/10.3390/jpm12122006/s1, Figure S1: A–O. Gene enrichment analysis on genes correlated with CDH1 mRNA level in 483 GC cases from cBioPortal, Stomach Adenocarcinoma, Firehose Legacy Study. The analysis was performed as follows: a total of 124 gene set collections summing up to 147,592 gene sets were obtained from Enrichr at https://maayanlab.cloud/Enrichr/ (29). The collections were divided into 13 functionally related groups (blocks A–O) and used to perform gene set variation analysis (GSVA) (30). Subsequently, differential enrichment analysis on gene correlation with CDH1 gene expression were performed using limma software tool package for each block (31); Figure S2: Representation of genomic data referred to 47 genes downregulated in CDH1-mutated compared to CDH1-wt GC cases. Genomic data of 47 of the 50 genes reported in Table 2, plus CDH1, in 415 GC cases divided into 6 subtypes (from cBioPortal, Stomach Adenocarcinoma, Firehose Legacy study). Genetic data of genes LINC00511, GPR89B and CKS1B were not available. Red-blu color scale indicates variation from higher to lower than diploid samples mRNA level (z-scores, RNA Seq V2 RSEM): light red, upregulated; light blu downregulated. Dark red, gained compared to diploid; dark blu, lost compared to diploid. Grey, invariant; Figure S3: A–C. Protein interactomes of CDH1-associated transcriptional patterns. Interactomes of proteins (Table 1) upregulated in GC-mutated versus non-mutated GC cases (A), proteins (Table 2) down-regulated in GC-mutated versus non-mutated cases (B), proteins (Table 3) associated with CDH1 upregulation in GC-mutated versus non-mutated GC cases (C). Conditions applied using the software tool STRING: physical interactions; interaction score medium confidence; MCL clustering, inflation 3. Not connected nodes were removed. Clusters are identified by different colors. CDH1 is identified by a rectangle; Figure S4: CDH1 mRNA levels and copy number status in 483 GC cases. The graph is reproduced from cBioPortal, Stomach Adenocarcinoma, Firehose Legacy Study; Figure S5: CDH1 transcript levels as a function of gene methylation in 483 GC cases. The graph is reproduced from cBioPortal, Stomach Adenocarcinoma, Firehose Legacy Study; Table S1: Signaling pathways associated with upregulated and downregulated genes in CDH1-mutated versus non-mutated DGC (diffuse GC type) according to the MSigDB database; Table S2: Listing of small drug compounds targeting the top 20 genes found to be up- or down-regulated in CDH1-mutated versus non-mutated GCs, including DGC (diffuse GC type); Table S3: Listing of best combinations of small drug compounds targeting the genes found to be up- or down-regulated in CDH1-mutated versus non-mutated GCs and DGC (diffuse GC type).
