# Supplementary material for: Landscape of Druggable Molecular Pathways Downstream of Genomic CDH1/Cadherin-1 Alterations in Gastric Cancer
Source: J Pers Med. 2022 Dec 3;12(12):2006. doi: 10.3390/jpm12122006 (PMC9784514; doi:10.3390/jpm12122006)
Supplement: Supplementary file 1 [file jpm-12-02006-s001.zip › jpm-1947899-supplementary.pdf]

Supplementary Figures and Tables

Landscape of Druggable Molecular Pathways Downstream of Genomic CDH1/Cadherin-1 Alterations in Gastric Cancer

Giorgio Malpeli[1], Stefano Barbi[2], Giulio Innamorati[1], Mariella Alloggio[3], Federica Filippini[3], Ilaria Decimo[4], Claudia Castelli[5], Roberto Perris[6], Maria Bencivenga[3]

[1]Department of Surgical, Odontostomatologic, Maternal and Child Sciences, University of Verona, 37134, Verona, Italy
[2]Department of Diagnostics and Public Health, University and Hospital Trust of Verona, 37134 Verona, Italy
[3]General and Upper GI Surgery Division, Department and of Surgical, Odontostomatologic, Maternal and Child Sciences, University of Verona, 37134 Verona, Italy
[4]Section of Pharmacology, Department of Diagnostic and Public Health, University of Verona, 37134 Verona, Italy.
[5]Pathology Unit, Department of Diagnostics and Public Health, University and Hospital Trust of Verona, 37134 Verona, Italy
[6]Department of Biosciences, COMT-Centre for Molecular and Translational Oncology, University of Parma, 43124 Parma, Italy

A

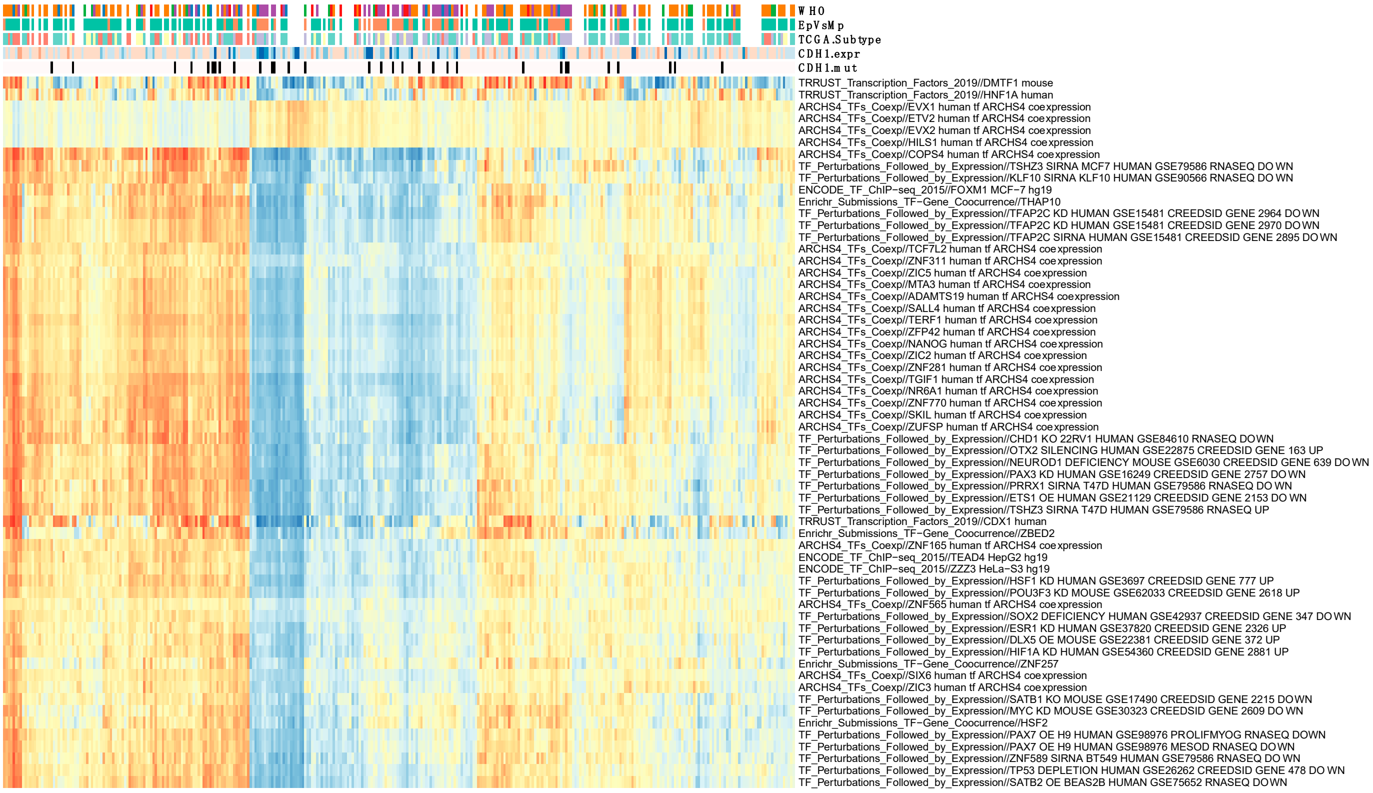

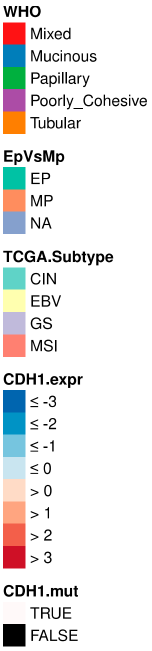
WHO
Mixed
Mucinous
Papillary
Poorly_Cohesive
Tubular
EpVsMp
EP
MP
NA
TCGA.Subtype
CIN
EBV
GS
MSI
CDH1.expr
≤ -3
≤ -2
≤ -1
≤ 0
> 0
> 1
> 2
> 3
CDH1.mut
TRUE
FALSE

B

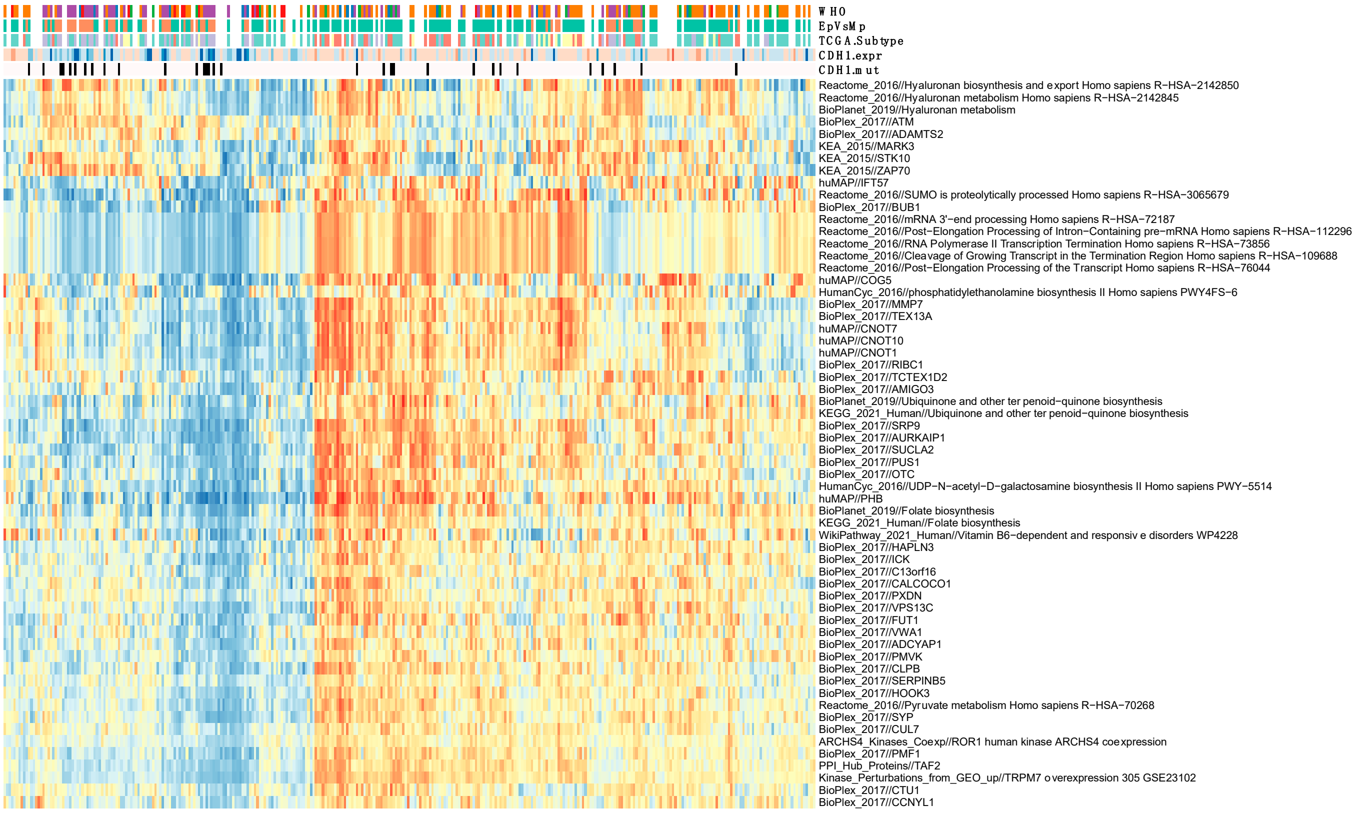
WHO
EpVsMp
TCGA.Subtype
CDH1.expr
CDH1.mut
Reactome_2016//Hyaluronan biosynthesis and export Homo sapiens R-HSA-2142850
Reactome_2016//Hyaluronan metabolism Homo sapiens R-HSA-2142845
BioPlanet_2019//Hyaluronan metabolism
BioPlex_2017//ATM
BioPlex_2017//ADAMTS2
KEA_2015//MARK3
KEA_2015//STK10
KEA_2015//ZAP70
huMAP//IFT57
Reactome_2016//SUMO is proteolytically processed Homo sapiens R-HSA-3065679
BioPlex_2017//BUB1
Reactome_2016//mRNA 3'-end processing Homo sapiens R-HSA-72187
Reactome_2016//Post-Elongation Processing of Intron-Containing pre-mRNA Homo sapiens R-HSA-112296
Reactome_2016//RNA Polymerase II Transcription Termination Homo sapiens R-HSA-73856
Reactome_2016//Cleavage of Growing Transcript in the Termination Region Homo sapiens R-HSA-109688
Reactome_2016//Post-Elongation Processing of the Transcript Homo sapiens R-HSA-76044
huMAP//COG5
HumanCyc_2016//phosphatidylethanolamine biosynthesis II Homo sapiens PWY4FS-6
BioPlex_2017//MMP7
BioPlex_2017//TEX13A
huMAP//CNOT7
huMAP//CNOT10
huMAP//CNOT1
BioPlex_2017//RIBC1
BioPlex_2017//TCTEX1D2
BioPlex_2017//AMIGO3
BioPlanet_2019//Ubiquinone and other terpenoid-quinone biosynthesis
KEGG_2021_Human//Ubiquinone and other terpenoid-quinone biosynthesis
BioPlex_2017//SRP9
BioPlex_2017//AURKAIP1
BioPlex_2017//SUCLA2
BioPlex_2017//PUS1
BioPlex_2017//OTC
HumanCyc_2016//UDP-N-acetyl-D-galactosamine biosynthesis II Homo sapiens PWY-5514
huMAP//PHB
BioPlanet_2019//Folate biosynthesis
KEGG_2021_Human//Folate biosynthesis
WikiPathway_2021_Human//Vitamin B6-dependent and responsive disorders WP4228
BioPlex_2017//HAPLN3
BioPlex_2017//ICK
BioPlex_2017//C13orf16
BioPlex_2017//CALCOCO1
BioPlex_2017//PXDN
BioPlex_2017//VPS13C
BioPlex_2017//FUT1
BioPlex_2017//VWA1
BioPlex_2017//ADCYAP1
BioPlex_2017//PMVK
BioPlex_2017//CLPB
BioPlex_2017//SERPINB5
BioPlex_2017//HOOK3
Reactome_2016//Pyruvate metabolism Homo sapiens R-HSA-70268
BioPlex_2017//SYP
BioPlex_2017//CUL7
ARCHS4_Kinases_Coexp//ROR1 human kinase ARCHS4 coexpression
BioPlex_2017//PMF1
PPI_Hub_Proteins//TAF2
Kinase_Perturbations_from_GEO_up//TRPM7 overexpression 305 GSE23102
BioPlex_2017//CTU1
BioPlex_2017//CCNYL1

C

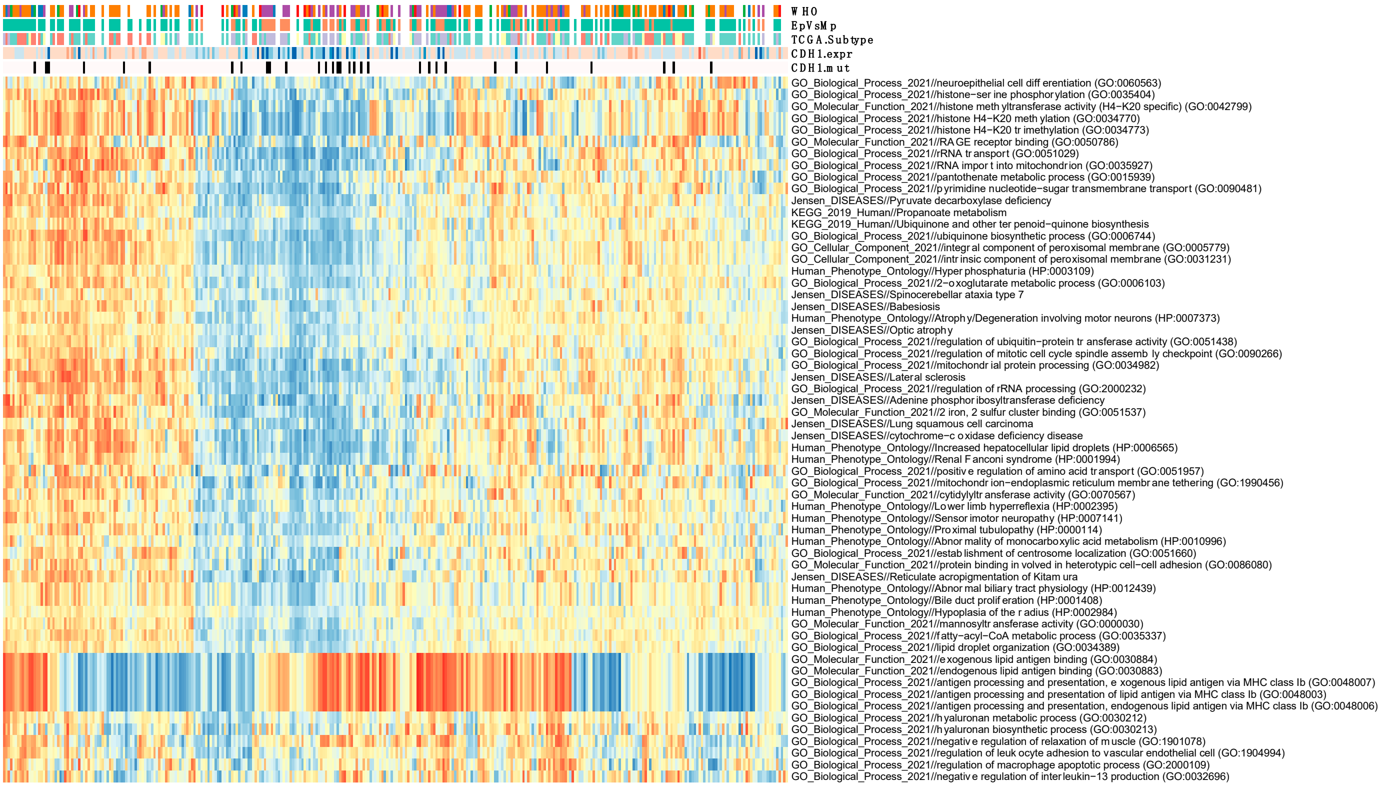
WHO
EpVsMp
TCGA.Subtype
CDH1.expr
CDH1.mut
GO_Biological_Process_2021//neuroepithelial cell differentiation (GO:0060563)
GO_Biological_Process_2021//histone-serine phosphorylation (GO:0035404)
GO_Molecular_Function_2021//histone methyltransferase activity (H4-K20 specific) (GO:0042799)
GO_Biological_Process_2021//histone H4-K20 methylation (GO:0034770)
GO_Biological_Process_2021//histone H4-K20 trimethylation (GO:0034773)
GO_Molecular_Function_2021//RAGE receptor binding (GO:0050786)
GO_Biological_Process_2021//rRNA transport (GO:0051029)
GO_Biological_Process_2021//RNA import into mitochondrion (GO:0035927)
GO_Biological_Process_2021//pantothenate metabolic process (GO:0015939)
GO_Biological_Process_2021//pyrimidine nucleotide-sugar transmembrane transport (GO:0090481)
Jensen_DISEASES//Pyruvate decarboxylase deficiency
KEGG_2019_Human//Propanoate metabolism
KEGG_2019_Human//Ubiquinone and other terpenoid-quinone biosynthesis
GO_Biological_Process_2021//ubiquinone biosynthetic process (GO:0006744)
GO_Cellular_Component_2021//integral component of peroxisomal membrane (GO:0005779)
GO_Cellular_Component_2021//intrinsic component of peroxisomal membrane (GO:0031231)
Human_Phenotype_Ontology//Hyperphosphaturia (HP:0003109)
GO_Biological_Process_2021//2-oxoglutarate metabolic process (GO:0006103)
Jensen_DISEASES//Spinocerebellar ataxia type 7
Jensen_DISEASES//Babesiosis
Human_Phenotype_Ontology//Atrophy/Degeneration involving motor neurons (HP:0007373)
Jensen_DISEASES//Optic atrophy
GO_Biological_Process_2021//regulation of ubiquitin-protein transferase activity (GO:0051438)
GO_Biological_Process_2021//regulation of mitotic cell cycle spindle assembly checkpoint (GO:0090266)
GO_Biological_Process_2021//mitochondrial protein processing (GO:0034982)
Jensen_DISEASES//Lateral sclerosis
GO_Biological_Process_2021//regulation of rRNA processing (GO:2000232)
Jensen_DISEASES//Adenine phosphoribosyltransferase deficiency
GO_Molecular_Function_2021//2 iron, 2 sulfur cluster binding (GO:0051537)
Jensen_DISEASES//Lung squamous cell carcinoma
Jensen_DISEASES//cytochrome-c oxidase deficiency disease
Human_Phenotype_Ontology//Increased hepatocellular lipid droplets (HP:0006565)
Human_Phenotype_Ontology//Renal Fanconi syndrome (HP:0001994)
GO_Biological_Process_2021//positive regulation of amino acid transport (GO:0051957)
GO_Biological_Process_2021//mitochondrion-endoplasmic reticulum membrane tethering (GO:1990456)
GO_Molecular_Function_2021//cytidylyltransferase activity (GO:0070567)
Human_Phenotype_Ontology//Lower limb hyperreflexia (HP:0002395)
Human_Phenotype_Ontology//Sensorimotor neuropathy (HP:0007141)
Human_Phenotype_Ontology//Proximal tubulopathy (HP:0000114)
Human_Phenotype_Ontology//Abnormality of monocarboxylic acid metabolism (HP:0010996)
GO_Biological_Process_2021//establishment of centrosome localization (GO:0051660)
GO_Molecular_Function_2021//protein binding involved in heterotypic cell-cell adhesion (GO:0086080)
Jensen_DISEASES//Reticulate acropigmentation of Kitamura
Human_Phenotype_Ontology//Abnormal biliary tract physiology (HP:0012439)
Human_Phenotype_Ontology//Bile duct proliferation (HP:0001408)
Human_Phenotype_Ontology//Hypoplasia of the radius (HP:0002984)
GO_Molecular_Function_2021//mannosyltransferase activity (GO:0000030)
GO_Biological_Process_2021//fatty-acyl-CoA metabolic process (GO:0035337)
GO_Biological_Process_2021//lipid droplet organization (GO:0034389)
GO_Molecular_Function_2021//exogenous lipid antigen binding (GO:0030884)
GO_Molecular_Function_2021//endogenous lipid antigen binding (GO:0030883)
GO_Biological_Process_2021//antigen processing and presentation, exogenous lipid antigen via MHC class Ib (GO:0048007)
GO_Biological_Process_2021//antigen processing and presentation of lipid antigen via MHC class Ib (GO:0048003)
GO_Biological_Process_2021//antigen processing and presentation, endogenous lipid antigen via MHC class Ib (GO:0048006)
GO_Biological_Process_2021//hyaluronan metabolic process (GO:0030212)
GO_Biological_Process_2021//hyaluronan biosynthetic process (GO:0030213)
GO_Biological_Process_2021//negative regulation of relaxation of muscle (GO:1901078)
GO_Biological_Process_2021//regulation of leukocyte adhesion to vascular endothelial cell (GO:1904994)
GO_Biological_Process_2021//regulation of macrophage apoptotic process (GO:2000109)
GO_Biological_Process_2021//negative regulation of interleukin-13 production (GO:0032696)

D

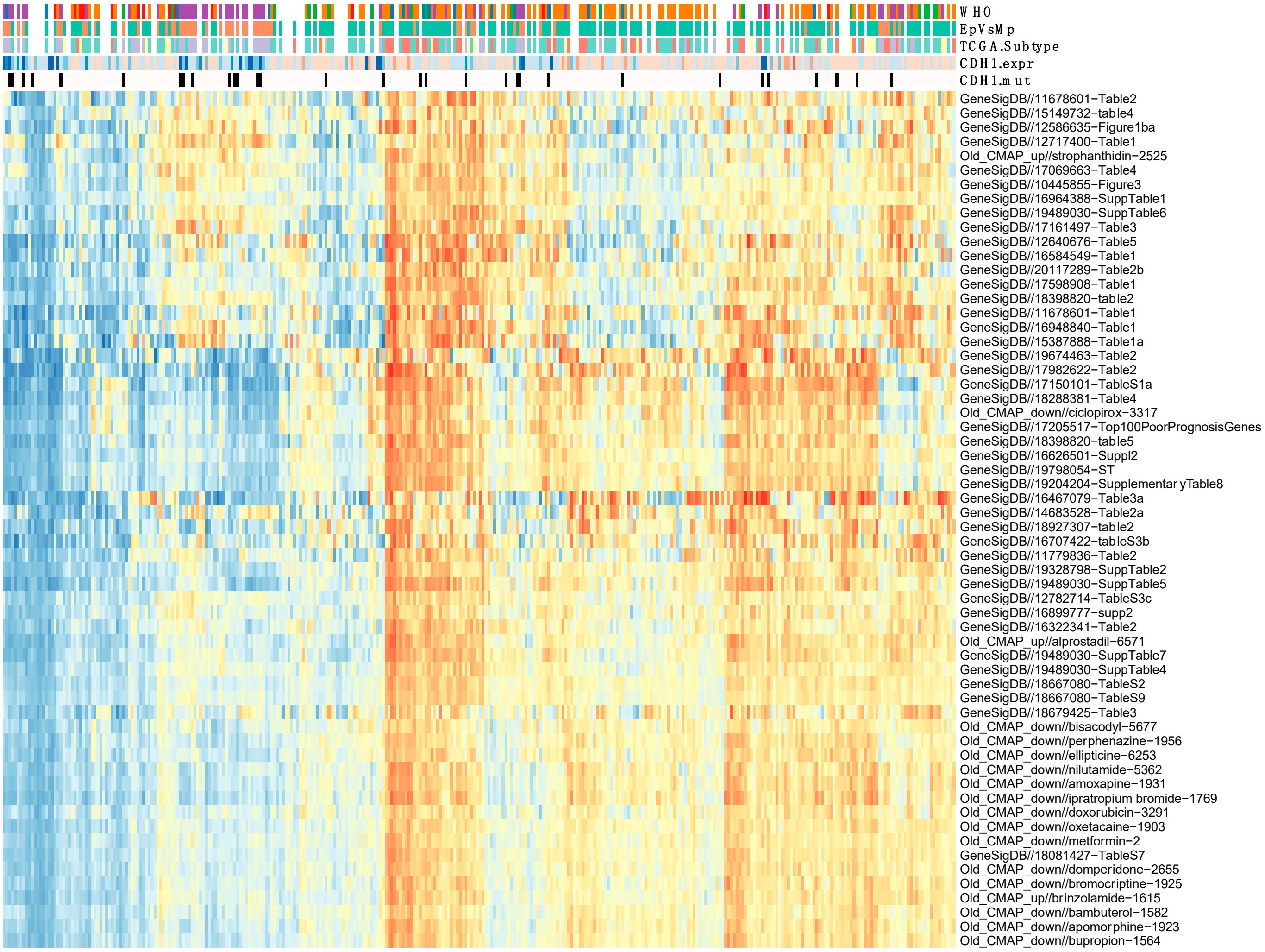
WHO
EpVsMp
TCGA.Subtype
CDH1.expr
CDH1.mut
GeneSigDB//11678601-Table2
GeneSigDB//15149732-table4
GeneSigDB//12586635-Figure1ba
GeneSigDB//12717400-Table1
Old_CMAP_up//strophanthidin-2525
GeneSigDB//17069663-Table4
GeneSigDB//10445855-Figure3
GeneSigDB//16964388-SuppTable1
GeneSigDB//19489030-SuppTable6
GeneSigDB//17161497-Table3
GeneSigDB//12640676-Table5
GeneSigDB//16584549-Table1
GeneSigDB//20117289-Table2b
GeneSigDB//17598908-Table1
GeneSigDB//18398820-table2
GeneSigDB//11678601-Table1
GeneSigDB//16948840-Table1
GeneSigDB//15387888-Table1a
GeneSigDB//19674463-Table2
GeneSigDB//17982622-Table2
GeneSigDB//17150101-TableS1a
GeneSigDB//18288381-Table4
Old_CMAP_down//ciclopirox-3317
GeneSigDB//17205517-Top100PoorPrognosisGenes
GeneSigDB//18398820-table5
GeneSigDB//16626501-Suppl2
GeneSigDB//19798054-ST
GeneSigDB//19204204-SupplementaryTable8
GeneSigDB//16467079-Table3a
GeneSigDB//14683528-Table2a
GeneSigDB//18927307-table2
GeneSigDB//16707422-tableS3b
GeneSigDB//11779836-Table2
GeneSigDB//19328798-SuppTable2
GeneSigDB//19489030-SuppTable5
GeneSigDB//12782714-TableS3c
GeneSigDB//16899777-supp2
GeneSigDB//16322341-Table2
Old_CMAP_up//alprostadil-6571
GeneSigDB//19489030-SuppTable7
GeneSigDB//19489030-SuppTable4
GeneSigDB//18667080-TableS2
GeneSigDB//18667080-TableS9
GeneSigDB//18679425-Table3
Old_CMAP_down//bisacodyl-5677
Old_CMAP_down//perphenazine-1956
Old_CMAP_down//ellipticine-6253
Old_CMAP_down//nilutamide-5362
Old_CMAP_down//amoxapine-1931
Old_CMAP_down//ipratropium bromide-1769
Old_CMAP_down//doxorubicin-3291
Old_CMAP_down//oxetacaine-1903
Old_CMAP_down//metformin-2
GeneSigDB//18081427-TableS7
Old_CMAP_down//domperidone-2655
Old_CMAP_down//bromocriptine-1925
Old_CMAP_up//brinzolamide-1615
Old_CMAP_down//bambuterol-1582
Old_CMAP_down//apomorphine-1923
Old_CMAP_down//bupropion-1564

E

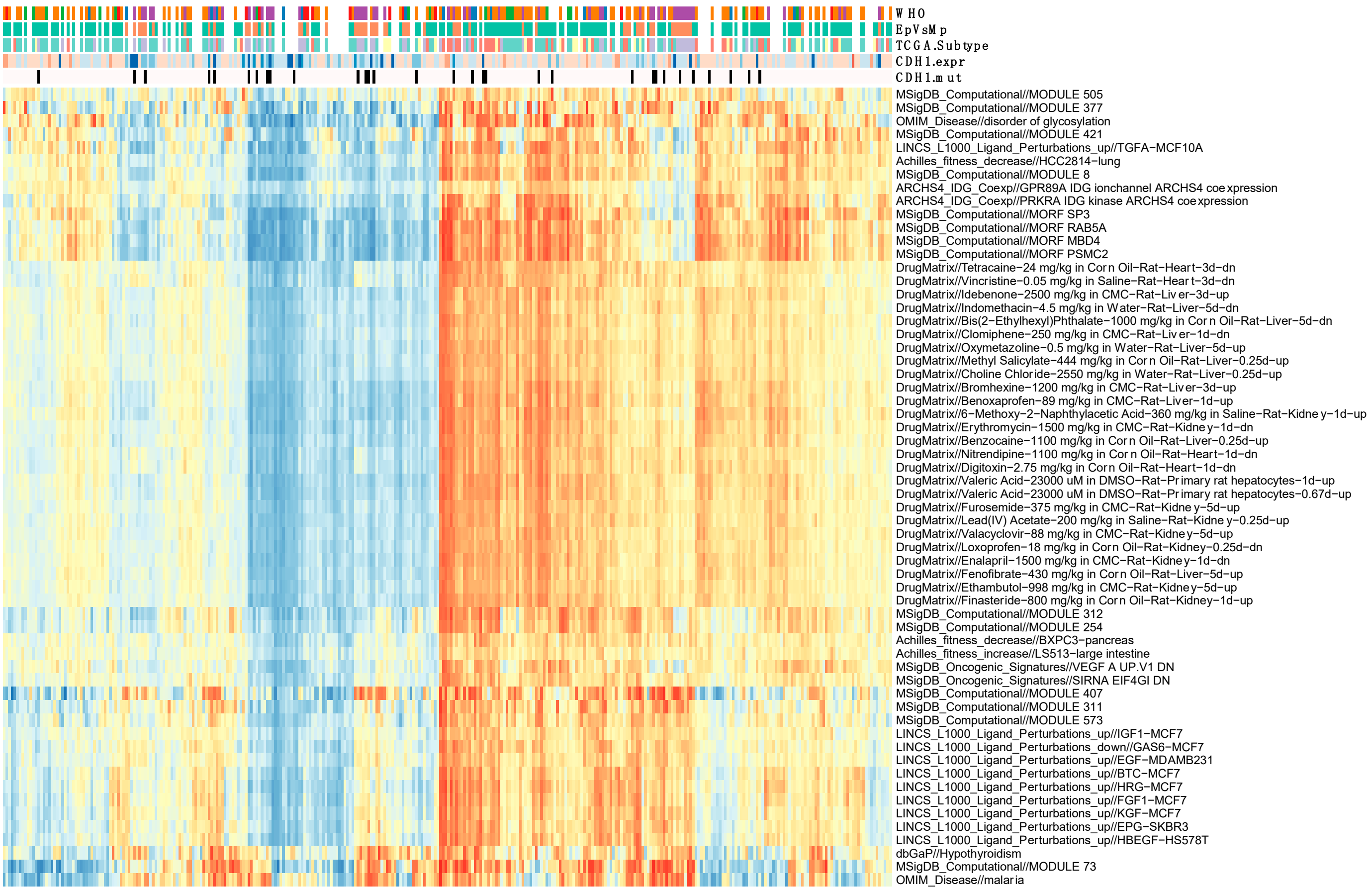
WHO
EpVsMp
TCGA.Subtype
CDH1.expr
CDH1.mut
MSigDB_Computational//MODULE 505
MSigDB_Computational//MODULE 377
OMIM_Disease//disorder of glycosylation
MSigDB_Computational//MODULE 421
LINCS_L1000_Ligand_Perturbations_up//TGFA−MCF10A
Achilles_fitness_decrease//HCC2814−lung
MSigDB_Computational//MODULE 8
ARCHS4_IDG_Coexp//GPR89A IDG ionchannel ARCHS4 coexpression
ARCHS4_IDG_Coexp//PRKRA IDG kinase ARCHS4 coexpression
MSigDB_Computational//MORF SP3
MSigDB_Computational//MORF RAB5A
MSigDB_Computational//MORF MBD4
MSigDB_Computational//MORF PSMC2
DrugMatrix//Tetracaine−24 mg/kg in Corn Oil−Rat−Heart−3d−dn
DrugMatrix//Vincristine−0.05 mg/kg in Saline−Rat−Heart−3d−dn
DrugMatrix//Idebenone−2500 mg/kg in CMC−Rat−Liver−3d−up
DrugMatrix//Indomethacin−4.5 mg/kg in Water−Rat−Liver−5d−dn
DrugMatrix//Bis(2−Ethylhexyl)Phthalate−1000 mg/kg in Corn Oil−Rat−Liver−5d−dn
DrugMatrix//Clomiphene−250 mg/kg in CMC−Rat−Liver−1d−dn
DrugMatrix//Oxymetazoline−0.5 mg/kg in Water−Rat−Liver−5d−up
DrugMatrix//Methyl Salicylate−444 mg/kg in Corn Oil−Rat−Liver−0.25d−up
DrugMatrix//Choline Chloride−2550 mg/kg in Water−Rat−Liver−0.25d−up
DrugMatrix//Bromhexine−1200 mg/kg in CMC−Rat−Liver−3d−up
DrugMatrix//Benoxaprofen−89 mg/kg in CMC−Rat−Liver−1d−up
DrugMatrix//6−Methoxy−2−Naphthylacetic Acid−360 mg/kg in Saline−Rat−Kidney−1d−up
DrugMatrix//Erythromycin−1500 mg/kg in CMC−Rat−Kidney−1d−dn
DrugMatrix//Benzocaine−1100 mg/kg in Corn Oil−Rat−Liver−0.25d−up
DrugMatrix//Nitrendipine−1100 mg/kg in Corn Oil−Rat−Heart−1d−dn
DrugMatrix//Digitoxin−2.75 mg/kg in Corn Oil−Rat−Heart−1d−dn
DrugMatrix//Valeric Acid−23000 uM in DMSO−Rat−Primary rat hepatocytes−1d−up
DrugMatrix//Valeric Acid−23000 uM in DMSO−Rat−Primary rat hepatocytes−0.67d−up
DrugMatrix//Furosemide−375 mg/kg in CMC−Rat−Kidney−5d−up
DrugMatrix//Lead(IV) Acetate−200 mg/kg in Saline−Rat−Kidney−0.25d−up
DrugMatrix//Valacyclovir−88 mg/kg in CMC−Rat−Kidney−5d−up
DrugMatrix//Loxoprofen−18 mg/kg in Corn Oil−Rat−Kidney−0.25d−dn
DrugMatrix//Enalapril−1500 mg/kg in CMC−Rat−Kidney−1d−dn
DrugMatrix//Fenofibrate−430 mg/kg in Corn Oil−Rat−Liver−5d−up
DrugMatrix//Ethambutol−998 mg/kg in CMC−Rat−Kidney−5d−up
DrugMatrix//Finasteride−800 mg/kg in Corn Oil−Rat−Kidney−1d−up
MSigDB_Computational//MODULE 312
MSigDB_Computational//MODULE 254
Achilles_fitness_decrease//BXPC3−pancreas
Achilles_fitness_increase//LS513−large intestine
MSigDB_Oncogenic_Signatures//VEGF A UP.V1 DN
MSigDB_Oncogenic_Signatures//SIRNA EIF4GI DN
MSigDB_Computational//MODULE 407
MSigDB_Computational//MODULE 311
MSigDB_Computational//MODULE 573
LINCS_L1000_Ligand_Perturbations_up//IGF1−MCF7
LINCS_L1000_Ligand_Perturbations_down//GAS6−MCF7
LINCS_L1000_Ligand_Perturbations_up//EGF−MDAMB231
LINCS_L1000_Ligand_Perturbations_up//BTC−MCF7
LINCS_L1000_Ligand_Perturbations_up//HRG−MCF7
LINCS_L1000_Ligand_Perturbations_up//FGF1−MCF7
LINCS_L1000_Ligand_Perturbations_up//KGF−MCF7
LINCS_L1000_Ligand_Perturbations_up//EPG−SKBR3
LINCS_L1000_Ligand_Perturbations_up//HBEGF−HS578T
dbGaP//Hypothyroidism
MSigDB_Computational//MODULE 73
OMIM_Disease//malaria

F

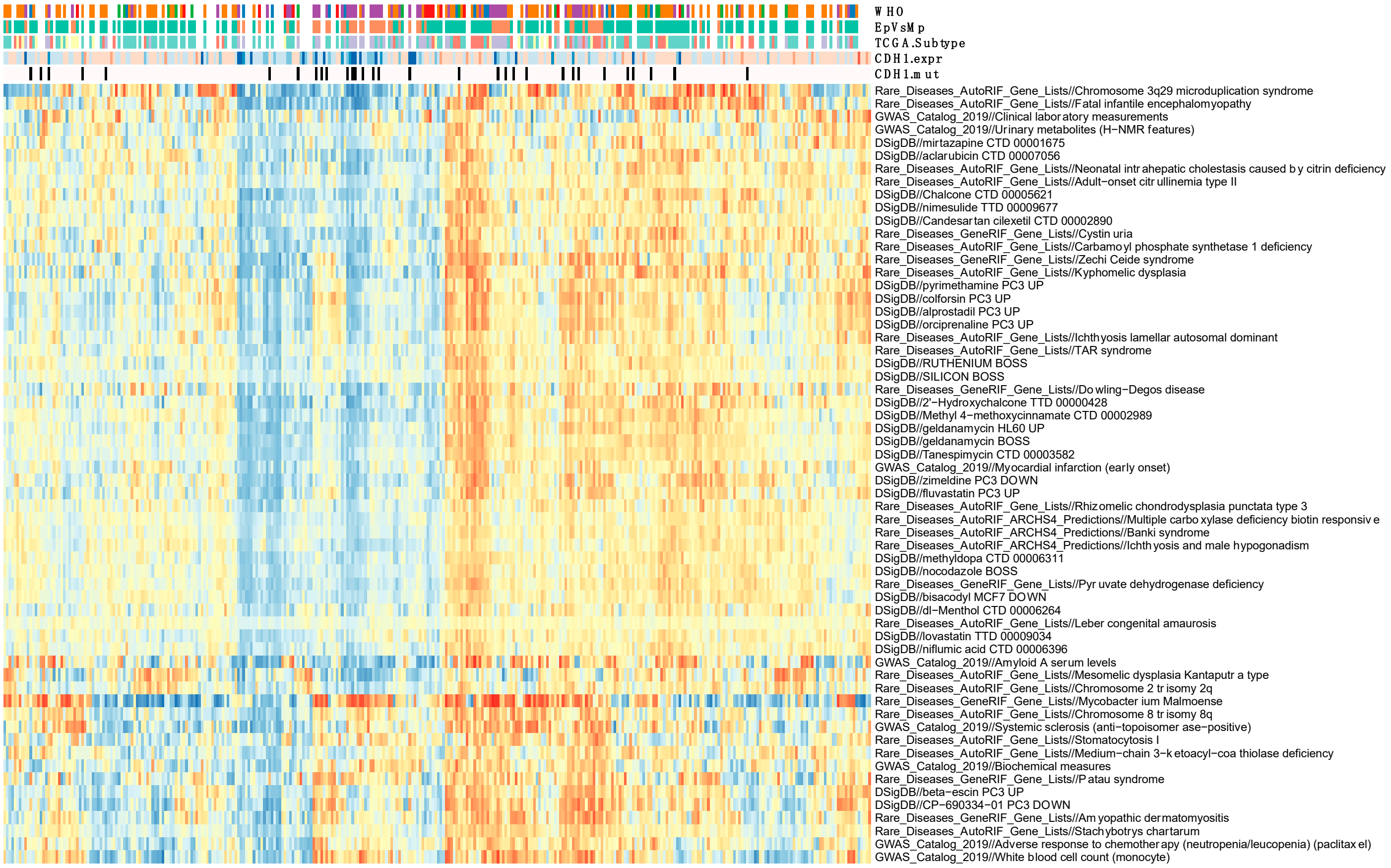
WHO
EpVsMp
TCGA.Subtype
CDH1.expr
CDH1.mut
Rare_Diseases_AutoRIF_Gene_Lists//Chromosome 3q29 microduplication syndrome
Rare_Diseases_AutoRIF_Gene_Lists//Fatal infantile encephalomyopathy
GWAS_Catalog_2019//Clinical laboratory measurements
GWAS_Catalog_2019//Urinary metabolites (H−NMR features)
DSigDB//mirtazapine CTD 00001675
DSigDB//aclarubicin CTD 00007056
Rare_Diseases_AutoRIF_Gene_Lists//Neonatal intrahepatic cholestasis caused by citrin deficiency
Rare_Diseases_AutoRIF_Gene_Lists//Adult−onset citrullinemia type II
DSigDB//Chalcone CTD 00005621
DSigDB//nimesulide TTD 00009677
DSigDB//Candesartan cilexetil CTD 00002890
Rare_Diseases_GeneRIF_Gene_Lists//Cystinuria
Rare_Diseases_AutoRIF_Gene_Lists//Carbamoyl phosphate synthetase 1 deficiency
Rare_Diseases_GeneRIF_Gene_Lists//Zechi Ceide syndrome
Rare_Diseases_AutoRIF_Gene_Lists//Kyphomelic dysplasia
DSigDB//pyrimethamine PC3 UP
DSigDB//colforsin PC3 UP
DSigDB//alprostadil PC3 UP
DSigDB//orciprenaline PC3 UP
Rare_Diseases_AutoRIF_Gene_Lists//Ichthyosis lamellar autosomal dominant
Rare_Diseases_AutoRIF_Gene_Lists//TAR syndrome
DSigDB//RUTHENIUM BOSS
DSigDB//SILICON BOSS
Rare_Diseases_GeneRIF_Gene_Lists//Dowling−Degos disease
DSigDB//2'−Hydroxychalcone TTD 00000428
DSigDB//Methyl 4−methoxycinnamate CTD 00002989
DSigDB//geldanamycin HL60 UP
DSigDB//geldanamycin BOSS
DSigDB//Tanespimycin CTD 00003582
GWAS_Catalog_2019//Myocardial infarction (early onset)
DSigDB//zimeldine PC3 DOWN
DSigDB//fluvastatin PC3 UP
Rare_Diseases_AutoRIF_Gene_Lists//Rhizomelic chondrodysplasia punctata type 3
Rare_Diseases_AutoRIF_ARCHS4_Predictions//Multiple carboxylase deficiency biotin responsive
Rare_Diseases_AutoRIF_ARCHS4_Predictions//Banki syndrome
Rare_Diseases_AutoRIF_ARCHS4_Predictions//Ichthyosis and male hypogonadism
DSigDB//methyldopa CTD 00006311
DSigDB//nocodazole BOSS
Rare_Diseases_GeneRIF_Gene_Lists//Pyruvate dehydrogenase deficiency
DSigDB//bisacodyl MCF7 DOWN
DSigDB//dl−Menthol CTD 00006264
Rare_Diseases_AutoRIF_Gene_Lists//Leber congenital amaurosis
DSigDB//lovastatin TTD 00009034
DSigDB//niflumic acid CTD 00006396
GWAS_Catalog_2019//Amyloid A serum levels
Rare_Diseases_AutoRIF_Gene_Lists//Mesomelic dysplasia Kantaputra type
Rare_Diseases_AutoRIF_Gene_Lists//Chromosome 2 trisomy 2q
Rare_Diseases_GeneRIF_Gene_Lists//Mycobacterium Malmoense
Rare_Diseases_AutoRIF_Gene_Lists//Chromosome 8 trisomy 8q
GWAS_Catalog_2019//Systemic sclerosis (anti−topoisomerase−positive)
Rare_Diseases_AutoRIF_Gene_Lists//Stomatocytosis I
Rare_Diseases_AutoRIF_Gene_Lists//Medium−chain 3−ketoacyl−coa thiolase deficiency
GWAS_Catalog_2019//Biochemical measures
Rare_Diseases_GeneRIF_Gene_Lists//Patau syndrome
DSigDB//beta−escin PC3 UP
DSigDB//CP−690334−01 PC3 DOWN
Rare_Diseases_GeneRIF_Gene_Lists//Amyopathic dermatomyositis
Rare_Diseases_AutoRIF_Gene_Lists//Stachybotrys chartarum
GWAS_Catalog_2019//Adverse response to chemotherapy (neutropenia/leucopenia) (paclitaxel)
GWAS_Catalog_2019//White blood cell count (monocyte)

G

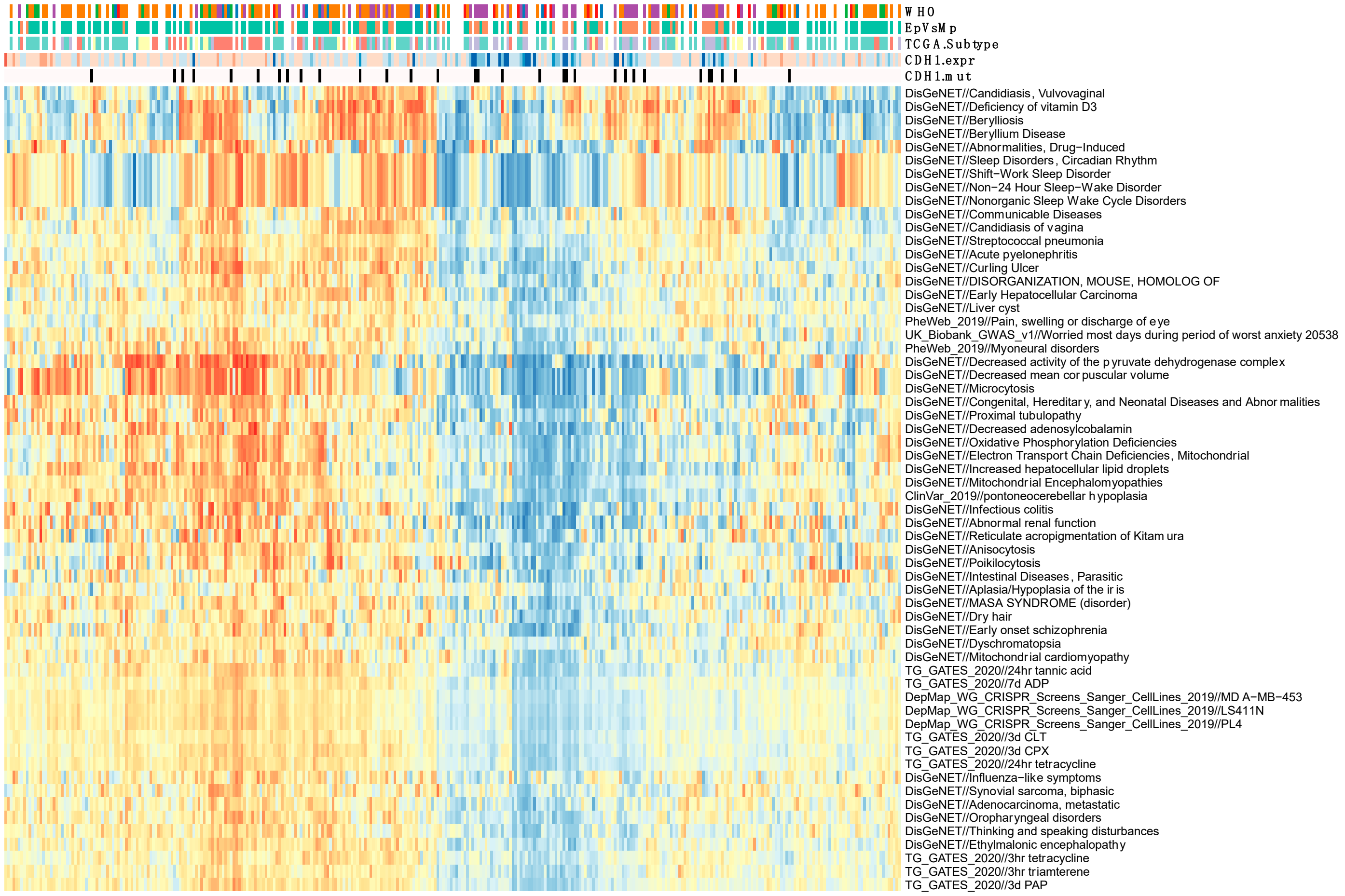

WHO
EpVsMp
TCGA.Subtype
CDH1.expr
CDH1.mut
DisGeNET//Candidiasis, Vulvovaginal
DisGeNET//Deficiency of vitamin D3
DisGeNET//Berylliosis
DisGeNET//Beryllium Disease
DisGeNET//Abnormalities, Drug−Induced
DisGeNET//Sleep Disorders, Circadian Rhythm
DisGeNET//Shift−Work Sleep Disorder
DisGeNET//Non−24 Hour Sleep−Wake Disorder
DisGeNET//Nonorganic Sleep Wake Cycle Disorders
DisGeNET//Communicable Diseases
DisGeNET//Candidiasis of vagina
DisGeNET//Streptococcal pneumonia
DisGeNET//Acute pyelonephritis
DisGeNET//Curling Ulcer
DisGeNET//DISORGANIZATION, MOUSE, HOMOLOG OF
DisGeNET//Early Hepatocellular Carcinoma
DisGeNET//Liver cyst
PheWeb_2019//Pain, swelling or discharge of eye
UK_Biobank_GWAS_v1//Worried most days during period of worst anxiety 20538
PheWeb_2019//Myoneural disorders
DisGeNET//Decreased activity of the pyruvate dehydrogenase complex
DisGeNET//Decreased mean corpuscular volume
DisGeNET//Microcytosis
DisGeNET//Congenital, Hereditary, and Neonatal Diseases and Abnormalities
DisGeNET//Proximal tubulopathy
DisGeNET//Decreased adenosylcobalamin
DisGeNET//Oxidative Phosphorylation Deficiencies
DisGeNET//Electron Transport Chain Deficiencies, Mitochondrial
DisGeNET//Increased hepatocellular lipid droplets
DisGeNET//Mitochondrial Encephalomyopathies
ClinVar_2019//pontoneocerebellar hypoplasia
DisGeNET//Infectious colitis
DisGeNET//Abnormal renal function
DisGeNET//Reticulate acropigmentation of Kitamura
DisGeNET//Anisocytosis
DisGeNET//Poikilocytosis
DisGeNET//Intestinal Diseases, Parasitic
DisGeNET//Aplasia/Hypoplasia of the iris
DisGeNET//MASA SYNDROME (disorder)
DisGeNET//Dry hair
DisGeNET//Early onset schizophrenia
DisGeNET//Dyschromatopsia
DisGeNET//Mitochondrial cardiomyopathy
TG_GATES_2020//24hr tannic acid
TG_GATES_2020//7d ADP
DepMap_WG_CRISPR_Screens_Sanger_CellLines_2019//MDA−MB−453
DepMap_WG_CRISPR_Screens_Sanger_CellLines_2019//LS411N
DepMap_WG_CRISPR_Screens_Sanger_CellLines_2019//PL4
TG_GATES_2020//3d CLT
TG_GATES_2020//3d CPX
TG_GATES_2020//24hr tetracycline
DisGeNET//Influenza−like symptoms
DisGeNET//Synovial sarcoma, biphasic
DisGeNET//Adenocarcinoma, metastatic
DisGeNET//Oropharyngeal disorders
DisGeNET//Thinking and speaking disturbances
DisGeNET//Ethylmalonic encephalopathy
TG_GATES_2020//3hr tetracycline
TG_GATES_2020//3hr triamterene
TG_GATES_2020//3d PAP

H

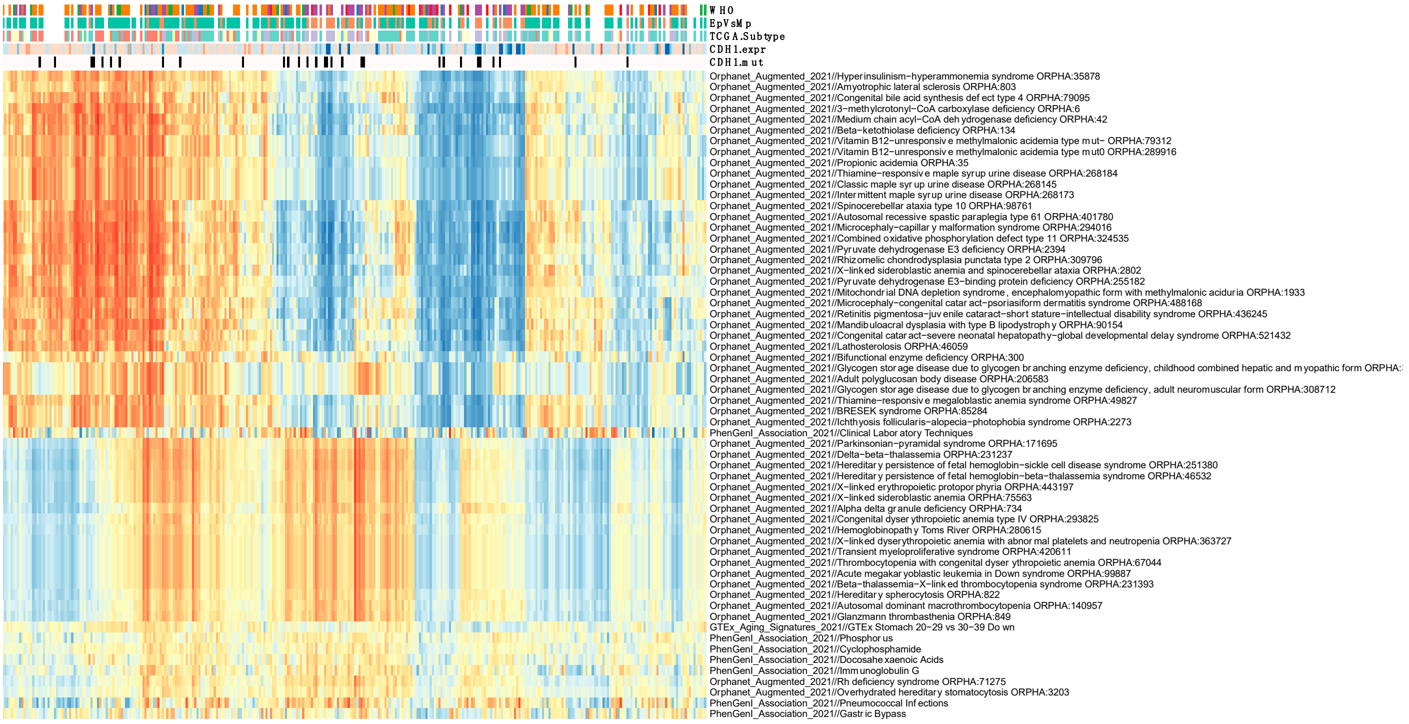

WHO
EpVsMp
TCGA.Subtype
CDH1.expr
CDH1.mut
Orphanet_Augmented_2021//Hyperinsulinism−hyperammonemia syndrome ORPHA:35878
Orphanet_Augmented_2021//Amyotrophic lateral sclerosis ORPHA:803
Orphanet_Augmented_2021//Congenital bile acid synthesis defect type 4 ORPHA:79095
Orphanet_Augmented_2021//3−methylcrotonyl−CoA carboxylase deficiency ORPHA:6
Orphanet_Augmented_2021//Medium chain acyl−CoA dehydrogenase deficiency ORPHA:42
Orphanet_Augmented_2021//Beta−ketothiolase deficiency ORPHA:134
Orphanet_Augmented_2021//Vitamin B12−unresponsive methylmalonic acidemia type mut− ORPHA:79312
Orphanet_Augmented_2021//Vitamin B12−unresponsive methylmalonic acidemia type mut0 ORPHA:289916
Orphanet_Augmented_2021//Propionic acidemia ORPHA:35
Orphanet_Augmented_2021//Thiamine−responsive maple syrup urine disease ORPHA:268184
Orphanet_Augmented_2021//Classic maple syrup urine disease ORPHA:268145
Orphanet_Augmented_2021//Intermittent maple syrup urine disease ORPHA:268173
Orphanet_Augmented_2021//Spinocerebellar ataxia type 10 ORPHA:98761
Orphanet_Augmented_2021//Autosomal recessive spastic paraplegia type 61 ORPHA:401780
Orphanet_Augmented_2021//Microcephaly−capillary malformation syndrome ORPHA:294016
Orphanet_Augmented_2021//Combined oxidative phosphorylation defect type 11 ORPHA:324535
Orphanet_Augmented_2021//Pyruvate dehydrogenase E3 deficiency ORPHA:2394
Orphanet_Augmented_2021//Rhizomelic chondrodysplasia punctata type 2 ORPHA:309796
Orphanet_Augmented_2021//X−linked sideroblastic anemia and spinocerebellar ataxia ORPHA:2802
Orphanet_Augmented_2021//Pyruvate dehydrogenase E3−binding protein deficiency ORPHA:255182
Orphanet_Augmented_2021//Mitochondrial DNA depletion syndrome, encephalomyopathic form with methylmalonic aciduria ORPHA:1933
Orphanet_Augmented_2021//Microcephaly−congenital cataract−psoriasiform dermatitis syndrome ORPHA:488168
Orphanet_Augmented_2021//Retinitis pigmentosa−juvenile cataract−short stature−intellectual disability syndrome ORPHA:436245
Orphanet_Augmented_2021//Mandibuloacral dysplasia with type B lipodystrophy ORPHA:90154
Orphanet_Augmented_2021//Congenital cataract−severe neonatal hepatopathy−global developmental delay syndrome ORPHA:521432
Orphanet_Augmented_2021//Lathosterolosis ORPHA:46059
Orphanet_Augmented_2021//Bifunctional enzyme deficiency ORPHA:300
Orphanet_Augmented_2021//Glycogen storage disease due to glycogen branching enzyme deficiency, childhood combined hepatic and myopathic form ORPHA:
Orphanet_Augmented_2021//Adult polyglucosan body disease ORPHA:206583
Orphanet_Augmented_2021//Glycogen storage disease due to glycogen branching enzyme deficiency, adult neuromuscular form ORPHA:308712
Orphanet_Augmented_2021//Thiamine−responsive megaloblastic anemia syndrome ORPHA:49827
Orphanet_Augmented_2021//BRESEK syndrome ORPHA:85284
Orphanet_Augmented_2021//Ichthyosis follicularis−alopecia−photophobia syndrome ORPHA:2273
PhenGenI_Association_2021//Clinical Laboratory Techniques
Orphanet_Augmented_2021//Parkinsonian−pyramidal syndrome ORPHA:171695
Orphanet_Augmented_2021//Delta−beta−thalassemia ORPHA:231237
Orphanet_Augmented_2021//Hereditary persistence of fetal hemoglobin−sickle cell disease syndrome ORPHA:251380
Orphanet_Augmented_2021//Hereditary persistence of fetal hemoglobin−beta−thalassemia syndrome ORPHA:46532
Orphanet_Augmented_2021//X−linked erythropoietic protoporphyria ORPHA:443197
Orphanet_Augmented_2021//X−linked sideroblastic anemia ORPHA:75563
Orphanet_Augmented_2021//Alpha delta granule deficiency ORPHA:734
Orphanet_Augmented_2021//Congenital dyserythropoietic anemia type IV ORPHA:293825
Orphanet_Augmented_2021//Hemoglobinopathy Toms River ORPHA:280615
Orphanet_Augmented_2021//X−linked dyserythropoietic anemia with abnormal platelets and neutropenia ORPHA:363727
Orphanet_Augmented_2021//Transient myeloproliferative syndrome ORPHA:420611
Orphanet_Augmented_2021//Thrombocytopenia with congenital dyserythropoietic anemia ORPHA:67044
Orphanet_Augmented_2021//Acute megakaryoblastic leukemia in Down syndrome ORPHA:99887
Orphanet_Augmented_2021//Beta−thalassemia−X−linked thrombocytopenia syndrome ORPHA:231393
Orphanet_Augmented_2021//Hereditary spherocytosis ORPHA:822
Orphanet_Augmented_2021//Autosomal dominant macrothrombocytopenia ORPHA:140957
Orphanet_Augmented_2021//Glanzmann thrombasthenia ORPHA:849
GTEx_Aging_Signatures_2021//GTEx Stomach 20−29 vs 30−39 Down
PhenGenI_Association_2021//Phosphorus
PhenGenI_Association_2021//Cyclophosphamide
PhenGenI_Association_2021//Docosahexaenoic Acids
PhenGenI_Association_2021//Immunoglobulin G
Orphanet_Augmented_2021//Rh deficiency syndrome ORPHA:71275
Orphanet_Augmented_2021//Overhydrated hereditary stomatocytosis ORPHA:3203
PhenGenI_Association_2021//Pneumococcal Infections
PhenGenI_Association_2021//Gastric Bypass

I

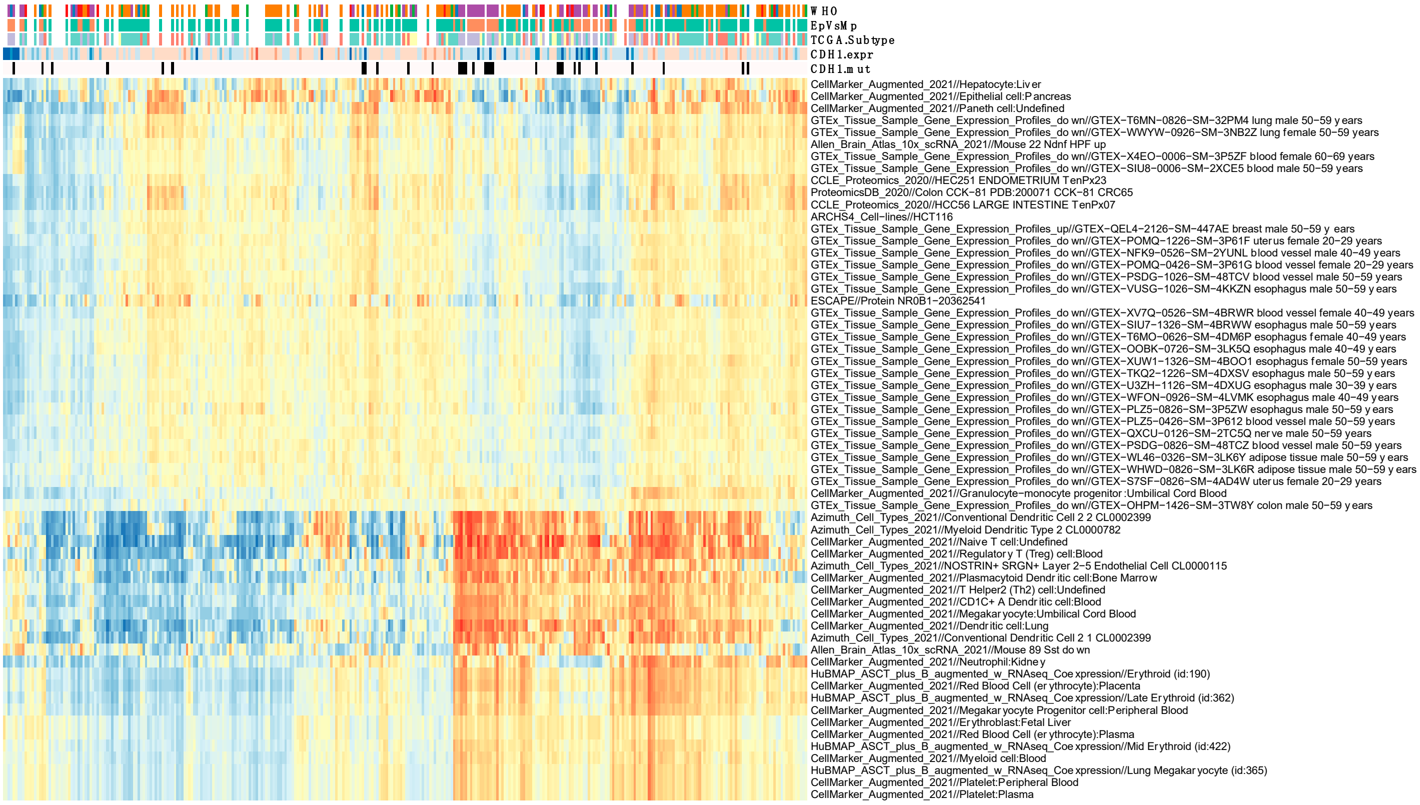
WHO
EpVsMp
TCGA.Subtype
CDH1.expr
CDH1.mut
CellMarker_Augmented_2021//Hepatocyte:Liver
CellMarker_Augmented_2021//Epithelial cell:Pancreas
CellMarker_Augmented_2021//Paneth cell:Undefined
GTEx_Tissue_Sample_Gene_Expression_Profiles_down//GTEX-T6MN-0826-SM-32PM4 lung male 50-59 years
GTEx_Tissue_Sample_Gene_Expression_Profiles_down//GTEX-WWYW-0926-SM-3NB2Z lung female 50-59 years
Allen_Brain_Atlas_10x_scRNA_2021//Mouse 22 Ndnf HPF up
GTEx_Tissue_Sample_Gene_Expression_Profiles_down//GTEX-X4EO-0006-SM-3P5ZF blood female 60-69 years
GTEx_Tissue_Sample_Gene_Expression_Profiles_down//GTEX-SIU8-0006-SM-2XCE5 blood male 50-59 years
CCLE_Proteomics_2020//HEC251 ENDOMETRIUM TenPx23
ProteomicsDB_2020//Colon CCK-81 PDB:200071 CCK-81 CRC65
CCLE_Proteomics_2020//HCC56 LARGE INTESTINE TenPx07
ARCHS4_Cell-lines//HCT116
GTEx_Tissue_Sample_Gene_Expression_Profiles_up//GTEX-QEL4-2126-SM-447AE breast male 50-59 years
GTEx_Tissue_Sample_Gene_Expression_Profiles_down//GTEX-POMQ-1226-SM-3P61F uterus female 20-29 years
GTEx_Tissue_Sample_Gene_Expression_Profiles_down//GTEX-NFK9-0526-SM-2YUNL blood vessel male 40-49 years
GTEx_Tissue_Sample_Gene_Expression_Profiles_down//GTEX-POMQ-0426-SM-3P61G blood vessel female 20-29 years
GTEx_Tissue_Sample_Gene_Expression_Profiles_down//GTEX-PSDG-1026-SM-48TCV blood vessel male 50-59 years
GTEx_Tissue_Sample_Gene_Expression_Profiles_down//GTEX-VUSG-1026-SM-4KKZN esophagus male 50-59 years
ESCAPE//Protein NR0B1-20362541
GTEx_Tissue_Sample_Gene_Expression_Profiles_down//GTEX-XV7Q-0526-SM-4BRWR blood vessel female 40-49 years
GTEx_Tissue_Sample_Gene_Expression_Profiles_down//GTEX-SIU7-1326-SM-4BRWW esophagus male 50-59 years
GTEx_Tissue_Sample_Gene_Expression_Profiles_down//GTEX-T6MO-0626-SM-4DM6P esophagus female 40-49 years
GTEx_Tissue_Sample_Gene_Expression_Profiles_down//GTEX-OOBK-0726-SM-3LK5Q esophagus male 40-49 years
GTEx_Tissue_Sample_Gene_Expression_Profiles_down//GTEX-XUW1-1326-SM-4BOO1 esophagus female 50-59 years
GTEx_Tissue_Sample_Gene_Expression_Profiles_down//GTEX-TKQ2-1226-SM-4DXSV esophagus male 50-59 years
GTEx_Tissue_Sample_Gene_Expression_Profiles_down//GTEX-U3ZH-1126-SM-4DXUG esophagus male 30-39 years
GTEx_Tissue_Sample_Gene_Expression_Profiles_down//GTEX-WFON-0926-SM-4LVMK esophagus male 40-49 years
GTEx_Tissue_Sample_Gene_Expression_Profiles_down//GTEX-PLZ5-0826-SM-3P5ZW esophagus male 50-59 years
GTEx_Tissue_Sample_Gene_Expression_Profiles_down//GTEX-PLZ5-0426-SM-3P612 blood vessel male 50-59 years
GTEx_Tissue_Sample_Gene_Expression_Profiles_down//GTEX-QXCU-0126-SM-2TC5Q nerve male 50-59 years
GTEx_Tissue_Sample_Gene_Expression_Profiles_down//GTEX-PSDG-0826-SM-48TCZ blood vessel male 50-59 years
GTEx_Tissue_Sample_Gene_Expression_Profiles_down//GTEX-WL46-0326-SM-3LK6Y adipose tissue male 50-59 years
GTEx_Tissue_Sample_Gene_Expression_Profiles_down//GTEX-WHWD-0826-SM-3LK6R adipose tissue male 50-59 years
GTEx_Tissue_Sample_Gene_Expression_Profiles_down//GTEX-S7SF-0826-SM-4AD4W uterus female 20-29 years
CellMarker_Augmented_2021//Granulocyte-monocyte progenitor:Umbilical Cord Blood
GTEx_Tissue_Sample_Gene_Expression_Profiles_down//GTEX-OHPM-1426-SM-3TW8Y colon male 50-59 years
Azimuth_Cell_Types_2021//Conventional Dendritic Cell 2 2 CL0002399
Azimuth_Cell_Types_2021//Myeloid Dendritic Type 2 CL0000782
CellMarker_Augmented_2021//Naive T cell:Undefined
CellMarker_Augmented_2021//Regulatory T (Treg) cell:Blood
Azimuth_Cell_Types_2021//NOSTRIN+ SRGN+ Layer 2-5 Endothelial Cell CL0000115
CellMarker_Augmented_2021//Plasmacytoid Dendritic cell:Bone Marrow
CellMarker_Augmented_2021//T Helper2 (Th2) cell:Undefined
CellMarker_Augmented_2021//CD1C+ A Dendritic cell:Blood
CellMarker_Augmented_2021//Megakaryocyte:Umbilical Cord Blood
CellMarker_Augmented_2021//Dendritic cell:Lung
Azimuth_Cell_Types_2021//Conventional Dendritic Cell 2 1 CL0002399
Allen_Brain_Atlas_10x_scRNA_2021//Mouse 89 Sst down
CellMarker_Augmented_2021//Neutrophil:Kidney
HuBMAP_ASCT_plus_B_augmented_w_RNAseq_Coexpression//Erythroid (id:190)
CellMarker_Augmented_2021//Red Blood Cell (erythrocyte):Placenta
HuBMAP_ASCT_plus_B_augmented_w_RNAseq_Coexpression//Late Erythroid (id:362)
CellMarker_Augmented_2021//Megakaryocyte Progenitor cell:Peripheral Blood
CellMarker_Augmented_2021//Erythroblast:Fetal Liver
CellMarker_Augmented_2021//Red Blood Cell (erythrocyte):Plasma
HuBMAP_ASCT_plus_B_augmented_w_RNAseq_Coexpression//Mid Erythroid (id:422)
CellMarker_Augmented_2021//Myeloid cell:Blood
HuBMAP_ASCT_plus_B_augmented_w_RNAseq_Coexpression//Lung Megakaryocyte (id:365)
CellMarker_Augmented_2021//Platelet:Peripheral Blood
CellMarker_Augmented_2021//Platelet:Plasma

L

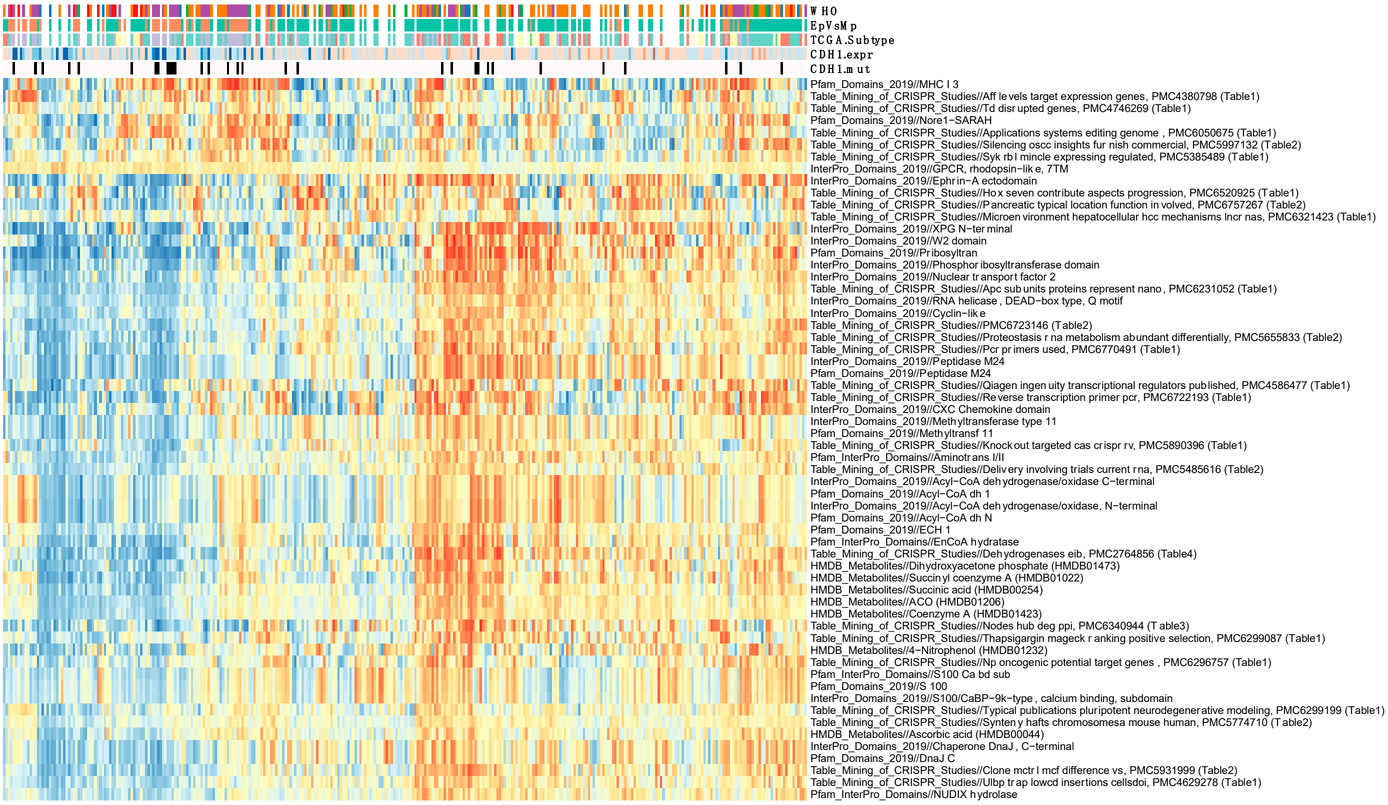
WHO
EpVsMp
TCGA.Subtype
CDH1.expr
CDH1.mut
Pfam_Domains_2019//MHC I 3
Table_Mining_of_CRISPR_Studies//Aff levels target expression genes, PMC4380798 (Table1)
Table_Mining_of_CRISPR_Studies//Td disrupted genes, PMC4746269 (Table1)
Pfam_Domains_2019//Nore1-SARAH
Table_Mining_of_CRISPR_Studies//Applications systems editing genome , PMC6050675 (Table1)
Table_Mining_of_CRISPR_Studies//Silencing oscc insights fur nish commercial, PMC5997132 (Table2)
Table_Mining_of_CRISPR_Studies//Syk rbl mincle expressing regulated, PMC5385489 (Table1)
InterPro_Domains_2019//GPCR, rhodopsin-like, 7TM
InterPro_Domains_2019//Ephrin-A ectodomain
Table_Mining_of_CRISPR_Studies//Hox seven contribute aspects progression, PMC6520925 (Table1)
Table_Mining_of_CRISPR_Studies//Pancreatic typical location function in volved, PMC6757267 (Table2)
Table_Mining_of_CRISPR_Studies//Microenvironment hepatocellular hcc mechanisms lncr nas, PMC6321423 (Table1)
InterPro_Domains_2019//XPG N-terminal
InterPro_Domains_2019//W2 domain
Pfam_Domains_2019//Pribosyltran
InterPro_Domains_2019//Phosphoribosyltransferase domain
InterPro_Domains_2019//Nuclear transport factor 2
Table_Mining_of_CRISPR_Studies//Apc subunits proteins represent nano, PMC6231052 (Table1)
InterPro_Domains_2019//RNA helicase , DEAD-box type, Q motif
InterPro_Domains_2019//Cyclin-like
Table_Mining_of_CRISPR_Studies//PMC6723146 (Table2)
Table_Mining_of_CRISPR_Studies//Proteostasis r na metabolism abundant differentially, PMC5655833 (Table2)
Table_Mining_of_CRISPR_Studies//Pcr primers used, PMC6770491 (Table1)
InterPro_Domains_2019//Peptidase M24
Pfam_Domains_2019//Peptidase M24
Table_Mining_of_CRISPR_Studies//Qiagen ingenuity transcriptional regulators published, PMC4586477 (Table1)
Table_Mining_of_CRISPR_Studies//Reverse transcription primer pcr, PMC6722193 (Table1)
InterPro_Domains_2019//CXC Chemokine domain
InterPro_Domains_2019//Methyltransferase type 11
Pfam_Domains_2019//Methyltransf 11
Table_Mining_of_CRISPR_Studies//Knockout targeted cas crispr rv, PMC5890396 (Table1)
Pfam_InterPro_Domains//Aminotrans I/II
Table_Mining_of_CRISPR_Studies//Delivery involving trials current rna, PMC5485616 (Table2)
InterPro_Domains_2019//Acyl-CoA dehydrogenase/oxidase C-terminal
Pfam_Domains_2019//Acyl-CoA dh 1
InterPro_Domains_2019//Acyl-CoA dehydrogenase/oxidase, N-terminal
Pfam_Domains_2019//Acyl-CoA dh N
Pfam_Domains_2019//ECH 1
Pfam_InterPro_Domains//EnCoA hydratase
Table_Mining_of_CRISPR_Studies//Dehydrogenases eib, PMC2764856 (Table4)
HMDB_Metabolites//Dihydroxyacetone phosphate (HMDB01473)
HMDB_Metabolites//Succinyl coenzyme A (HMDB01022)
HMDB_Metabolites//Succinic acid (HMDB00254)
HMDB_Metabolites//ACO (HMDB01206)
HMDB_Metabolites//Coenzyme A (HMDB01423)
Table_Mining_of_CRISPR_Studies//Nodes hub deg ppi, PMC6340944 (Table3)
Table_Mining_of_CRISPR_Studies//Thapsigargin mageck r anking positive selection, PMC6299087 (Table1)
HMDB_Metabolites//4-Nitrophenol (HMDB01232)
Table_Mining_of_CRISPR_Studies//Np oncogenic potential target genes , PMC6296757 (Table1)
Pfam_InterPro_Domains//S100 Ca bd sub
Pfam_Domains_2019//S 100
InterPro_Domains_2019//S100/CaBP-9k-type , calcium binding, subdomain
Table_Mining_of_CRISPR_Studies//Typical publications pluripotent neurodegenerative modeling, PMC6299199 (Table1)
Table_Mining_of_CRISPR_Studies//Synteny hafts chromosomesa mouse human, PMC5774710 (Table2)
HMDB_Metabolites//Ascorbic acid (HMDB00044)
InterPro_Domains_2019//Chaperone DnaJ , C-terminal
Pfam_Domains_2019//DnaJ C
Table_Mining_of_CRISPR_Studies//Clone mctrl mcf difference vs, PMC5931999 (Table2)
Table_Mining_of_CRISPR_Studies//Ulbp trap lowcd insertions cellsdoi, PMC4629278 (Table1)
Pfam_InterPro_Domains//NUDIX hydrolase

M

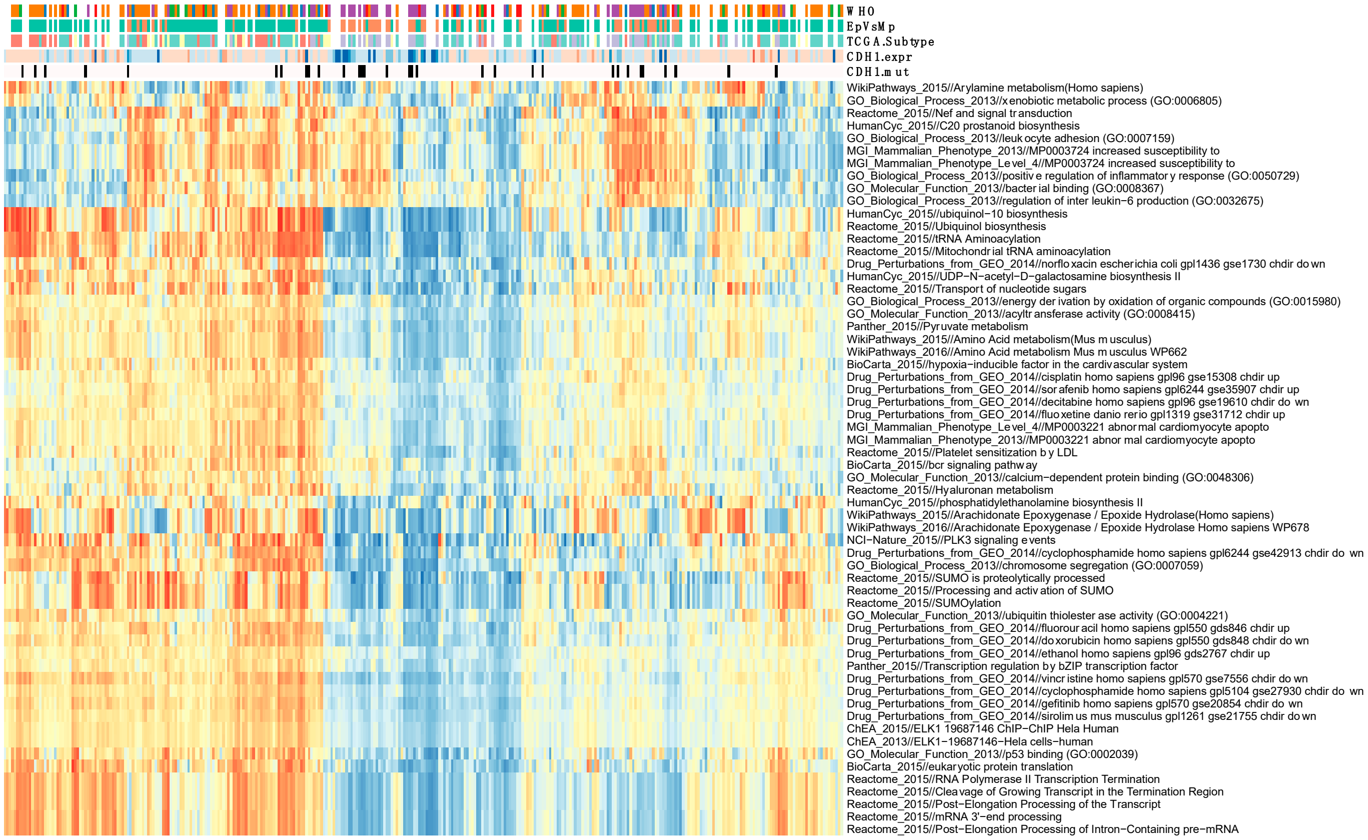
WHO
EpVsMp
TCGA.Subtype
CDH1.expr
CDH1.mut
WikiPathways_2015//Arylamine metabolism(Homo sapiens)
GO_Biological_Process_2013//xenobiotic metabolic process (GO:0006805)
Reactome_2015//Nef and signal transduction
HumanCyc_2015//C20 prostanoid biosynthesis
GO_Biological_Process_2013//leukocyte adhesion (GO:0007159)
MGI_Mammalian_Phenotype_2013//MP0003724 increased susceptibility to
MGI_Mammalian_Phenotype_Level_4//MP0003724 increased susceptibility to
GO_Biological_Process_2013//positive regulation of inflammatory response (GO:0050729)
GO_Molecular_Function_2013//bacterial binding (GO:0008367)
GO_Biological_Process_2013//regulation of interleukin−6 production (GO:0032675)
HumanCyc_2015//ubiquinol−10 biosynthesis
Reactome_2015//Ubiquinol biosynthesis
Reactome_2015//tRNA Aminoacylation
Reactome_2015//Mitochondrial tRNA aminoacylation
Drug_Perturbations_from_GEO_2014//norfloxacin escherichia coli gpl1436 gse1730 chdir down
HumanCyc_2015//UDP−N−acetyl−D−galactosamine biosynthesis II
Reactome_2015//Transport of nucleotide sugars
GO_Biological_Process_2013//energy derivation by oxidation of organic compounds (GO:0015980)
GO_Molecular_Function_2013//acyltransferase activity (GO:0008415)
Panther_2015//Pyruvate metabolism
WikiPathways_2015//Amino Acid metabolism(Mus musculus)
WikiPathways_2016//Amino Acid metabolism Mus musculus WP662
BioCarta_2015//hypoxia−inducible factor in the cardivascular system
Drug_Perturbations_from_GEO_2014//cisplatin homo sapiens gpl96 gse15308 chdir up
Drug_Perturbations_from_GEO_2014//sorafenib homo sapiens gpl6244 gse35907 chdir up
Drug_Perturbations_from_GEO_2014//decitabine homo sapiens gpl96 gse19610 chdir down
Drug_Perturbations_from_GEO_2014//fluoxetine danio rerio gpl1319 gse31712 chdir up
MGI_Mammalian_Phenotype_Level_4//MP0003221 abnormal cardiomyocyte apopto
MGI_Mammalian_Phenotype_2013//MP0003221 abnormal cardiomyocyte apopto
Reactome_2015//Platelet sensitization by LDL
BioCarta_2015//bcr signaling pathway
GO_Molecular_Function_2013//calcium−dependent protein binding (GO:0048306)
Reactome_2015//Hyaluronan metabolism
HumanCyc_2015//phosphatidylethanolamine biosynthesis II
WikiPathways_2015//Arachidonate Epoxygenase / Epoxide Hydrolase(Homo sapiens)
WikiPathways_2016//Arachidonate Epoxygenase / Epoxide Hydrolase Homo sapiens WP678
NCI−Nature_2015//PLK3 signaling events
Drug_Perturbations_from_GEO_2014//cyclophosphamide homo sapiens gpl6244 gse42913 chdir down
GO_Biological_Process_2013//chromosome segregation (GO:0007059)
Reactome_2015//SUMO is proteolytically processed
Reactome_2015//Processing and activation of SUMO
Reactome_2015//SUMOylation
GO_Molecular_Function_2013//ubiquitin thiolesterase activity (GO:0004221)
Drug_Perturbations_from_GEO_2014//fluorouracil homo sapiens gpl550 gds846 chdir up
Drug_Perturbations_from_GEO_2014//doxorubicin homo sapiens gpl550 gds848 chdir down
Drug_Perturbations_from_GEO_2014//ethanol homo sapiens gpl96 gds2767 chdir up
Panther_2015//Transcription regulation by bZIP transcription factor
Drug_Perturbations_from_GEO_2014//vincristine homo sapiens gpl570 gse7556 chdir down
Drug_Perturbations_from_GEO_2014//cyclophosphamide homo sapiens gpl5104 gse27930 chdir down
Drug_Perturbations_from_GEO_2014//gefitinib homo sapiens gpl570 gse20854 chdir down
Drug_Perturbations_from_GEO_2014//sirolimus mus musculus gpl1261 gse21755 chdir down
ChEA_2015//ELK1 19687146 ChIP−ChIP Hela Human
ChEA_2013//ELK1−19687146−Hela cells−human
GO_Molecular_Function_2013//p53 binding (GO:0002039)
BioCarta_2015//eukaryotic protein translation
Reactome_2015//RNA Polymerase II Transcription Termination
Reactome_2015//Cleavage of Growing Transcript in the Termination Region
Reactome_2015//Post−Elongation Processing of the Transcript
Reactome_2015//mRNA 3'−end processing
Reactome_2015//Post−Elongation Processing of Intron−Containing pre−mRNA

N

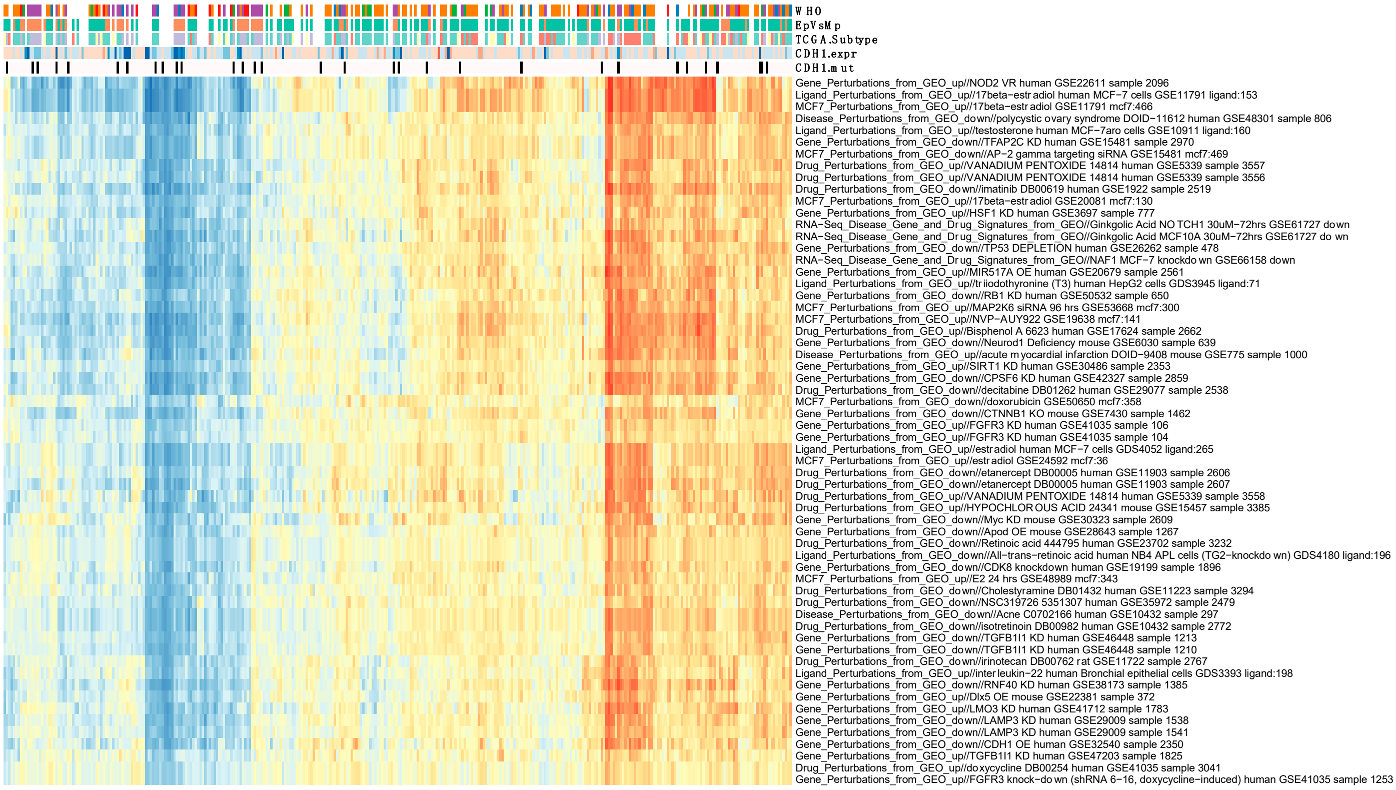
WHO
EpVsMp
TCGA.Subtype
CDH1.expr
CDH1.mut
Gene_Perturbations_from_GEO_up//NOD2 VR human GSE22611 sample 2096
Ligand_Perturbations_from_GEO_up//17beta−estradiol human MCF−7 cells GSE11791 ligand:153
MCF7_Perturbations_from_GEO_up//17beta−estradiol GSE11791 mcf7:466
Disease_Perturbations_from_GEO_down//polycystic ovary syndrome DOID−11612 human GSE48301 sample 806
Ligand_Perturbations_from_GEO_up//testosterone human MCF−7aro cells GSE10911 ligand:160
Gene_Perturbations_from_GEO_down//TFAP2C KD human GSE15481 sample 2970
MCF7_Perturbations_from_GEO_down//AP−2 gamma targeting siRNA GSE15481 mcf7:469
Drug_Perturbations_from_GEO_up//VANADIUM PENTOXIDE 14814 human GSE5339 sample 3557
Drug_Perturbations_from_GEO_up//VANADIUM PENTOXIDE 14814 human GSE5339 sample 3556
Drug_Perturbations_from_GEO_down//imatinib DB00619 human GSE1922 sample 2519
MCF7_Perturbations_from_GEO_up//17beta−estradiol GSE20081 mcf7:130
Gene_Perturbations_from_GEO_up//HSF1 KD human GSE3697 sample 777
RNA−Seq_Disease_Gene_and_Drug_Signatures_from_GEO//Ginkgolic Acid NOTCH1 30uM−72hrs GSE61727 down
RNA−Seq_Disease_Gene_and_Drug_Signatures_from_GEO//Ginkgolic Acid MCF10A 30uM−72hrs GSE61727 down
Gene_Perturbations_from_GEO_down//TP53 DEPLETION human GSE26262 sample 478
RNA−Seq_Disease_Gene_and_Drug_Signatures_from_GEO//NAF1 MCF−7 knockdown GSE66158 down
Gene_Perturbations_from_GEO_up//MIR517A OE human GSE20679 sample 2561
Ligand_Perturbations_from_GEO_up//triiodothyronine (T3) human HepG2 cells GDS3945 ligand:71
Gene_Perturbations_from_GEO_down//RB1 KD human GSE50532 sample 650
MCF7_Perturbations_from_GEO_up//MAP2K6 siRNA 96 hrs GSE53668 mcf7:300
MCF7_Perturbations_from_GEO_up//NVP−AUY922 GSE19638 mcf7:141
Drug_Perturbations_from_GEO_up//Bisphenol A 6623 human GSE17624 sample 2662
Gene_Perturbations_from_GEO_down//Neurod1 Deficiency mouse GSE6030 sample 639
Disease_Perturbations_from_GEO_up//acute myocardial infarction DOID−9408 mouse GSE775 sample 1000
Gene_Perturbations_from_GEO_up//SIRT1 KD human GSE30486 sample 2353
Gene_Perturbations_from_GEO_down//CPSF6 KD human GSE42327 sample 2859
Drug_Perturbations_from_GEO_down//decitabine DB01262 human GSE29077 sample 2538
MCF7_Perturbations_from_GEO_down//doxorubicin GSE50650 mcf7:358
Gene_Perturbations_from_GEO_down//CTNNB1 KO mouse GSE7430 sample 1462
Gene_Perturbations_from_GEO_up//FGFR3 KD human GSE41035 sample 106
Gene_Perturbations_from_GEO_up//FGFR3 KD human GSE41035 sample 104
Ligand_Perturbations_from_GEO_up//estradiol human MCF−7 cells GDS4052 ligand:265
MCF7_Perturbations_from_GEO_up//estradiol GSE24592 mcf7:36
Drug_Perturbations_from_GEO_down//etanercept DB00005 human GSE11903 sample 2606
Drug_Perturbations_from_GEO_down//etanercept DB00005 human GSE11903 sample 2607
Drug_Perturbations_from_GEO_up//VANADIUM PENTOXIDE 14814 human GSE5339 sample 3558
Drug_Perturbations_from_GEO_up//HYPOCHLOROUS ACID 24341 mouse GSE15457 sample 3385
Gene_Perturbations_from_GEO_down//Myc KD mouse GSE30323 sample 2609
Gene_Perturbations_from_GEO_down//Apod OE mouse GSE28643 sample 1267
Drug_Perturbations_from_GEO_down//Retinoic acid 444795 human GSE23702 sample 3232
Ligand_Perturbations_from_GEO_down//All−trans−retinoic acid human NB4 APL cells (TG2−knockdown) GDS4180 ligand:196
Gene_Perturbations_from_GEO_down//AK8 knockdown human GSE19199 sample 1896
MCF7_Perturbations_from_GEO_up//E2 24 hrs GSE48989 mcf7:343
Drug_Perturbations_from_GEO_down//Cholestyramine DB01432 human GSE11223 sample 3294
Drug_Perturbations_from_GEO_down//NSC319726 5351307 human GSE35972 sample 2479
Disease_Perturbations_from_GEO_down//Acne C0702166 human GSE10432 sample 297
Drug_Perturbations_from_GEO_down//isotretinoin DB00982 human GSE10432 sample 2772
Gene_Perturbations_from_GEO_down//TGFB1I1 KD human GSE46448 sample 1213
Gene_Perturbations_from_GEO_down//TGFB1I1 KD human GSE46448 sample 1210
Drug_Perturbations_from_GEO_down//irinotecan DB00762 rat GSE11722 sample 2767
Ligand_Perturbations_from_GEO_up//interleukin−22 human Bronchial epithelial cells GDS3393 ligand:198
Gene_Perturbations_from_GEO_down//RNF40 KD human GSE38173 sample 1385
Gene_Perturbations_from_GEO_up//Dlx5 OE mouse GSE22381 sample 372
Gene_Perturbations_from_GEO_up//LMO3 KD human GSE41712 sample 1783
Gene_Perturbations_from_GEO_down//LAMP3 KD human GSE29009 sample 1538
Gene_Perturbations_from_GEO_down//LAMP3 KD human GSE29009 sample 1541
Gene_Perturbations_from_GEO_down//CDH1 OE human GSE32540 sample 2350
Gene_Perturbations_from_GEO_up//TGFB1I1 KD human GSE47203 sample 1825
Drug_Perturbations_from_GEO_up//doxycycline DB00254 human GSE41035 sample 3041
Gene_Perturbations_from_GEO_up//FGFR3 knock−down (shRNA 6−16, doxycycline−induced) human GSE41035 sample 1253

O

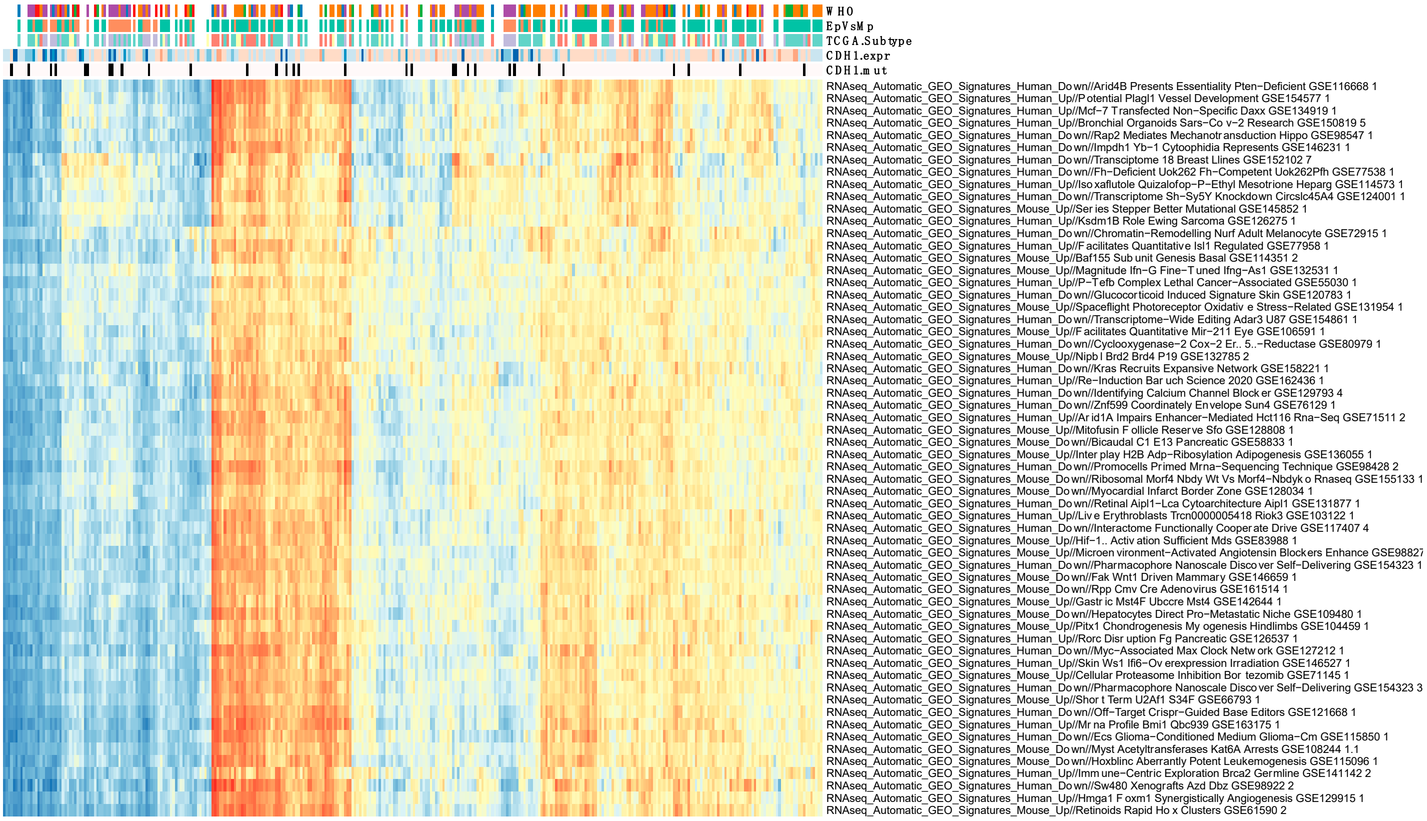

Figure S1 A–O. Gene enrichment analysis on genes correlated with CDH1 mRNA level in 483 GC cases from cBioPortal, Stomach Adenocarcinoma, Firehose Legacy Study. The analysis was performed as follows: a total of 124 gene set collections adding up to 147,592 gene sets were obtained from Enrichr at https://maayanlab.cloud/Enrichr/ (26). The collections were split into 13 functionally related groups (blocks **A–O**) and used to perform gene set variation analysis (GSVA) (27). Subsequently, differential enrichment analysis on gene correlation with CDH1 gene expression were performed using *limma* software tool package for each block (28). EP (epithelial phenotype); MP (mesenchymal phenotype).

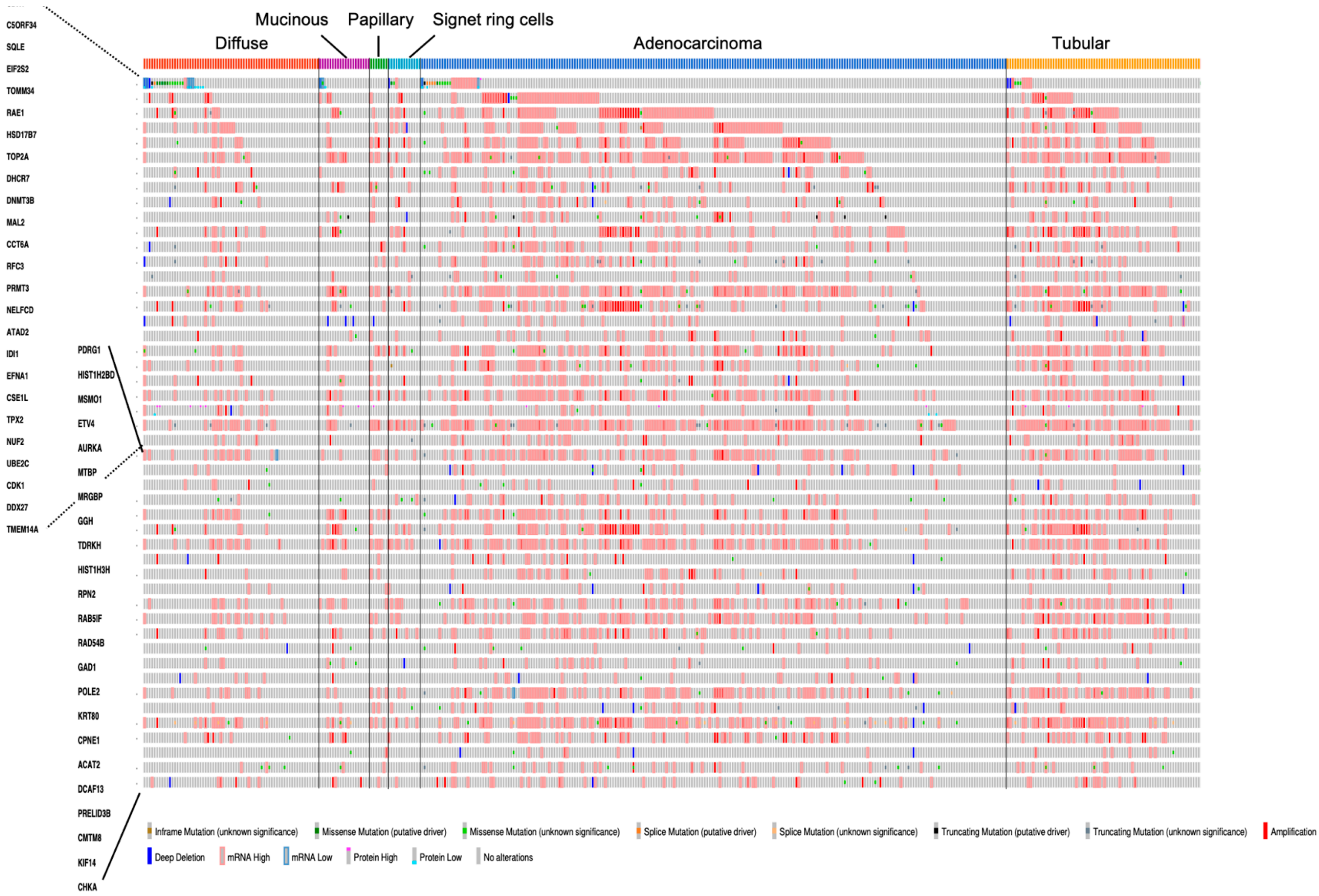

Figure S2. Representation of genomic data referred to 47 genes downregulated in CDH1-mutated compared to CDH1-wt GC cases. Genomic data of 47 of the 50 genes reported in Table 2, plus CDH1, in 415 GC cases divided into 6 subtypes (from cBioPortal, Stomach Adenocarcinoma, Firehose Legacy study). Genetic data of genes LINC00511, GPR89B and CKS1B were not available. Red-blu color scale indicates variation from higher to lower than diploid samples mRNA level (z-scores, RNA Seq V2 RSEM): light red, upregulated; light blu downregulated. Dark red, gained compared to diploid; dark blu, lost compared to diploid. Grey, invariant.

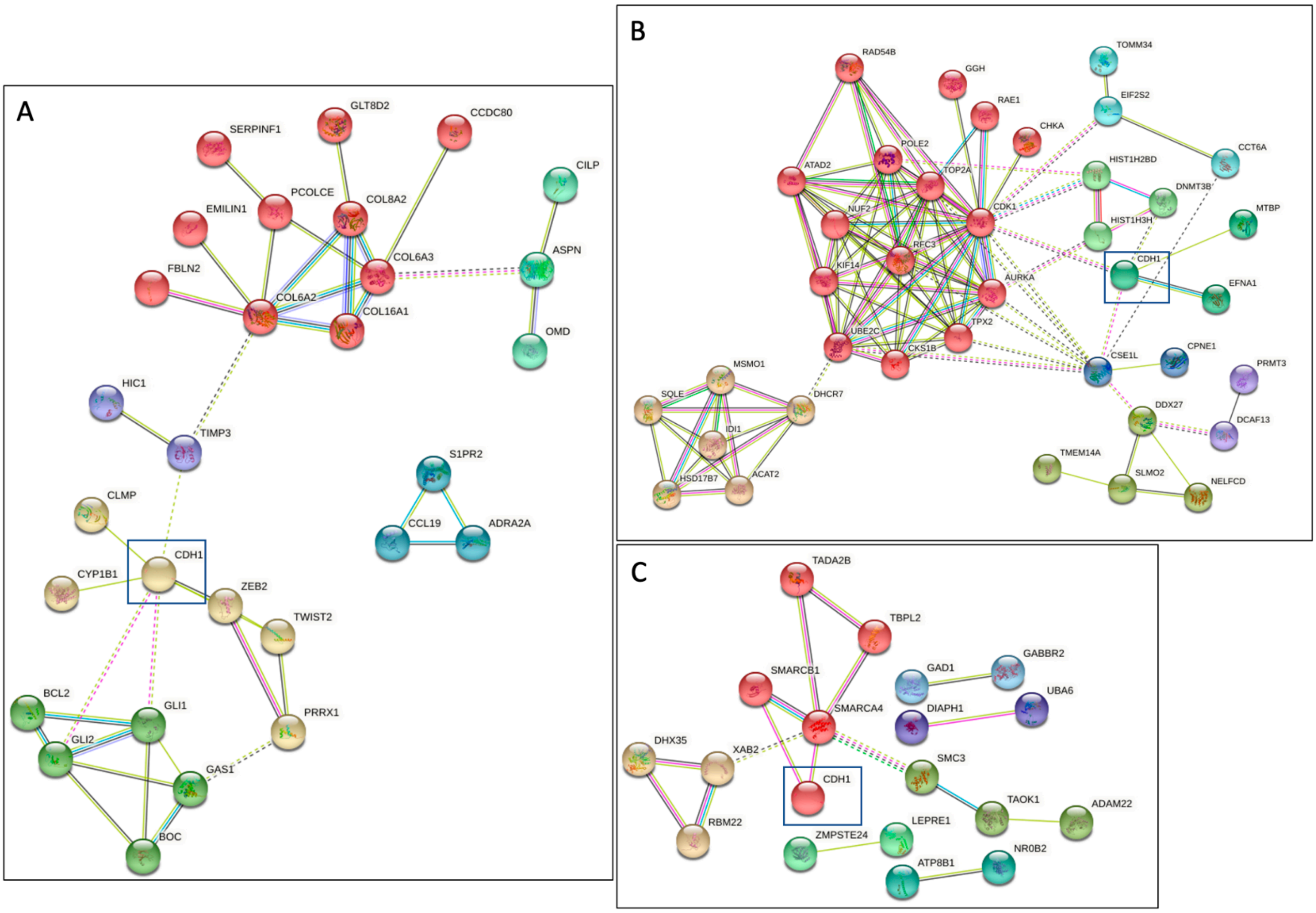

Figure S3 A–C. Protein interactomes of CDH1-associated transcriptional patterns. Interactomes of proteins (Table 1) upregulated in GC-mutated versus non-mutated GC cases (**A**), proteins (Table 2) down-regulated in GC-mutated versus non-mutated cases (**B**), proteins (Table 3) associated with CDH1 upregulation in GC-mutated versus non-mutated GC cases (**C**). Conditions applied using the software tool STRING: physical interactions; interaction score medium confidence; MCL clustering, inflation 3. Not connected nodes were removed. Clusters are identified by different colors. CDH1 is identified by a rectangle.

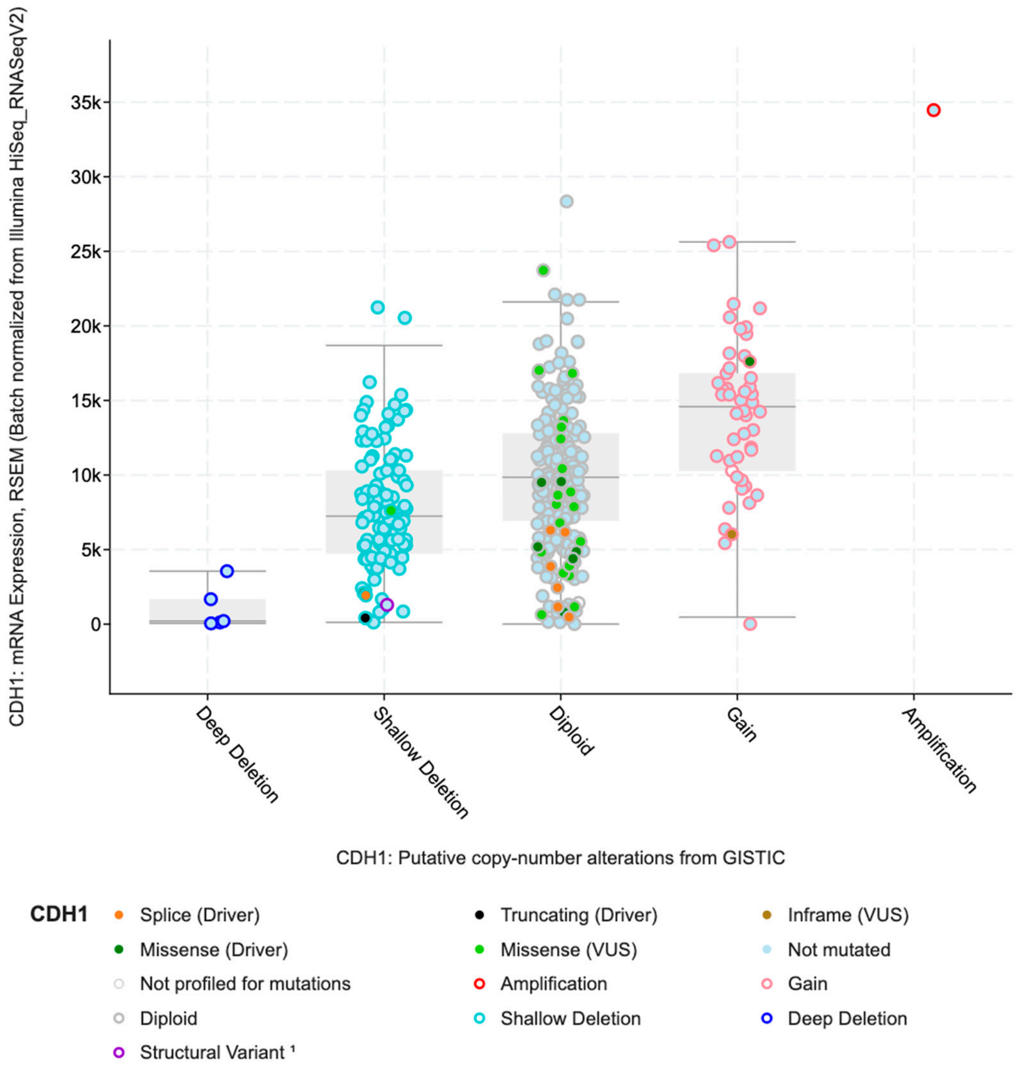

Figure S4. CDH1 mRNA levels and copy number status in 483 GC cases. The graph is reproduced from cBioPortal, Stomach Adenocarcinoma, Firehose Legacy Study.

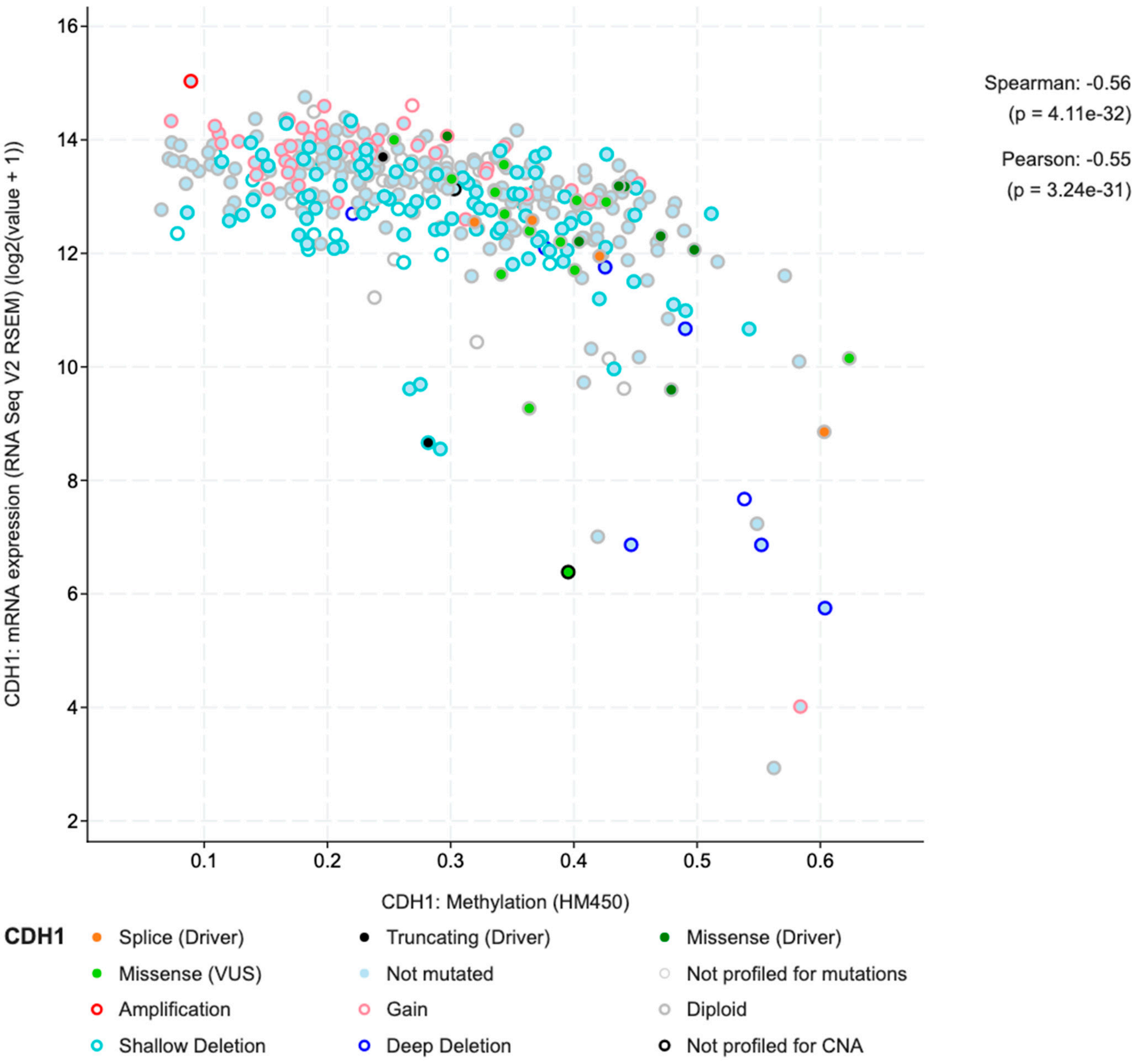

Figure S5. CDH1 transcript levels as a function of gene methylation in 483 GC cases. The graph is reproduced from cBioPortal, Stomach Adenocarcinoma, Firehose Legacy Study.

Table S1. Signaling pathways associated with upregulated and downregulated genes in CDH1-mutated versus non-mutated DGC (diffuse GC) according to the MSigDB database.

Gene Set Name	Description	Genes in Overlap	FDR q-value
Upregulated genes			
LEE_BMP2_TARGETS_UP [772]	Genes up-regulated in uterus upon knockout of BMP2	104	2.7 e-45
BOQUEST_STEM_CELL_UP [261]	Genes up-regulated in freshly isolated CD31- (stromal stem cells from adipose tissue) versus the CD31+ (non-stem) counterparts.	63	3.6 e-42
BENPORATH_ES_WITH_H3K27ME3 [1114]	Set 'H3K27 bound': genes posessing the trimethylated H3K27 (H3K27me3) mark in their promoters in human embryonic stem cells, as identified by ChIP on chip.	118	2.0 e-41
LIM_MAMMARY_STEM_CELL_UP [480]	Genes consistently up-regulated in mammary stem cells both in mouse and human species.	78	1.1 e-39
NABA_MATRISOME [1026]	Ensemble of genes encoding extracellular matrix and extracellular matrix-associated proteins	103	7.2 e-34
WEST_ADRENOCORTICAL_TUMOR_DN [544]	Down-regulated genes in pediatric adrenocortical tumors (ACT) compared to the normal tissue.	75	3.6 e-33
BENPORATH_SUZ12_TARGETS [1033]	Set 'Suz12 targets': genes identified by ChIP on chip as targets of the Polycomb protein SUZ12 in human embryonic stem cells.	100	1.3 e-31
MEISSNER_BRAIN_HCP_WITH_H3K4ME3_AND_H3 _H3K27ME3 [1073]	Genes with high-CpG-density promoters (HCP) bearing histone H3 dimethylation at K4 (H3K4me2) and trimethylation at K27 (H3K27me3) in brain.	101	4.9 e-31
LINDGREN_BLADDER_CANCER_CLUSTER_2B [389]	Genes specifically up-regulated in Cluster IIb of urothelial cell carcinom (UCC) tumors.	57	2.4 e-26
LIU_PROSTATE_CANCER_DN [493]	Genes down-regulated in prostate cancer samples.	62	4.6 e-25
CHICAS_RB1_TARGETS_CONFLUENT [567]	Genes up-regulated in confluent IMR90 cells (fibroblast) after knockdown of RB1 by RNAi.	66	7.4 e-25
BENPORATH_EED_TARGETS [1058]	Set 'Eed targets': genes identified by ChIP on chip as targets of the Polycomb protein EED in human embryonic stem cells.	90	2.5 e-24
SMID_BREAST_CANCER_NORMAL_LIKE_UP [484]	Genes up-regulated in the normal-like subtype of breast cancer.	60	5.7 e-24
CHEN_METABOLIC_SYNDROM_NETWORK [1241]	Genes forming the macrophage-enriched metabolic network (MEMN) claimed to have a causal relationship with the metabolic syndrom traits.	96	4.8 e-23
GOBERT_OLIGODENDROCYTE_DIFFERENTIATION ION_DN [1081]	Genes down-regulated during differentiation of Oli-Neu cells (oligodendroglial precursor) in response to PD174265.	88	1.6 e-22
ONDER_CDH1_TARGETS_2_UP [257]	Genes up-regulated in HMLE cells (immortalized nontransformed mammary epithelium) after E-cadhedrin (CDH1) [GeneID=999] knockdown by RNAi.	43	4.7 e-22
SWEET_LUNG_CANCER_KRAS_DN [422]	Genes down-regulated in the Kras2LA mouse lung cancer model with mutated KRAS.	53	2.2 e-21
BENPORATH_PRC2_TARGETS [649]	Set 'PRC2 targets': Polycomb Repression Complex 2 (PRC) targets; identified by ChIP on chip on human embryonic stem cells as genes that: posess the trimethylated H3K27 mark in their promoters and are bound by SUZ12 and EED [GeneID=8726] Polycomb proteins.	65	5.3 e-21
NABA_CORE_MATRISOME [275]	Ensemble of genes encoding core extracellular matrix including ECM glycoproteins, collagens and proteoglycans	42	5.3 e-20
RIGGI_EWING_SARCOMA_PROGENITOR_UP [435]	Genes up-regulated in mesenchymal stem cells (MSC) engineered to express EWS-FLI1 fusion protein.	51	3.1 e-19
Downregulated genes			
FISCHER_DREAM_TARGETS [968]	Target genes of the DREAM complex.	178	4.8 e-146
KINSEY_TARGETS_OF_EWSR1_FLII_FUSION_UP [1290]	Genes up-regulated in TC71 and EWS502 cells (Ewing's sarcoma) by EWSR1-FLI1 as inferred from RNAi knockdown of this fusion protein.	176	4.3 e-121
DODD_NASOPHARYNGEAL_CARCINOMA_DN [1408]	Genes down-regulated in nasopharyngeal carcinoma (NPC) compared to the normal tissue.	178	6.2 e-117
PUJANA_BRCA1_PCC_NETWORK [1628]	Genes constituting the BRCA1-PCC network of transcripts whose expression positively correlated (Pearson correlation coefficient, PCC >= 0.4) with that of BRCA1 across a compendium of normal tissues.	185	8.1 e-114
GOBERT_OLIGODENDROCYTE_DIFFERENTIATION ION_UP [571]	Genes up-regulated during later stage of differentiation of Oli-Neu cells (oligodendroglial precursor) in response to PD174265	124	1.6 e-108
PUJANA_CHEK2_PCC_NETWORK [762]	Genes constituting the CHEK2-PCC network of transcripts whose expression positively correlates (Pearson correlation coefficient, PCC >= 0.4) with that of CHEK2	127	4.8 e-96
DUTERTRE_ESTRADIOL_RESPONSE_24HR_UP [323]	Genes up-regulated in MCF7 cells (breast cancer) at 24 h of estradiol treatment.	93	1.3 e-92

SHEDDEN_LUNG_CANCER_POOR_SURVIVAL_A6 [459]	Cluster 6 of method A: up-regulation of these genes in patients with non-small cell lung cancer (NSCLC) predicts poor survival outcome.	104	3.2 e-92
MARSON_BOUND_BY_E2F4_UNSTIMULATED [692]	Genes with promoters bound by E2F4 in unstimulated hybridoma cells.	119	2.2 e-91
MEBARKI_HCC_PROGENITOR_FZD8CRD_UP [586]	Transcriptome of human HepaRG hepatocellular carcinoma liver progenitors in responses to a WNT3A-enriched microenvironment and dissection of pathways dependent on _-catenin and/or blocked by the SFRP-like Wnt inhibitor FZD8_CRD.	112	5 .0 e-91
KOBAYASHI_EGFR_SIGNALING_24HR_DN [252]	Genes down-regulated in H1975 cells (non-small cell lung cancer, NSCLC) resistant to gefitinib after treatment with EGFR inhibitor CL-387785 for 24h.	81	9.0 e-85
BERENJENO_TRANSFORMED_BY_RHOA_UP [551]	Genes up-regulated in NIH3T3 cells (fibroblasts) transformed by expression of contitutively active (Q63L) form of RHOA off plasmid vector.	101	1.0 e-79
SOTIRIOU_BREAST_CANCER_GRADE_1_VS_3_UP [154]	Up-regulated genes whose expression correlated with histologic grade of invasive breast cancer tumors: comparison of grade 1 vs grade 3.	66	1.4 e-78
REACTOME_CELL_CYCLE [693]	Cell Cycle	108	7.7 e-78
ROSTY_CERVICAL_CANCER_PROLIFERATION_CL_CLUSTER [140]	The 'Cervical Cancer Proliferation Cluster' (CCPC): genes whose expression in cervical carcinoma positively correlates with that of the HPV E6 and E7 oncogenes; they are also differentially expressed according to disease outcome.	62	7.6 e-75
REACTOME_CELL_CYCLE_MITOTIC [561]	Cell Cycle, Mitotic	97	6.0 e-74
CAIRO_HEPATOBLASTOMA_CLASSES_UP [612]	Genes up-regulated in robust Cluster 2 (rC2) of hepatoblastoma samples compared to those in the robust Cluster 1 (rC1).	99	1.1 e-72
LEE_BMP2_TARGETS_DN [898]	Genes down-regulated in uterus upon knockout of BMP2	111	3.8 e-69
PUJANA_BRCA2_PCC_NETWORK [424]	Genes constituting the BRCA2-PCC network of transcripts whose expression positively correlated (Pearson correlation coefficient, PCC >= 0.4) with that of BRCA2 across a compendium of normal tissues.	83	1.9 e-67
FLORIO_NEOCORTEX_BASAL_RADIAL_GLIA_DN [190]	Genes down-regulated in basal radial glia (bRG) relative to apical radial glia (aRG), and up-regulated in both aRG and bRG relative to neurons.	63	1.6 e-66

Table S2. Listing of small drug compounds targeting the top 20 genes found to be up- or down-regulated in CDH1-mutated versus non-mutated GCs, including DGC (diffuse GC).

Rank	Score*	Perturbation	Cell-line	Dose	Time
In all GC					
1	0.1667	FIT	HCC515	10.0μM	24.0h
2	0.1667	GR 127935 hydrochloride	PC3	10.0μM	24.0h
3	0.1667	AMIODARONE HYDROCHLORIDE	A375	10.0μM	24.0h
4	0.1667	GF-109203X	HA1E	12.12μM	24.0h
5	0.1667	Akti-1/2	VCAP	9.1μM	6.0h
6	0.1667	TG101348	VCAP	11.1μM	6.0h
7	0.1667	BRD-K54256913	VCAP	10.0μM	6.0h
8	0.1667	BRD-K94390040	VCAP	10.0μM	6.0h
9	0.1667	isoquercitrin	PC3	10.0μM	24.0h
10	0.1667	trichostatin A	SKB	10.0μM	24.0h
11	0.1667	BRD-K94832621	A549	10.0μM	24.0h
12	0.1667	BRD-A10420615	MCF7	10.0μM	24.0h
13	0.1667	BRD-K11634954	A549	10.0μM	24.0h
14	0.1667	sirolimus	HT29	10.0μM	6.0h
In DGC					
1	0.2222	BRD-K55722623	HEPG2	10.0μM	6.0h
2	0.2222	BRD-K13169950	ASC	10.0μM	24.0h
3	0.1667	AY 9944	HA1E	10.0μM	24.0h
4	0.1667	N9-isoproplyolomoucine	A673	122.55μM	6.0h
5	0.1667	Salermide	HT29	120.0μM	24.0h
6	0.1667	Arachidonyl trifluoro-methyl ketone	HT29	80.0μM	24.0h
7	0.1667	piperlongmMine (HPLC)	HT29	10.0μM	24.0h
8	0.1667	piperlongmMine (HPLC)	MCF7	10.0μM	24.0h
9	0.1667	ABT-751	MCF7	10.0μM	24.0h
10	0.1667	BRD-K05649647	NCIH1836	20.0μM	6.0h
11	0.1667	Emetine Dihydrochloride Hydrate (74)	NCIH596	0.63μM	6.0h
12	0.1667	Parthenolide	SW948	10.0μM	6.0h
13	0.1667	BRD-K70871370	HCC515	10.0μM	6.0h
14	0.1667	BRD-K18726304	HEPG2	10.0μM	6.0h

*A score is the overlap between the input differentially expressed genes and the signature differentially expressed genes divided by the effective input, reciprocally adopted as input upregulated genes intersected with the downregulated genes of the datasets and input downregulatedgenes intersected with the corresponding upregulated genes. The analysis was performed through L1000CDS^2 at https://maayanlab.cloud/L1000CDS2/#/index.

Table S3. Listing of best combinations of small drug compounds targeting the genes found to be up- or down-regulated in CDH1-mutated versus non-mutated GCs and DGC (diffuse GC).

Rank	Score*	Combination+	
In all GC			
1	0.3333	4. GF-109203X	16. BRD-K37392901
2	0.3333	7. BRD-K54256913	16. BRD-K37392901
3	0.3333	13. BRD-K11634954	16. BRD-K37392901
4	0.3333	4. GF-109203X	22. radicicol
5	0.3333	7. BRD-K54256913	22. radicicol
6	0.3333	13. BRD-K11634954	22. radicicol
7	0.3333	19. radicicol	22. radicicol
8	0.3333	16. BRD-K37392901	23. nintedanib
9	0.3333	19. radicicol	23. nintedanib
10	0.3333	22. radicicol	23. nintedanib
11	0.3	1. FIT	16. BRD-K37392901
12	0.3	2. GR 127935 hydrochloride	16. BRD-K37392901
13	0.3	3. AMIODARONE HYDROCHLORIDE	16. BRD-K37392901
14	0.3	5. Akti-1/2	16. BRD-K37392901
In DGC			
1	0.3889	1. BRD-K55722623	2. BRD-K13169950
2	0.3889	2. BRD-K13169950	5. Salermide
3	0.3889	2. BRD-K13169950	9. ABT-751
4	0.3333	1. BRD-K55722623	3. AY 9944
5	0.3333	2. BRD-K13169950	3. AY 9944
6	0.3333	2. BRD-K13169950	4. N9-isoproplyolomoucine
7	0.3333	2. BRD-K13169950	6. Arachidonyl trifluoro-methyl ketone
8	0.3333	1. BRD-K55722623	7. piperlongumine (HPLC)
9	0.3333	3. AY 9944	7. piperlongumine (HPLC)
10	0.3333	1. BRD-K55722623	8. piperlongumine (HPLC)
11	0.3333	3. AY 9944	8. piperlongumine (HPLC)
12	0.3333	3. AY 9944	9. ABT-751
13	0.3333	2. BRD-K13169950	10. BRD-K05649647
14	0.3333	5. Salermide	10. BRD-K05649647

*A score is the overlap between the input differentially expressed genes and the signature differentially expressed genes divided by the effective input, reciprocally adopted as input upregulated genes intersected with the downregulated genes of the datasets and input downregulatedgenes intersected with the corresponding upregulated genes. The analysis was performed through L1000CDS^2 at https://maayanlab.cloud/L1000CDS2/#/index.

+The number next to a drug name refers to the position of the individual treatment.
